# Transcriptome sequencing and analysis of zinc-uptake-related genes in *Trichophyton mentagrophytes*

**DOI:** 10.1186/s12864-017-4284-3

**Published:** 2017-11-21

**Authors:** Xinke Zhang, Pengxiu Dai, Yongping Gao, Xiaowen Gong, Hao Cui, Yipeng Jin, Yihua Zhang

**Affiliations:** 1 The College of Veterinary Medicine of the Northwest Agriculture and Forestry University, No. 3 Taicheng Road, Yangling, Shaanxi People’s Republic of China; 2 Clinical Department, College of Veterinary Medicine, China Agricultural University, Beijing, People’s Republic of China

**Keywords:** *Trichophyton mentagrophytes*, Transcriptome sequencing, Functional annotation, Zinc uptake, Zinc-responsive activating factor

## Abstract

**Background:**

*Trichophyton mentagrophytes* is an important zoonotic dermatophytic (ringworm) pathogen; causing severe skin infection in humans and other animals worldwide. Fortunately, commonly used fungal skin disease prevention and treatment measures are relatively simple. However, *T. mentagrophytes* is primarily studied at the epidemiology and drug efficacy research levels, yet current study has been unable to meet the needs of clinical medicine.

Zinc is a crucial trace element for the growth and reproduction of fungi and other microorganisms. The metal ions coordinate within a variety of proteins to form zinc finger proteins, which perform many vital biological functions. Zinc transport regulatory networks have not been resolved in *T. mentagrophytes*. The *T. mentagrophytes* transcriptome will allow us to discover new genes, particularly those genes involved in zinc uptake.

**Result:**

We found *T. mentagrophytes* growth to be restricted by zinc deficiency; natural *T. mentagrophytes* growth requires zinc ions. *T. Mentagrophytes* must acquire zinc ions for growth and development*.*

The transcriptome of *T. mentagrophytes* was sequenced by using Illumina HiSeq™ 2000 technology and the de novo assembly of the transcriptome was performed by using the Trinity method, and functional annotation was analyzed. We got 10,751 unigenes. The growth of *T. mentagrophytes* is severely inhibited and there were many genes showing significant up regulation and down regulation respectively in *T. mentagrophytes* when zinc deficiency. Zinc deficiency can affect the expression of multiple genes of *T. mentagrophytes.* The effect of the zinc deficiency could be recovered in the normal medium. And we finally found the zinc-responsive activating factor (*ZafA*) and speculated that 4 unigenes are zinc transporters. We knocked *ZafA* gene by ATMT transformation in *T. mentagrophytes*, the result showed that *ZafA* gene is very important for the growth and the generation of conidia in *T. mentagrophytes*. The expression of 4 zinc transporter genes is potentially regulated by the zinc-responsive activating factor. The data of this study is also sufficient to be used as a support to study *T. mentagrophytes*.

**Conclusion:**

We reported the first large transcriptome study carried out in *T. mentagrophytes* where we have compared physiological and transcriptional responses to zinc deficiency, and analyzed the expression of genes involved in zinc uptake. The study also produced high-resolution digital profiles of global genes expression relating to *T. mentagrophytes* growth.

**Electronic supplementary material:**

The online version of this article (10.1186/s12864-017-4284-3) contains supplementary material, which is available to authorized users.

## Background

*Trichophyton mentagrophytes* is an important zoonotic dermatophytic (ringworm) pathogen that can cause severe skin infections in humans and other animals, seriously threatening to human health and animal husbandry [1, 2].

Particular nutrients play an extremely important role during *T. mentagrophytes* invasion; these elements include zinc, iron, nitrogen, and selenium [3]. Among these, zinc is a crucial trace element, which often coordinates within a variety of proteins to form zinc finger proteins, which perform many vital biological functions. Although zinc is essential for fungi, it can also be toxic. When the intracellular zinc level rises to some critical level, the zinc ions can affect other important physiological processes [4]. Therefore, fungi have successfully evolved zinc transporter systems to maintain a homeostatic balance of zinc ions for survival and virulence.

Zinc transporter system expression in the model fungus *Saccharomyces cerevisiae* is primarily regulated by the C_2_H_2_-type zinc finger transcription factor *Zap1* at the transcriptional level [5, 6]. Studies have shown that various fungi can secrete functionally similar zinc finger transcriptional factors. For example in the fungi, *Aspergillus fumigatus, Candida albicans*, and *Cryptococcus gattii*, mutations in similar zinc transport mechanism genes stop growth and development, and can even cause loss of virulence [7–11]. *T. mentagrophytes* expresses various zinc finger proteins; of prime importance for growth and virulence is the exocrine zinc finger protein. Perhaps most representative are the zinc metalloproteinases, which can digest and absorb nutrients, and invade the body cuticle [12]. Previous research has shown that metalloproteinase gene mutations can affect *T. mentagrophytes* virulence at different levels [13]. At the same time, only if zinc metalloproteinase combines zinc element will it be able to exhibit biological activity**.** We speculated that *T. mentagrophytes* has a C_2_H_2_-type zinc finger transcription factor that can serve as an upstream regulator in the absorption of zinc.

The *T. mentagrophytes* zinc transport regulation network has not been determined. Our study sequenced the *T. mentagrophytes* transcriptome using Illumina HiSeq™ 2000 technology, a de novo assembly of the transcriptome was performed using the Trinity method, and we performed functional annotation analysis. This allowed us to produce high-resolution digital profiles of global gene expression relating to *T. mentagrophytes* growth. The *T. mentagrophytes* transcriptome will be characterized further, and zinc-uptake-related gene families will be systematically explored as discovered, in future work.

## Methods

### Fungal culture and RNA extraction

The *T. mentagrophytes* wild-type strain ATCC 28185 (a gift from Ruoyu Li, Peking University First Hospital, China) was maintained at 28 °C on solid Sabouraud dextrose medium (SDA) for 14 days. Five mL of sterile saline was used to wash off spores so as to collect fungus liquid. The fungus liquid concentration was adjusted to 10^8^ CFU/mL by cell count plate. SDA with 1 mM EDTA was supplemented to generate zinc deficient SDA, named SDA-Zn (zinc ions have been chelated)**.** The 150-μL fungus liquid was inoculated to SDA (sufficient zinc ions, grouped into Norm) and SDA-Zn with 200, 400, 600, and 1000 μM of zinc sulfate (grouped into Zn200, Zn400, Zn600, and Zn1000) respectively. Culture conditions were 28 °C for 14 days.

Total RNA was extracted using TRIzol® reagent (Invitrogen, USA) following the manufacturer’s protocol, and DNase Ι (Takara, Japan) was used to remove genomic DNA. Integrity and size distributions were checked using an Agilent 2100 (Agilent, USA) with an RNA integrity number (RIN: 8.0) and GE Image Quant 350 (GE Healthcare, USA).

### cDNA library construction and Illumina sequencing

The extracted RNA samples (Norm, Zn400, and Zn1000) were used for cDNA synthesis. Poly(A) mRNA was enriched by Oligo (dT) beads (Qiagen, German). Next, the enriched mRNA was fragmented and reverse transcribed into first-strand cDNAs with random hexamers. Use DNA polymerase I (Thermo Fisher Scientific, USA), RNase H, dNTP, and buffer to synthesize second-strand cDNA. Then using QiaQuick PCR extraction kit (Qiagen, German) to purify the cDNA fragments, and the cDNA fragments were ligated to Illumina sequencing adapters. The ligation products were size selected by agarose gel electrophoresis, PCR amplified, and sequenced using Illumina HiSeq™ 2000 by Gene De novo Biotechnology Co (Guangzhou, China). Sequence data were deposited at the NCBI Short Read Archive database (https://trace.ncbi.nlm.nih.gov/Traces/sra/sra.cgi?cmd=search_obj&m=&s=&term=SRR5097230&go=Search) under the accession numbers SRR5097135, SRR5097227, SRR5097226, SRR5097228 (Norm), SRR5097229, SRR5097230, SRR5097231, SRR5097232 (Zn10 00), and SRR5895930 (Zn400).

### De novo assembly and functional annotation

High quality, clean reads for the assembly library were generated by filtering according to the following rules: reads containing adapters, more than 10% of unknown nucleotides (N) and more than 50% low quality (Q-value ≤10) bases were removed. The quality-filtered reads obtained were then de novo assembled into contigs by the Trinity Program [14]. Trinity is a modular method and software package, which combines three components: *Inchworm, Chrysalis*, and *Butterfly*. Initially, *Inchworm* assembles reads, resulting in a collection of linear contigs. Next, *Chrysalis* clusters related contigs, and then builds de Bruijn graphs for each cluster of related contigs. Finally, *Butterfly* analyzes the paths and outputs one linear sequence and ultimately generates unigenes. We used the BLASTx program (https://blast.ncbi.nlm.nih.gov/Blast.cgi) with an E-value threshold of 1 × 10^−5^ to obtain protein functional annotations, by aligning our unigenes to protein sequences from NCBI Nr (non-redundant protein database, https://blast.ncbi.nlm.nih.gov/Blast.cgi), Swiss-Prot (annotated protein sequence database, http://www.expasy.org/), KEGG (Kyoto encyclopedia of genes and genomes, http://www.genome.jp/kegg/), and COG (clusters of orthologous groups of protein, https://www.ncbi.nlm.nih.gov/COG/). The Blast2GO program [15] was used to obtain gene ontology (GO) annotation of our unigenes from Nr annotation, and then WEGO software [16] was used to perform GO functional classifications. KEGG is a major public pathway-related database [17] with which one is able to analyze gene products within the context of metabolic and cellular processes.

### Identification of Differentially Expressed Genes (DEGs)

The RPKM (reads per kb per million reads) was used to calculate and normalize the number of unique-match reads. The formula follows: RPKM = (1000,000 × C)/(N × L/1000), with RPKM set as the expression of unigene A, C as the number of reads that are uniquely mapped to unigene A, N as the total number of reads that are uniquely mapped to all unigenes, and L as the length (base number) of unigene A. The RPKM measure can provide normalized values of gene expression to enable transcript comparisons between Norm, Zn400, and Zn1000. We used the edgeR package (https://bioconductor.org/packages/release/bioc/html/edgeR.html) to identify differentially expressed genes across samples. We specified |log2FC| > 1 with the false discovery rate (FDR) < 0.05, as the thresholds necessary to determine significant differences in gene expression between Norm, Zn400, and Zn1000. Differentially expressed genes (DEGs) were then subjected to GO functional and KEGG pathway enrichment analyses. GO enrichment analysis provides all GO terms that are significantly enriched in DEGs compared with the genome background, and filter DEGs corresponding to biological function. Initially all DEGs are mapped to GO terms in the Gene Ontology database (http://geneontology.org/), gene numbers are calculated for every term, and significantly enriched GO terms in DEGs compared with the genome background are defined by a hypergeometric test. The *P*-value formula is:

$$ P=1-\sum \limits_{i=0}^{m-1}\frac{\left({}_i^M\right)\left({}_{n-i}^{N-M}\right)}{\left({}_n^N\right)} $$

Here N is the number of all genes with GO annotation; M is the number of all genes that are annotated to the certain GO terms; n is the number of DEGs in N; m is the number of DEGs in M. The calculated *p*-value then goes through FDR correction, taking FDR ≤ 0.05 as a threshold. GO terms meeting this condition are defined as significantly enriched GO terms in DEGs. Our analysis successfully recognized the putative biological functions of our DEGs.

Genes usually interact with each other to play roles in particular biological functions. Pathway-based analysis helps to further determine genes’ biological functions. KEGG is the major public pathway-related database [17]. Pathway enrichment analysis identifies significantly enriched metabolic pathways or signal transduction pathways in DEGs. The significance formula is the same as that in GO analysis. Here N is the number of all genes with KEGG annotation, M is the number of all genes annotated to specific pathways, n is the number of DEGs in N, and m is the number of DEGs in M. The calculated p-value then goes through FDR Correction, taking FDR ≤ 0.05 as a threshold. Pathways meeting this condition are defined as significantly enriched pathways in those DEGs.

### Validation of differential expression using qRT-PCR

Total RNA was extracted as described above, and cDNA was generated from total RNA. Primers for quantitative real time PCR (qRT-PCR) were designed using Primer Premier 6.0 software (Premier, Canada), and synthesized by Gene De novo Biotechnology Co (Guangzhou, China). All primers are shown in Additional file 1. The 18S gene was used as an internal control. qRT-PCR was performed on a Step One Plus™ Real-Time PCR System (Thermo Fisher Scientific, USA). Each 20-μL reaction mixture contained 10 μL of Maxima SYBR Green/ROX qPCR Master Mix (2X) (Thermo Scientific, USA), 0.3 μL of each primer (10 μM), 0.8 μL of cDNA, and 8.6 μL of nuclease-free water. The qRT-PCR run protocol was as follows: 95 °C, 10 min; followed by 40 cycles of 95 °C, 15 s; 60 °C, 30 s; and 72 °C, 15 s in 96-well optical reaction plates. Three biological replicates with three technical replicates for each value determined the Ct values. Expression levels of the tested reference genes were determined by Ct values and calculated by2^-△△Ct.

### Construction of transformation vectors and ATMT transformation

The binary vector pDHt*/ZafA::hph* used for site-directed mutagenesis was constructed by reorganizing the hygromycin B resistance gene (*hph*) of plasmid pAN7–1, the left and right flanking sequences of the *ZafA* gene of *T.mentagrophytes* simultaneously into *XhoI/HindIII* digested plasmid *pDHt/SK* (a gift from Dr. K. J. Kwon-Chung). The *ZafA* gene was knocked by Agrobactirium tumfacience mediated-transformant (ATMT) in *T. mentagrophytes*. The two pairs of primers (*ZafA*-F: CCAGACTGAAGGTGCTAAG, *ZafA*-R: CCTGTTAGTATCGTCGTGTT; *hph*-F: TACATCCATACTCCATCCTTC, *hph-R*: CGGCATCTACTCTATTCCTT) designed by *ZafA* gene fragment disrupted and *hph* gene fragment were used to verified *ZafA* gene mutant strain, and its amplification length is 400 and 1200 bp, respectively.

## Results

### Effect of zinc deficiency on the growth of *T. mentagrophytes*

**Fig. 1:**
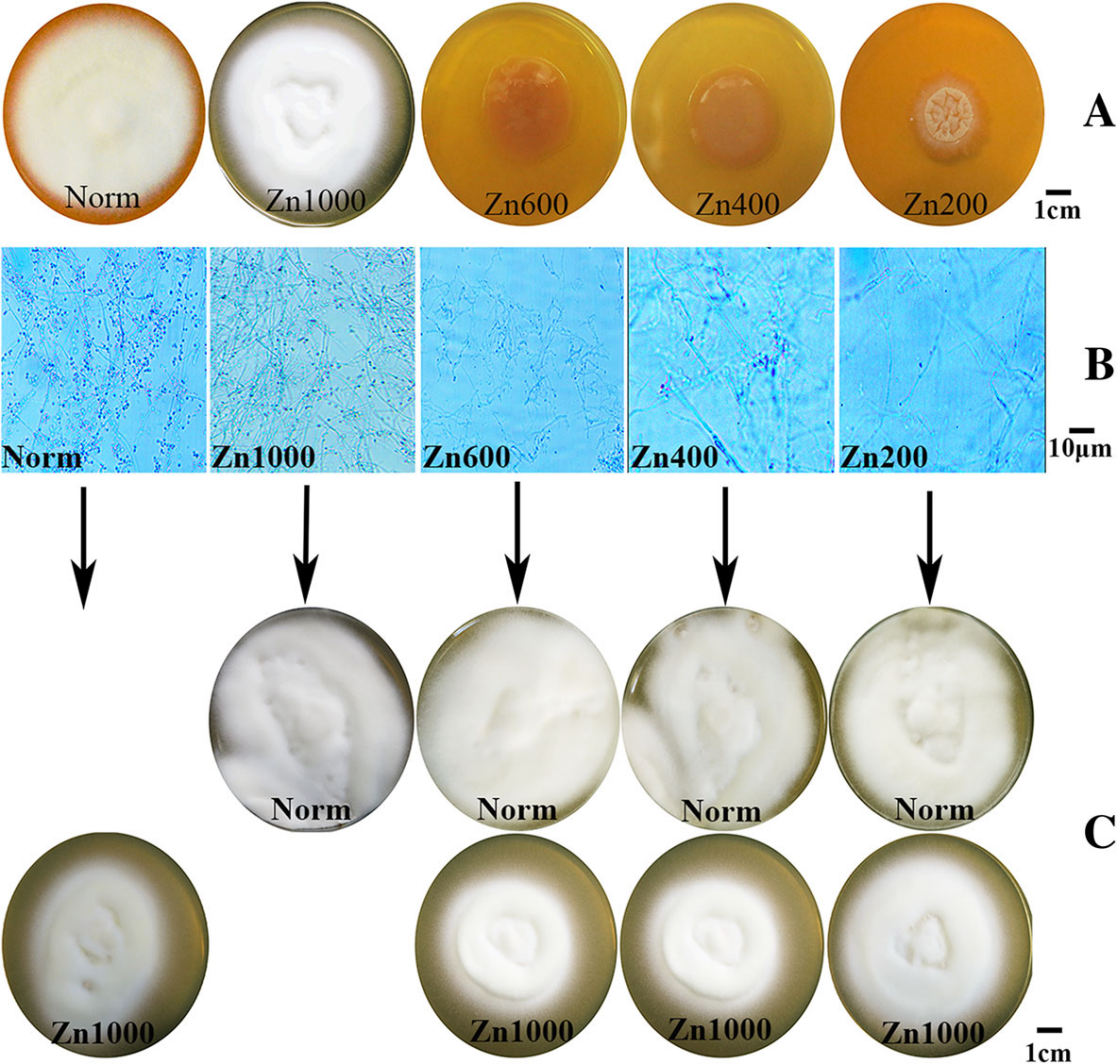
**a** The growth situation of *T. mentagrophytes* in 5 groups. SDA with 1 mM EDTA was supplemented to generate zinc deficient SDA, named SDA-Zn (zinc ions have been chelated)**.** The 150-μL fungus liquid was inoculated to SDA (sufficient zinc ions, grouped into Norm) and SDA-Zn with 200, 400, 600, and 1000 μM of zinc sulfate (grouped into Zn200, Zn400, Zn600, and Zn1000) respectively. *T. mentagrophytes* can grow well on SDA in Norm and Zn1000, the growth of Zn200、Zn400 and Zn600 *T. mentagrophyte* was inhibited, especially in Zn200. **b** The each group was stained by lacto phenol cotton blue. Norm and Zn1000 mycelium grow well and with numerous round microconidia. A small number of microconidia and mycelium can be observed in Zn600, and even fewer in Zn400. We could not detect favorable microconidia and the mycelium was particularly weak in Zn200. **c** *T. mentagrophytes* from Zn200, Zn400 and Zn600 were inoculated into normal medium and Zn1000 medium, the *T. mentagrophytes* growth traits returned to normal, *T. mentagrophytes* can grow well on SDA in Norm and Zn1000

The *T. mentagrophytes* cells of five groups were maintained at 28 °C for 14 days. We found *T. mentagrophytes* can grow well on SDA in Norm, presenting a white colony with fluffy, fine mycelium on its surface. The colony morphology of the Zn1000 group was not significantly different from that of the Norm group, but the growth rate was slower. In contrast, with the decreasing zinc ion concentration in its medium, the growth of Zn400 and Zn600 *T. mentagrophytes* was severely inhibited, with pale-yellow mucus-covered colonies in which mycelium could not be seen. The growth of Zn200 *T. mentagrophytes* was most seriously inhibited, with the colony appearing to be folded over (Fig. 1a).

Upon microscopy Norm and Zn1000 mycelium can be seen to grow well, with numerous round microconidia in grape-like clusters. A small number of microconidia and mycelium can be observed in Zn600, and even fewer in Zn400. However, we could not detect microconidia in Zn200 at all, and its mycelium were particularly weak (Fig. 1b).

*T. mentagrophytes* from Zn200, Zn400 and Zn600 were then inoculated into normal medium and Zn1000 medium, and the *T. mentagrophytes* growth traits returned to normal (Fig. 1c).

These results collectively indicate that when *T. mentagrophytes* cells grow in zinc-deficient conditions growth status is adversely affected, showing that zinc is very important for the growth of *T. mentagrophytes*.

### De novo assembly and sequence annotation

A total of 36,793,459 raw reads and 36,227,708 quality filtered (clean) reads were obtained from the Norm library. We obtained 30,028,768 raw reads and 29,576,580 quality filtered (clean) reads from Zn1000. And in Zn400, we obtained 47,306,862 raw reads and 46,394,758 quality filtered (clean) reads. The saturation curves shown depict the detected number of genes that tend to be saturated (Additional file 2). The Q20 percentages (percentage of sequences with sequencing error rates) of the three libraries, Norm, Zn1000, and Zn400, were 97.69, 97.72, and 94.58% respectively, and the GC content ranged from 51.52 to 52.83%. All clean reads were pooled together and then de novo assembled by Trinity. The assembly produced a substantial number of contigs and 10,751 unigenes.

These unigenes were annotated using the Nr, Swiss-Prot, KEGG, and KOG databases. A final number of 9593, 6113, 3765, and 5172 unigenes had matches in the Nr, Swiss-Prot, KEGG, and KOG databases, respectively (Additional file 3). Up to 89.23% of all machine annotated unigenes showed similarity to known proteins in the Nr database. Additionally, the unigenes were searched against the Nr database using BLASTx, and homologous sequences and species identification were ascertained. The five highest number of homologous sequences corresponding to particular species follows: 24.86% *Trichophyton equinum* CBS 127.97, 23.98% *Trichophyton tonsurans* CBS 112818, 10.40% *Trichophyton interdigitale* H6, 6.60% *Trichophyton rubrum* CBS 118892, and 4.35% *Microsporum gypseum* CBS 118893.

**Fig. 2:**
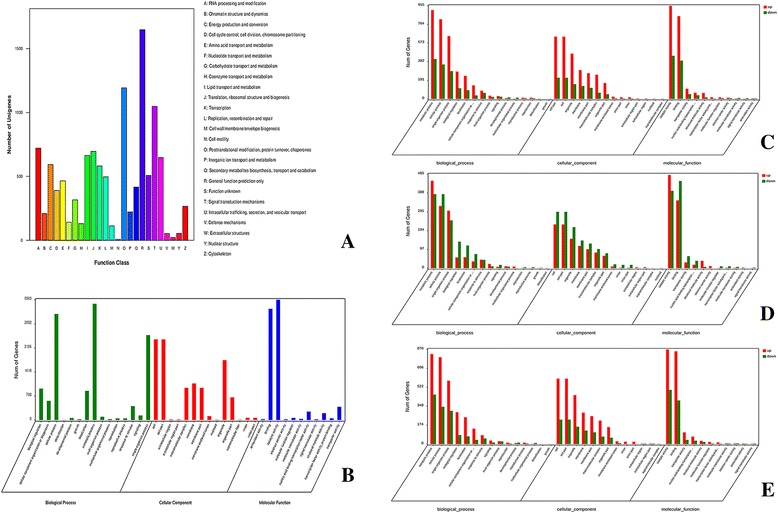
**a** The KOG classification of unigenes, 5172 unigenes (48.11% of total) were classified into 25 functional KOG classifications. **b** The GO functional classification of unigenes. A total of 6053 machine annotated unigenes were grouped into 40 functional group categories using GO assignment. **c**, **d** and **e** The GO functional classification of DEGs. These DEGs were classified into three main categories including cellular component, biological process and molecular function

As shown in Fig. 2a, 5172 unigenes (48.11% of total) were classified into 25 functional KOG classifications, based on sequence similarity. The predominant term was “general function prediction only,” for which 1648 unigenes (31.86%) were qualified. “Posttranslational modification, protein turnover, chaperone” (1193 unigenes), “signal transduction mechanism” (1048 unigenes), and “RNA processing and modification” (721 unigenes) were other major categories selected, and only eight, 22, 53, and 54 unigenes matched the terms “cell motility,” “extracellular structure,” “defense mechanism,” and “nuclear structure,” respectively.

A total of 6053 machine annotated unigenes were grouped into 40 functional group categories using GO assignment (Fig. 2b). Among these categories, 15 are involved in “biological process,” 11 in “molecular function,” and 14 in “cellular component.” “Metabolic process” (3422 unigenes) and “cellular process” (3121 unigenes) are dominant among these; “detoxification (four unigenes) and growth” (21 unigenes) are the scarcest in the “biological process” category. Within the “molecular function” category, a high percentage of genes are associated with “catalytic activity” (3538 unigenes) and “binding” (3272 unigenes). Minimal “molecular function” GO assignments included “signal transducer activity” (21 unigenes) and “electron carrier activity” (22 unigenes). Within the “cellular components category,” “cell” (2374 unigenes) and “cell part” (2374 unigenes) are predominant; “nucleoid” (six unigenes) and “supramolecular fiber” (six unigenes) had the fewest matches.

A total of 2292 unigenes were annotated to 114 pathways in this study; the pathways of all our unigenes are shown in Additional file 4. “Metabolic pathway” represented the largest group (1916 unigenes), with most being involved in the “biosynthesis of amino acids” (138 unigenes), “carbon metabolism” (118 unigenes), “purine metabolism” (117 unigenes), and “oxidative phosphorylation” (113 unigenes). Secondary pathways included “genetic information processing” (1103 unigenes), which included “ribosome” (151 unigenes), “RNA transport” (99 unigenes), “spliceosome” (94 unigenes), “protein processing in endoplasmic reticulum” (93 unigenes), and “endocytosis” (92 unigenes). These probable pathways provide a valuable resource for investigating specific metabolic processes and gene functions in *T. mentagrophytes.*

### Overview of differentially expressed genes

FDR and log2FC were both used to filter our DEGs, with the filter conditions of FDR < 0.05 and |log2FC| > 1. We compared Norm (as a control) to Zn400 and Zn1000. Results showed 2314 and 2127 genes are significantly up- and down-regulated, respectively, in Zn400. In Zn1000, 1395 and 1446 genes displayed significant up-regulation and down-regulation, respectively. A comparison was also performed using Zn1000 as the control, against Zn400. This result showed significant up- and down-regulation: 2268 and 2040 genes were regulated in Zn400 as compared with Zn1000, respectively. All DEGs are shown in Additional file 5, and a group diffuse analysis ‘volcano plot’ is shown in Additional file 6.

The Zn400 DEGs were subjected to GO-term analysis (Fig. 2c); these DEGs partitioned into three major categories: “cellular component” (1165), “biological process” (1880), and “molecular function” (2024). These major categories sorted into several subcategories (based on Pvalue <0.05 and Qvalue <0.05): “electron carrier activity” within the “molecular function” category, and “generation of precursor metabolites and energy,” “oxidation-reduction process,” “electron transport chain,” “purine nucleoside biosynthetic process,” “purine ribonucleoside biosynthetic process,” “multi-organism process,” “monovalent inorganic cation transport,” and “proton transport” within the “biological process” category.

The Zn1000 DEGs were also subjected to GO-term analysis (Fig. 2d), These DEGs mainly tagged “preribosome,” “oxidation-reduction process,” “oxidoreductase activity,” and “acting on other nitrogenous compounds as donor” subcategories (based on Pvalue <0.05 and Qvalue <0.05). DEGs in the comparison of Zn1000 with Zn400 mainly tagged “ion binding” and “cation binding” subcategories (based on Pvalue <0.05 and Qvalue <0.05) (Fig. 2e).

All Zn400 DEGs were subjected to pathway enrichment analysis. Up to 19.34% of the DEGs could be annotated, and 112 pathways were obtained (Additional file 5). Many pathways were significantly enriched (Pvalue <0.05, Qvalue <0.05) including oxidative phosphorylation; valine, leucine, and isoleucine biosynthesis; biosynthesis of amino acids; carbon metabolism; pantothenate and CoA biosynthesis; and the citrate cycle (TCA cycle). In Zn1000, 19.39% of the DEGs could be annotated, and 109 pathways were obtained. Oxidative phosphorylation is significantly enriched (Pvalue <0.05, Qvalue <0.05) (Additional file 7).

### Identifying *T. mentagrophytes* zinc-uptake-related genes

**Fig. 3:**
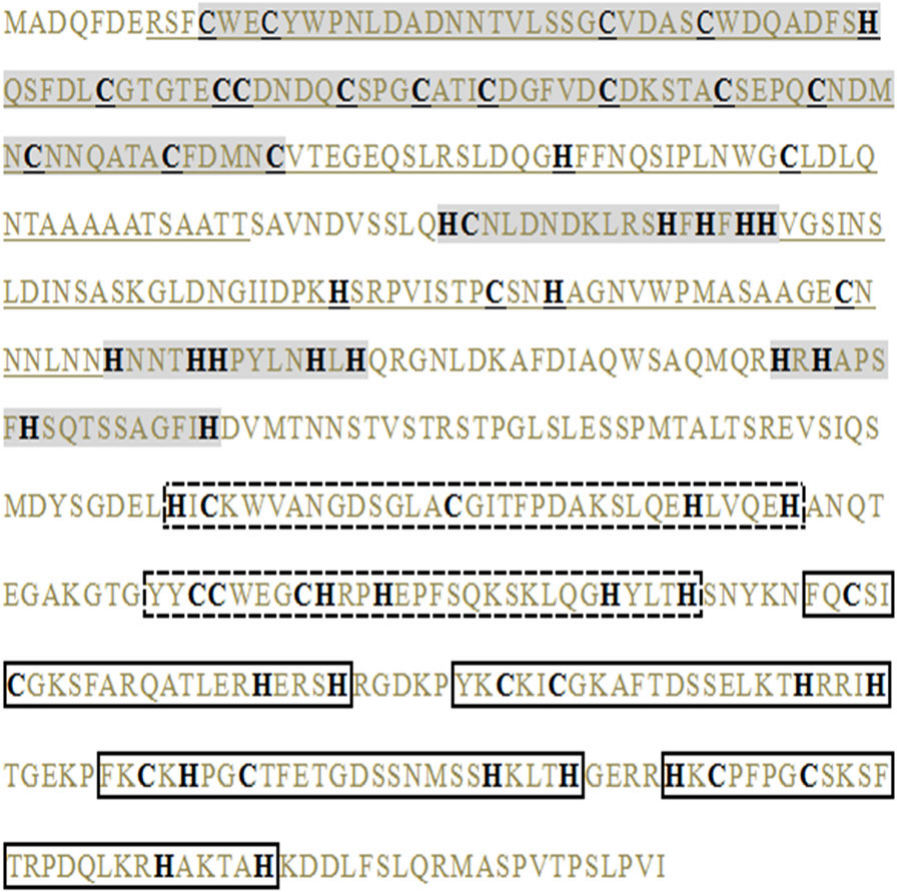
The predicted amino acid sequence encoded by the unigene0008014. Putative zinc-binding domains (ZBD) and activating domains (AD) are shaded and underlined respectively. Zinc fingers are squared including typical fingers (solid line square) and non-typical fingers (dotted line square). Cysteine and histidine residues are in bold

Zinc uptake system expression in the model fungus *S. cerevisiae* is primarily regulated at the transcriptional level by the C_2_H_2_-type zinc finger transcription factor *Zap1* [5]. Subsequent studies have shown other fungi, for example *A. fumigatus*, can secrete functionally similar zinc finger transcriptional factor *ZafA* proteins [18]. A BLASTx [19] sequencing similarity search was performed using the *A. fumigatus ZafA* and *S. cerevisiae Zap1* protein sequences against our unigenes. We found a total of 100 unigenes similar to *A. fumigatus ZafA*, and 53 unigenes to *S. cerevisiae Zap 1*, respectively. A total of 36 of these unigenes have similarities with both *ZafA* and *Zap1*, but only 28 have a zinc finger structure (Additional file 8), according to functional annotation matches. Of these 28 unigenes with annotated zinc fingers, the sequence with the highest similarity to both *ZafA* and *Zap1* was Unigene0008014, which was a DEG in the transcriptome sequencing comparison of Norm versus Zn400, but was not a DEG in Norm versus Zn1000. Regardless, the predicted protein encoded by this gene is the most similar to the ZafA protein of *A. fumigatus* [18], unambiguously fits the zinc finger consensus [20], and has putative zinc-binding domains [21] (Fig. 3). Furthermore, Unigene0008014 qualified for “zinc-responsiveness transcriptional activator” (*Trichophyton equinum* CBS 127.97) and “zinc-responsive transcriptional regulator Zap1” (*S. cerevisiae* strain ATCC 204508/S288c) Nr and Swiss-Prot annotations in our analyses, respectively. We hypothesize that Unigene0008014 plays an important role in regulating zinc ion uptake in *T. mentagrophytes,* and be named a zinc-responsive activating factor. The zinc-responsive activating factor genomic sequence can be amplified using three different pairs of primers in *T. mentagrophytes* (primers shown in Additional file 9). The nucleotide sequence of our putative zinc-responsive activating factor has been submitted to NCBI GenBank under the accession KY420911.

Four of our unigenes, Unigene0002709, Unigene0002593, Unigene0004712, and Unigene0005637, are likely zinc transporters. Nr annotations for these transcripts, “zinc/iron transporter” (*Trichophyton tonsurans CBS* 112818), “plasma membrane zinc ion transporter” (*Trichophyton equinum CBS* 127.97), “membrane zinc transporter” (*Trichophyton tonsurans CBS* 112818), and “ZIP family zinc transporter” (*Trichophyton tonsurans CBS* 112818), respectively, support this hypothesis. Moreover, Swiss-Prot annotations, “zinc-regulated transporter” (*S. cerevisiae* strain ATCC 204508/S288c), “RNA polymerase II transcription factor B subunit” (*Candida glabrata* strain ATCC 2001/CBS 138/JCM 3761/NBRC 0622/NRRL Y-65), “zinc-regulated transporter” (*S. cerevisiae* strain ATCC 204508/S288c), “zinc-regulated transporter” (*S. cerevisiae* strain ATCC 204508/S288c), respectively, corroborate the assertion. Additionally, these four unigenes have high BLASTx [19] similarity with *ZrfA*, *ZrfB*, *ZrfC*, and *Aspf2* of *A. fumigatus* based on E-value.

**Fig. 4:**
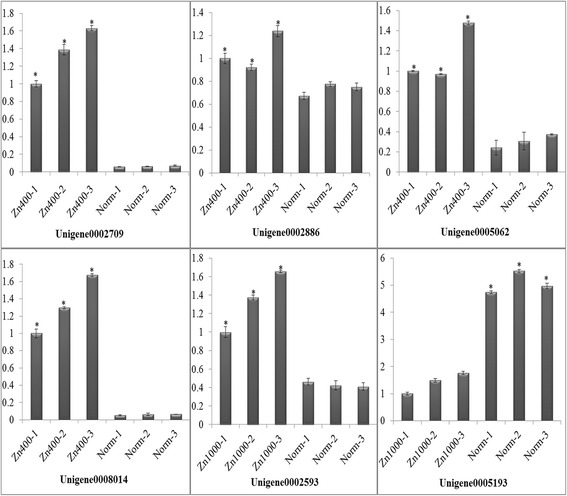
The result of qRT-PCR in 6 unigenes. Unigene0002709, Unigene0008014, Unigene0002886, and Unigene0005062 exhibited differential expression levels, in Zn400. Unigene0002593 and Unigene0005193 exhibited differential expression levels in Zn1000

To validate changes in gene expression patterns we used qRT-PCR against six unigenes: Unigene0008014, Unigene0002709, Unigene0002593, Unigene0002886, Unigene0005062, and Unigene0005193. Unigene0002709, Unigene0008014, Unigene0002886, and Unigene0005062 exhibited differential expression levels, identical to those obtained by sequencing in Zn400. Unigene0002593 and Unigene0005193 exhibited differential expression levels, identical to those obtained by sequencing in Zn1000 (Fig. 4). A statistical analysis was performed on the qRT-PCR differential expression analyses and is shown in Additional file 10.

Data from this study sufficiently support our investigation into genes related to zinc ion uptake regulation in *T. mentagrophytes*.

### The change of phenotype in *ZafA* gene mutant strain

**Fig. 5:**
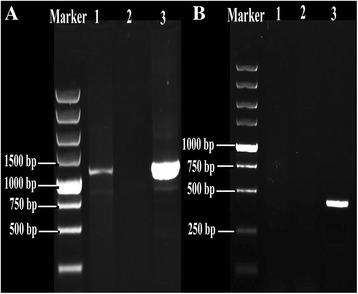
PCR analysis of transformants. **a** Amplification of *hph* (1200 bp) using the primers *hph*-F and *hph*-*R. Lane* 1, DNA sample from the *T. mentagrophytes ZafA* gene mutant strain; lane 2, DNA sample from the wild-type *T. mentagrophytes* strain 28,185, lane 3, the transformation vector *pDHt/ZafA:: hph*. **b** Amplification of the *ZafA* gene fragment disrupted (400 bp) using the primers *ZafA*-F and *ZafA*-*R. Lane* 1, DNA sample from the *T. mentagrophytes ZafA* gene mutant strain; lane 2, the transformation vector *pDHt/ZafA:: hph*; lane 3, DNA sample from the wild-type *T. mentagrophytes* strain 28,185

**Fig. 6:**
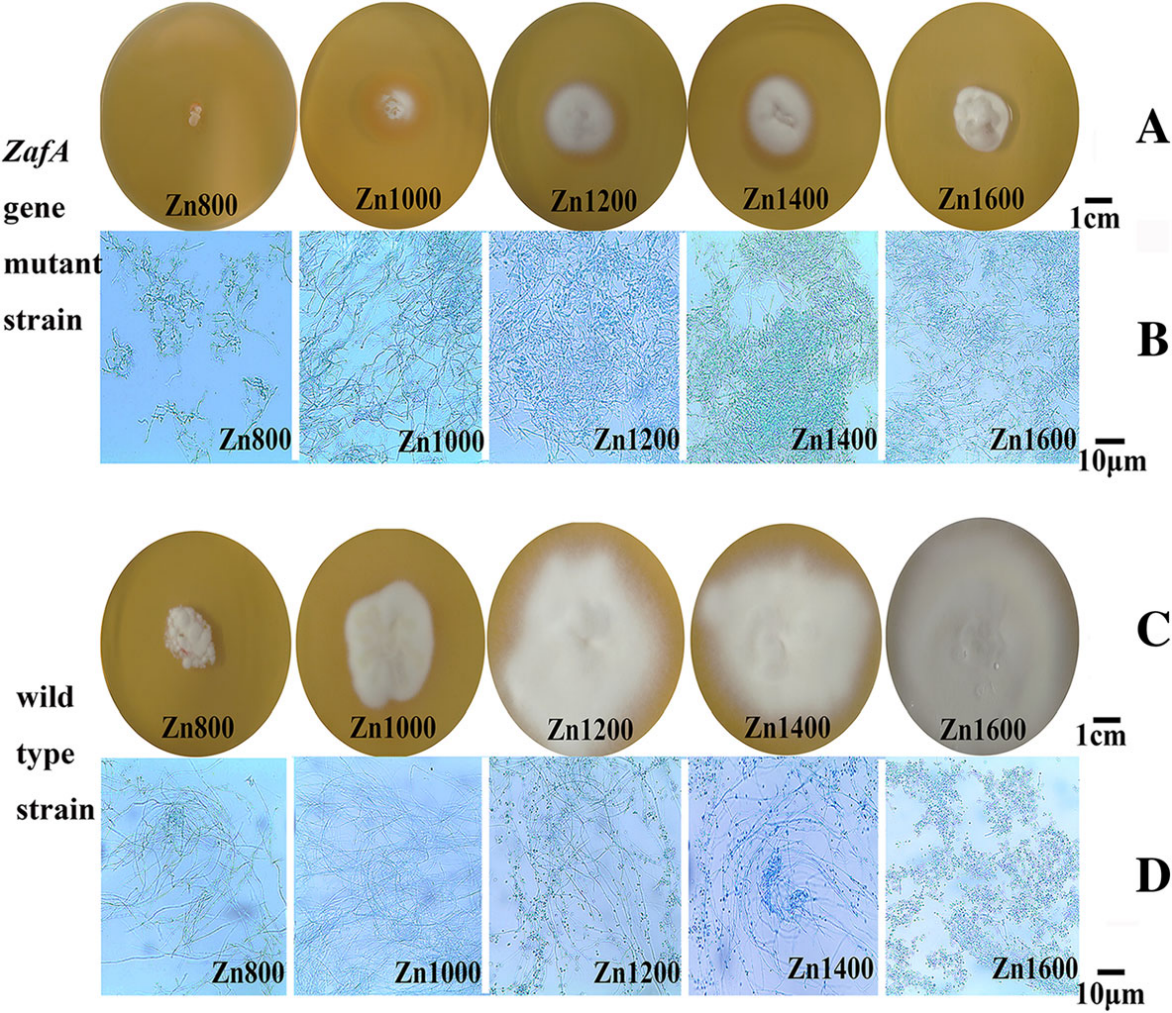
The growth situation of *T. mentagrophytes* wild-type strain and *ZafA* gene mutant strain on SDA-Zn medium with 800, 1000, 1200, 1400 and 1600 μM zinc sulfate. **a** The growth situation of *ZafA* gene mutant strain, the growth of *T. mentagrophyte* is inhibited, especially in Zn800 and Zn1000. **b** Under the microscope, there is good quantity of mycelium in *ZafA* gene mutant strain, but the number of conidia is reduced. **c** The wild-type strain grows normally, and with increasing of zinc ion concentration, the growth rate is accelerated. **d** Under the microscope, there are good quantity of mycelium and a mass of conidia in wild-type strain

To observe the changes of phenotype and the growth ability after *ZafA* gene deleted in *T. mentagrophytes*. The *ZafA* gene was knocked by ATMT transformation in *T. mentagrophytes*. The fragment of *hph* gene could be amplified, and the fragment of *ZafA* gene could not be amplified in *ZafA* gene mutant strain (Fig. 5), this means that the *ZafA* gene is completely removed. The *T. mentagrophytes* wild-type strain and *ZafA* gene mutant strain were maintained at 28 °C on SDA-Zn medium with 800, 1000, 1200, 1400 and 1600 μM zinc sulfate for 16 days. The changes of phenotypic and growth ability are shown in Fig. 6. The wild-type strain can begin growing normally in third day, and with increasing of zinc ion concentration, the growth rate is accelerated. But the *ZafA* gene mutant strain can begin growing in eighth day, and the growth rate and state are much lower than the wild-type strain in same situation. Under the microscope, there was no significant difference in the quality and quantity of mycelium between wild-type strain and *ZafA* gene mutant strain, but the number of conidia of *ZafA* gene mutant strain was obviously less than wild-type strain in same culture situation. The result showed that the deletion of *Zafa* gene can negatively affect the growth and the number of conidia of *T. mentagrophytes*.

## Discussion

### Illumina sequencing and sequence annotation in *T. mentagrophytes*

The infection of skin, nail, hair, and fur caused by dermatophytes is called dermatophytosis. As early as 1839, scientists confirmed that dermatophytes can cause human disease. Furthermore, at least 10–20% of the world’s population may be infected with dermatophytes [22]. Dermatophytes comprise three genera: *Microsporum*, *Trichophyton*, and *Epidermophyton*. An important member of *Trichophyton, T. mentagrophytes* can cause severe skin infections in humans and other animals, and has a wide distribution around the world [23]. Therefore, *T. mentagrophytes* warrants investigation. Our study aimed to generate a large amount of cDNA sequence data to facilitate more detailed transcriptomics studies in *T. mentagrophytes*, and, in particular, to identify genes related to the regulation of zinc ion uptake in that organism. RNA-seq is a powerful tool that can provide a global overview of genes expression at the transcriptome level [24]; however, it has not been extensively applied to fungi. We believe RNA-seq will prove to be a powerful method for fungi study. The availability of our *T. mentagrophytes* transcriptome data will meet the initial information needs for functional studies of this species and its relatives.

We chose to sequence the transcriptomes of Norm, Zn400, and Zn1000, based on our growth experiment results. Zn1000 and Norm samples grew similarly in our trials; there was a significant difference between Zn400 and Norm, microconidia and mycelium morphology are also significantly different between the two. The RNA-seq method was then performed on these samples using Illumina sequencing, which generated a total of 10,751 unigenes, of which more than 89% were annotated by our analyses. These data will provide a valuable resource for the study of *T. mentagrophytes*.

### Zn-deficiency induced changes in *T. mentagrophytes* growth and gene expression

Fungi rely on zinc for growth; the zinc ions serve as a cofactor in numerous proteins, including important transcription factors [25]. Zinc chelation can reduce fungal growth in both rich and defined media [26]. Zinc chelation occurs during infection, and is an important strategy evolved in immune cells to hamper pathogen growth [27]. Zn1000 cells grew with no obvious morphological differences compared with Norm in our study. However, with sequential zinc ion concentration decreases in Zn200, Zn400, and Zn600, microconidia and mycelium morphology showed *T. mentagrophytes* growth to be increasingly restricted by zinc deficiency. Furthermore, when *T. mentagrophytes* growth was inhibited in Zn200, Zn400, and Zn600 zinc deficient samples, which were then inoculated into normal medium or Zn1000 zinc deficiency medium, *T. mentagrophytes* cells restore to normal growth patterns due to the increase in zinc concentration. This suggests that the growth inhibition we observed in *T. mentagrophytes* is primarily caused by a lack of zinc ions, rather than other metal ions, and it reinforces the hypothesis that *T. mentagrophytes* natural growth requires zinc ions. Zinc ion acquisition is, therefore, crucial for the growth and development of *T. Mentagrophytes.*

The *S. cerevisiae* yeast cell employs several different strategies to cope with stress caused by zinc deprivation [28]. Zinc deprivation induced by TPEN also induces a variety of changes in the gene expression in *C. gattii* cells [9]. Studies have shown that the growth and gene expression, in particular, high-levels of zinc transporter system expression, within a variety of fungi, including *A. fumigatus* [18], *C. albicans* [10], and *C. gattii* [9], are affected by zinc deficiency. Similarly, our RNA-seq data differential expression analyses revealed 4441 DEGs (2314 up-regulated and 2127 down-regulated) in Zn400, versus 2841 DEGs (1395 up-regulated and 1446 down-regulated) in Zn1000. Because of the higher zinc ion concentration, Zn1000 has fewer DEGs than Zn400. This further indicates that the change of gene expression in *T. mentagrophytes* is predominantly caused by a lack of zinc ions, versus other metal ions. Zinc deficiency definitely affects gene expression in *T. mentagrophytes*. We speculate that *T. mentagrophytes* regulates the expression of many genes under conditions of zinc deficiency.

A genome-wide, functional analysis revealed that almost 400 different gene products are necessary for proper growth in zinc-limiting conditions, using a *S. cerevisiae* mutant library [29]. Of these proteins, most are associated with oxidative stress response, endoplasmic reticulum function, peroxisome biogenesis, or zinc uptake. Furthermore, as revealed by transcriptomic and functional analyzes, also in *S. cerevisiae*, low zinc conditions lead to alterations in lipid synthesis, sulfate metabolism, and oxidative stress tolerance [30, 31]. Our KEGG and GO DEG analyses showed that *T. mentagrophytes* has the same response as *S. cerevisiae* under zinc deficient environments. Most of our DEGs partitioned into either oxidation-reduction process, electron carrier activity, purine ribonucleoside biosynthetic process, monovalent inorganic cation transport, or proton transport, in the GO analysis. Furthermore, oxidative phosphorylation; valine, leucine and isoleucine biosynthesis; biosynthesis of amino acids; carbon metabolism; and the citrate cycle (TCA cycle) were significantly enriched in our pathway enrichment analysis. These results suggest that zinc deprivation can affect *T. mentagrophytes* development, growth, and gene expression. We speculate *T. mentagrophytes* can change particular metabolic pathways to resist zinc deficiency. However, this stressed cellular state cannot last forever, thus *T. Mentagrophytes* growth and development are eventually negatively impacted, to the point of cellular death under extended zinc deficiency.

### Zinc-uptake-related genes in *T. mentagrophytes*

Fungi cells must acquire zinc ions for proper life cycle development, even as saprophytes, or during infective processes [32]. Zinc transport mechanisms were initially characterized in fungi with *S. cerevisiae Zap1, Zrt1*, and *Zrt2* genes [33]. *Zap1* can activate the transcription of *Zrt1* and *Zrt2* by binding to the zinc-response element in its promoter region; binding affinity is controlled by zinc level [5]. Zinc uptake and homeostasis is also important in the physiology and virulence of *A. fumigatus, C. albicans*, and *C. gattii.* The main function of the *A. fumigatus ZafA* protein is to regulate zinc uptake; it is a requisite for the growth of *A. fumigatus* in zinc-limited conditions [18]. Furthermore, *ZafA* mutants do not survive and have no pathogenicity in the mice lung, supporting the essential role of *A. fumigatus ZafA* in growth and virulence [18]. In addition, *ZafA* can induce *Zrf* and *Aspf2* gene expression under zinc deficient conditions, and its expression is also influenced by zinc concentration [8, 34]. *Csr1/Zap1* is considered a homolog of *S. cerevisiae Zap1* in *C. albicans*. Mutants lacking *Csr1/Zap1* alleles show growth deficiencies under zinc deficient conditions, and cannot form germ tubes or hyphae, demonstrating that *Csr1/Zap1* contributes to zinc uptake and homeostasis, as well as morphological transitions, in *C. albicans* [10]. Another pathogenic *Candida* species, *C. dubliniensis* also possesses the *S. cerevisiae Zap1* homolog *Csr1*. Mutants show growth defects under zinc-limited conditions. However, unlike *C. albicans*, these *Csr1* mutants are able to form germ tubes and undergo morphological transition, although the mutants do exhibit reduced virulence [35]. Similar to other fungi, the *S. cerevisiae* zinc finger transcription factor *Zap1* homolog was identified in *C. gattii*. The *Zap1* homolog mutant showed impaired growth under zinc-limited conditions compared with wild-type. Furthermore, the *Zap1* mutant displayed attenuated virulence in a murine cryptococcosis model. This suggests that *Zap1* plays critical roles in zinc uptake and virulence in *C. gattii* [9].

In the present study we found 28 unigenes with similarity to both *ZafA* of *A. fumigatus* and *Zap1* of *S. cerevisiae* according to functional annotation matches and BLASTx. We hypothesize that Unigene0008014 is functionally similar to both *ZafA* of *A. fumigatus* and *Zap1* of *S. cerevisiae*, based on sequence similarity, functional annotation, and the predicted protein product. The significant up-regulation of Unigene0008014 expression levels, in particular for Zn400, as detected by qRT-PCR, with such a dramatic difference in our transcriptome sequencing comparison for Zn400, support this hypothesis. We also knocked *ZafA* gene by ATMT transformation in *T. mentagrophytes*. By observing the changes of phenotype and the growth ability after *ZafA* gene deleted in *T. mentagrophytes.* We found that *ZafA* gene is very important for the growth and the generation of conidia in *T. mentagrophytes*. We think that Unigene0008014 can regulate zinc uptake at the transcriptional level as a C_2_H_2_-type zinc finger transcription factor in *T. mentagrophytes,* and name it a zinc-responsive activating factor.

The other 27 *ZafA*/*Zap1* putative homologs do not appear to be zinc-responsive activating factors, but do contain zinc finger structures*,* and many show significant expression changes under zinc deficiency, suggesting that these 27 unigenes have important metabolic functions. Therefore, we consider these 27 unigenes to possibly function as a regulator of zinc uptake; however, this speculation requires further research.

We also found four *T. mentagrophytes* unigenes, Unigene0002709, Unigene0002593, Unigene0004712, and Unigene0005637, that may be zinc transporters. These four sequences are similar to the zinc transporters of *A. fumigatus* and *S. cerevisiae* based on E-value, and our gene function annotation analyses show these four unigenes to be zinc transporters. Furthermore, Unigene0002709 and Unigene0002593 qRT-PCR results showed significant up-regulation. Unigene0005637 was not a DEG, and Unigene0004712 was significantly down-regulated. We think that these results may be due to the pH value of the medium partly inhibiting Unigene0005637 and Unigene0004712 expression levels. A similar effect has been observed in *A. fumigatus* [7]. Indeed, different pH values can affect the expression of various zinc transporters [8]. Thus, we think that the zinc transporter system in *T. mentagrophytes* comprises five unigenes: Unigene0008014, Unigene0002709, Unigene0002593, Unigene0004712, and Unigene0005637. However, the possibility that these five unigenes are just a part of a larger zinc transporter system exists, and requires further analysis.

## Conclusion

In this study we report the first large transcriptome study carried out in *T. mentagrophytes* where we have compared physiological and transcriptional responses to zinc deficiency. A total of 10,751 unigenes were obtained and more than 89% of them were annotated. This provided more adequate resources to study *T. mentagrophytes*. Evidence from physiological observations, transcriptome and qRT-PCR analysis indicated that zinc deficiency could induce arrested development, and numerous genes expression changes in *T. mentagrophytes*. Importantly, we found the zinc-responsive activating factor (unigene0008014) and we speculated that 4 unigenes (unigene0002709, unigene0002593, unigene0004712, unigene0005637) are zinc transporters. The expression of these 4 zinc transporter genes is potentially regulated by the zinc-responsive activating factor. And we knocked *ZafA* gene in *T. mentagrophytes*, the result showed that *ZafA* gene is very important for the growth and the generation of conidia in *T. mentagrophytes*.

## Additional files

Additional file 1: The primers of qRT-PCR. (DOCX 15 kb)

Additional file 2: The saturation curves of RNA-seq. (TIFF 2098 kb)

Additional file 3: The Nr, Swiss-Prot, KEGG and KOG annotation of unigenes. (XLSX 3000 kb)

Additional file 4: The pathways of unigenes. (DOCX 29 kb)

Additional file 5: All DEGs. (XLSX 1503 kb)

Additional file 6: Group diffuse analysis ‘volcano plot’. (TIFF 1807 kb)

Additional file 7: The pathways of DEGS. (XLSX 27 kb)

Additional file 8: The unigenes that have zinc finger structure. (DOCX 16 kb)

Additional file 9: Three pairs of primers that can amplify zinc-responsive. (DOCX 15 kb)

Additional file 10: The qRT-PCR differential expression analyses. (DOCX 17 kb)

## Abbreviations

*A. fumigatus*: *Aspergillus fumigatus*
ATMT: Agrobacterium tumefaciens-mediated transformation
*C. albicans*: *Candida albicans*
*C. gattii*: *Cryptococcus gattii*
COG: Clusters of orthologous groups of protein
DEGs: Differentially Expressed Genes
EDTA: Ethylene Diamine Tetraacetic Acid
GO: Gene ontology
*hph*: Hygromycin B resistance gene
KEGG: Kyoto encyclopedia of genes and genomes
Nr: Non-redundant protein database
qRT-PCR: Quantitative real time PCR
*S. cerevisiae*: *Saccharomyces cerevisiae*
SDA: Sabouraud dextrose medium
SDA-Zn: Zinc deficient SDA
Swiss-Prot: Annotated protein sequence database
*T. mentagrophytes*: *Trichophyton mentagrophytes*
*ZafA*: Zinc-responsive activating factor
