# Supplementary material for: Transcriptome sequencing and analysis of zinc-uptake-related genes in Trichophyton mentagrophytes
Source: BMC Genomics. 2017 Nov 21;18:888. doi: 10.1186/s12864-017-4284-3 (PMC5697147; doi:10.1186/s12864-017-4284-3)
Supplement: Supplementary file 1 — The primers of qRT-PCR. (DOCX 15 kb) [file 12864_2017_4284_MOESM1_ESM.docx]

| QPCR-0008014-F | CGCACATTTCCCGAACTACAACC |
| --- | --- |
| QPCR-0008014-R | GCCAATAGCACTCCCAGCAGAAT |
| QPCR-0002709-F | GCCTTCCTTTCGTTCTCGTA |
| QPCR-0002709-R | AACTCCGCCTCTATTCGTCA |
| QPCR-0002593-F | AAAGTGGCGGTGTAGATGGT |
| QPCR-0002593-R | AACGAGGATACGAAGAAGATGA |
| QPCR-0002886-F | AGCAGCGTACTGGCGGATTT |
| QPCR-0002886-R | CTCAACGACCACGACCAACG |
| QPCR-0005062-F | CCGTGTCCGCCTCATCAAAC |
| QPCR-0005062-R | CGCTACCAGCACCAGCAACC |
| QPCR-0005193-F | GAACGAGCAGGAAGACGAGA |
| QPCR-0005193-F | CCGCCAGAATAACAGAGGAA |
| 18S-F | AGGCGCGCAAATTACCCAATCC |
| 18s-R | GCCCTCCAATTGTTCCTCGTTAAG |

The primers of qRT-PCR
