# Supplementary figures and images for: Transcriptome sequencing and analysis of zinc-uptake-related genes in Trichophyton mentagrophytes
Source: BMC Genomics. 2017 Nov 21;18:888. doi: 10.1186/s12864-017-4284-3 (PMC5697147; doi:10.1186/s12864-017-4284-3)

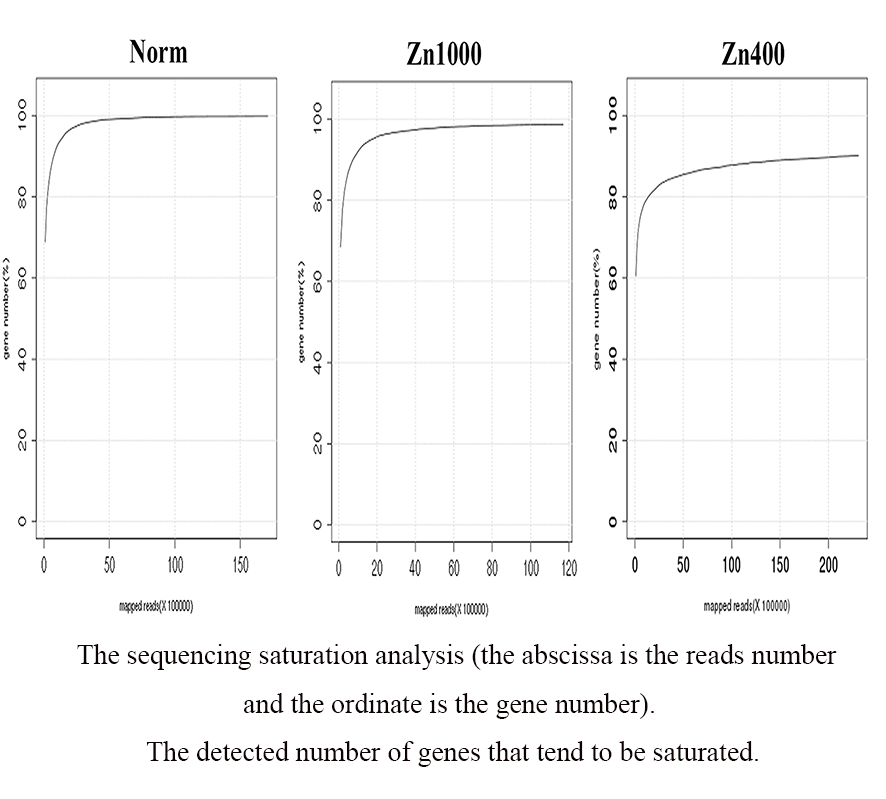

Supplement: Supplementary file 2 — The saturation curves of RNA-seq. (TIFF 2098 kb) [file 12864_2017_4284_MOESM2_ESM.tif]

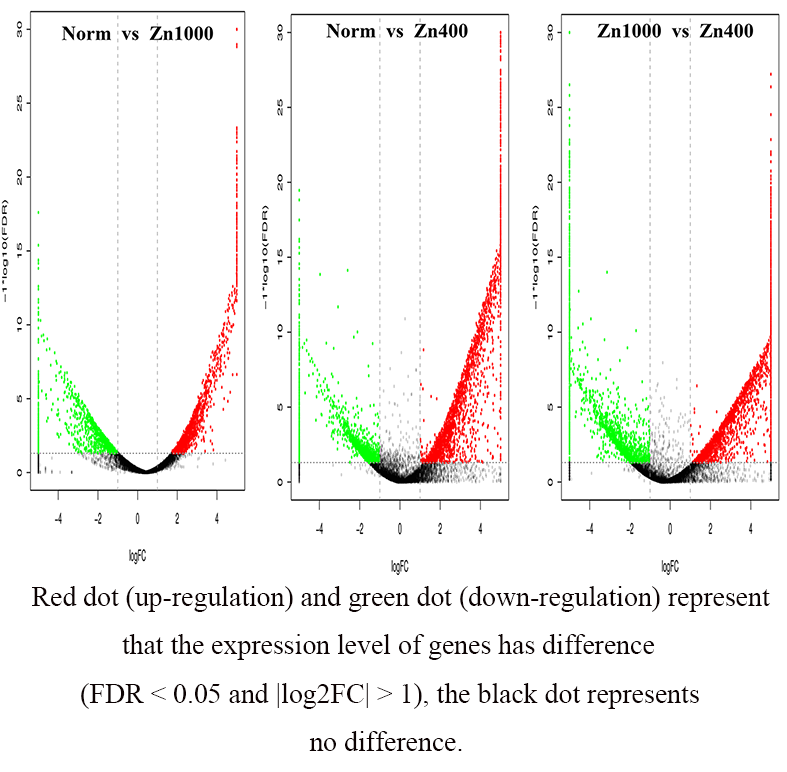

Supplement: Supplementary file 6 — Group diffuse analysis ‘volcano plot’. (TIFF 1807 kb) [file 12864_2017_4284_MOESM6_ESM.tif]
