# Supplementary material for: Transcriptome sequencing and analysis of zinc-uptake-related genes in Trichophyton mentagrophytes
Source: BMC Genomics. 2017 Nov 21;18:888. doi: 10.1186/s12864-017-4284-3 (PMC5697147; doi:10.1186/s12864-017-4284-3)
Supplement: Supplementary file 4 — The pathways of unigenes. (DOCX 29 kb) [file 12864_2017_4284_MOESM4_ESM.docx]

The pathways of all unigenes

|  | **Pathway** | **All genes with pathway annotation** | **Pathway ID** |
| --- | --- | --- | --- |
| 1 | [Ribosome](file:///G:\WEN\%E8%BD%AC%E5%BD%95%E7%BB%84%E6%B5%8B%E5%BA%8F\%E3%80%90%E6%89%B9%E9%87%8F%E4%B8%8B%E8%BD%BD%E3%80%91GDR1624-%E8%A5%BF%E5%8C%97%E5%86%9C%E6%9E%97%E7%A7%91%E6%8A%80%E5%A4%A7%E5%AD%A6%E5%8A%A8%E7%89%A9%E5%8C%BB%E5%AD%A6%E9%99%A24%E4%BE%8B%E7%9C%9F%E8%8F%8C%E8%BD%AC%E5%BD%95%E7%BB%84%E6%B5%8B%E5%BA%8F%E9%A1%B9%E7%9B%AE-%E5%89%94%E9%99%A4T2%E6%A0%B7%E5%93%81-Denovo_%E5%AE%8C%E6%95%B4%E7%89%88%E7%BB%93%E9%A2%98%E6%8A%A5%E5%91%8A%E7%AD%89\GDR1624-%E8%A5%BF%E5%8C%97%E5%86%9C%E6%9E%97%E7%A7%91%E6%8A%80%E5%A4%A7%E5%AD%A6%E5%8A%A8%E7%89%A9%E5%8C%BB%E5%AD%A6%E9%99%A24%E4%BE%8B%E7%9C%9F%E8%8F%8C%E8%BD%AC%E5%BD%95%E7%BB%84%E6%B5%8B%E5%BA%8F%E9%A1%B9%E7%9B%AE-Denovo-RNAseq_result\4_basic_annotation\KEGG\Trichophyton_mentagrophytes-Unigene.fa.htm#gene1) | 151 (6.59%) | ko03010 |
| 2 | [Biosynthesis of amino acids](file:///G:\WEN\%E8%BD%AC%E5%BD%95%E7%BB%84%E6%B5%8B%E5%BA%8F\%E3%80%90%E6%89%B9%E9%87%8F%E4%B8%8B%E8%BD%BD%E3%80%91GDR1624-%E8%A5%BF%E5%8C%97%E5%86%9C%E6%9E%97%E7%A7%91%E6%8A%80%E5%A4%A7%E5%AD%A6%E5%8A%A8%E7%89%A9%E5%8C%BB%E5%AD%A6%E9%99%A24%E4%BE%8B%E7%9C%9F%E8%8F%8C%E8%BD%AC%E5%BD%95%E7%BB%84%E6%B5%8B%E5%BA%8F%E9%A1%B9%E7%9B%AE-%E5%89%94%E9%99%A4T2%E6%A0%B7%E5%93%81-Denovo_%E5%AE%8C%E6%95%B4%E7%89%88%E7%BB%93%E9%A2%98%E6%8A%A5%E5%91%8A%E7%AD%89\GDR1624-%E8%A5%BF%E5%8C%97%E5%86%9C%E6%9E%97%E7%A7%91%E6%8A%80%E5%A4%A7%E5%AD%A6%E5%8A%A8%E7%89%A9%E5%8C%BB%E5%AD%A6%E9%99%A24%E4%BE%8B%E7%9C%9F%E8%8F%8C%E8%BD%AC%E5%BD%95%E7%BB%84%E6%B5%8B%E5%BA%8F%E9%A1%B9%E7%9B%AE-Denovo-RNAseq_result\4_basic_annotation\KEGG\Trichophyton_mentagrophytes-Unigene.fa.htm#gene2) | 138 (6.02%) | ko01230 |
| 3 | [Carbon metabolism](file:///G:\WEN\%E8%BD%AC%E5%BD%95%E7%BB%84%E6%B5%8B%E5%BA%8F\%E3%80%90%E6%89%B9%E9%87%8F%E4%B8%8B%E8%BD%BD%E3%80%91GDR1624-%E8%A5%BF%E5%8C%97%E5%86%9C%E6%9E%97%E7%A7%91%E6%8A%80%E5%A4%A7%E5%AD%A6%E5%8A%A8%E7%89%A9%E5%8C%BB%E5%AD%A6%E9%99%A24%E4%BE%8B%E7%9C%9F%E8%8F%8C%E8%BD%AC%E5%BD%95%E7%BB%84%E6%B5%8B%E5%BA%8F%E9%A1%B9%E7%9B%AE-%E5%89%94%E9%99%A4T2%E6%A0%B7%E5%93%81-Denovo_%E5%AE%8C%E6%95%B4%E7%89%88%E7%BB%93%E9%A2%98%E6%8A%A5%E5%91%8A%E7%AD%89\GDR1624-%E8%A5%BF%E5%8C%97%E5%86%9C%E6%9E%97%E7%A7%91%E6%8A%80%E5%A4%A7%E5%AD%A6%E5%8A%A8%E7%89%A9%E5%8C%BB%E5%AD%A6%E9%99%A24%E4%BE%8B%E7%9C%9F%E8%8F%8C%E8%BD%AC%E5%BD%95%E7%BB%84%E6%B5%8B%E5%BA%8F%E9%A1%B9%E7%9B%AE-Denovo-RNAseq_result\4_basic_annotation\KEGG\Trichophyton_mentagrophytes-Unigene.fa.htm#gene3) | 118 (5.15%) | ko01200 |
| 4 | [Purine metabolism](file:///G:\WEN\%E8%BD%AC%E5%BD%95%E7%BB%84%E6%B5%8B%E5%BA%8F\%E3%80%90%E6%89%B9%E9%87%8F%E4%B8%8B%E8%BD%BD%E3%80%91GDR1624-%E8%A5%BF%E5%8C%97%E5%86%9C%E6%9E%97%E7%A7%91%E6%8A%80%E5%A4%A7%E5%AD%A6%E5%8A%A8%E7%89%A9%E5%8C%BB%E5%AD%A6%E9%99%A24%E4%BE%8B%E7%9C%9F%E8%8F%8C%E8%BD%AC%E5%BD%95%E7%BB%84%E6%B5%8B%E5%BA%8F%E9%A1%B9%E7%9B%AE-%E5%89%94%E9%99%A4T2%E6%A0%B7%E5%93%81-Denovo_%E5%AE%8C%E6%95%B4%E7%89%88%E7%BB%93%E9%A2%98%E6%8A%A5%E5%91%8A%E7%AD%89\GDR1624-%E8%A5%BF%E5%8C%97%E5%86%9C%E6%9E%97%E7%A7%91%E6%8A%80%E5%A4%A7%E5%AD%A6%E5%8A%A8%E7%89%A9%E5%8C%BB%E5%AD%A6%E9%99%A24%E4%BE%8B%E7%9C%9F%E8%8F%8C%E8%BD%AC%E5%BD%95%E7%BB%84%E6%B5%8B%E5%BA%8F%E9%A1%B9%E7%9B%AE-Denovo-RNAseq_result\4_basic_annotation\KEGG\Trichophyton_mentagrophytes-Unigene.fa.htm#gene4) | 117 (5.1%) | ko00230 |
| 5 | [Oxidative phosphorylation](file:///G:\WEN\%E8%BD%AC%E5%BD%95%E7%BB%84%E6%B5%8B%E5%BA%8F\%E3%80%90%E6%89%B9%E9%87%8F%E4%B8%8B%E8%BD%BD%E3%80%91GDR1624-%E8%A5%BF%E5%8C%97%E5%86%9C%E6%9E%97%E7%A7%91%E6%8A%80%E5%A4%A7%E5%AD%A6%E5%8A%A8%E7%89%A9%E5%8C%BB%E5%AD%A6%E9%99%A24%E4%BE%8B%E7%9C%9F%E8%8F%8C%E8%BD%AC%E5%BD%95%E7%BB%84%E6%B5%8B%E5%BA%8F%E9%A1%B9%E7%9B%AE-%E5%89%94%E9%99%A4T2%E6%A0%B7%E5%93%81-Denovo_%E5%AE%8C%E6%95%B4%E7%89%88%E7%BB%93%E9%A2%98%E6%8A%A5%E5%91%8A%E7%AD%89\GDR1624-%E8%A5%BF%E5%8C%97%E5%86%9C%E6%9E%97%E7%A7%91%E6%8A%80%E5%A4%A7%E5%AD%A6%E5%8A%A8%E7%89%A9%E5%8C%BB%E5%AD%A6%E9%99%A24%E4%BE%8B%E7%9C%9F%E8%8F%8C%E8%BD%AC%E5%BD%95%E7%BB%84%E6%B5%8B%E5%BA%8F%E9%A1%B9%E7%9B%AE-Denovo-RNAseq_result\4_basic_annotation\KEGG\Trichophyton_mentagrophytes-Unigene.fa.htm#gene5) | 113 (4.93%) | ko00190 |
| 6 | [Cell cycle - yeast](file:///G:\WEN\%E8%BD%AC%E5%BD%95%E7%BB%84%E6%B5%8B%E5%BA%8F\%E3%80%90%E6%89%B9%E9%87%8F%E4%B8%8B%E8%BD%BD%E3%80%91GDR1624-%E8%A5%BF%E5%8C%97%E5%86%9C%E6%9E%97%E7%A7%91%E6%8A%80%E5%A4%A7%E5%AD%A6%E5%8A%A8%E7%89%A9%E5%8C%BB%E5%AD%A6%E9%99%A24%E4%BE%8B%E7%9C%9F%E8%8F%8C%E8%BD%AC%E5%BD%95%E7%BB%84%E6%B5%8B%E5%BA%8F%E9%A1%B9%E7%9B%AE-%E5%89%94%E9%99%A4T2%E6%A0%B7%E5%93%81-Denovo_%E5%AE%8C%E6%95%B4%E7%89%88%E7%BB%93%E9%A2%98%E6%8A%A5%E5%91%8A%E7%AD%89\GDR1624-%E8%A5%BF%E5%8C%97%E5%86%9C%E6%9E%97%E7%A7%91%E6%8A%80%E5%A4%A7%E5%AD%A6%E5%8A%A8%E7%89%A9%E5%8C%BB%E5%AD%A6%E9%99%A24%E4%BE%8B%E7%9C%9F%E8%8F%8C%E8%BD%AC%E5%BD%95%E7%BB%84%E6%B5%8B%E5%BA%8F%E9%A1%B9%E7%9B%AE-Denovo-RNAseq_result\4_basic_annotation\KEGG\Trichophyton_mentagrophytes-Unigene.fa.htm#gene6) | 105 (4.58%) | ko04111 |
| 7 | [RNA transport](file:///G:\WEN\%E8%BD%AC%E5%BD%95%E7%BB%84%E6%B5%8B%E5%BA%8F\%E3%80%90%E6%89%B9%E9%87%8F%E4%B8%8B%E8%BD%BD%E3%80%91GDR1624-%E8%A5%BF%E5%8C%97%E5%86%9C%E6%9E%97%E7%A7%91%E6%8A%80%E5%A4%A7%E5%AD%A6%E5%8A%A8%E7%89%A9%E5%8C%BB%E5%AD%A6%E9%99%A24%E4%BE%8B%E7%9C%9F%E8%8F%8C%E8%BD%AC%E5%BD%95%E7%BB%84%E6%B5%8B%E5%BA%8F%E9%A1%B9%E7%9B%AE-%E5%89%94%E9%99%A4T2%E6%A0%B7%E5%93%81-Denovo_%E5%AE%8C%E6%95%B4%E7%89%88%E7%BB%93%E9%A2%98%E6%8A%A5%E5%91%8A%E7%AD%89\GDR1624-%E8%A5%BF%E5%8C%97%E5%86%9C%E6%9E%97%E7%A7%91%E6%8A%80%E5%A4%A7%E5%AD%A6%E5%8A%A8%E7%89%A9%E5%8C%BB%E5%AD%A6%E9%99%A24%E4%BE%8B%E7%9C%9F%E8%8F%8C%E8%BD%AC%E5%BD%95%E7%BB%84%E6%B5%8B%E5%BA%8F%E9%A1%B9%E7%9B%AE-Denovo-RNAseq_result\4_basic_annotation\KEGG\Trichophyton_mentagrophytes-Unigene.fa.htm#gene7) | 99 (4.32%) | ko03013 |
| 8 | [Spliceosome](file:///G:\WEN\%E8%BD%AC%E5%BD%95%E7%BB%84%E6%B5%8B%E5%BA%8F\%E3%80%90%E6%89%B9%E9%87%8F%E4%B8%8B%E8%BD%BD%E3%80%91GDR1624-%E8%A5%BF%E5%8C%97%E5%86%9C%E6%9E%97%E7%A7%91%E6%8A%80%E5%A4%A7%E5%AD%A6%E5%8A%A8%E7%89%A9%E5%8C%BB%E5%AD%A6%E9%99%A24%E4%BE%8B%E7%9C%9F%E8%8F%8C%E8%BD%AC%E5%BD%95%E7%BB%84%E6%B5%8B%E5%BA%8F%E9%A1%B9%E7%9B%AE-%E5%89%94%E9%99%A4T2%E6%A0%B7%E5%93%81-Denovo_%E5%AE%8C%E6%95%B4%E7%89%88%E7%BB%93%E9%A2%98%E6%8A%A5%E5%91%8A%E7%AD%89\GDR1624-%E8%A5%BF%E5%8C%97%E5%86%9C%E6%9E%97%E7%A7%91%E6%8A%80%E5%A4%A7%E5%AD%A6%E5%8A%A8%E7%89%A9%E5%8C%BB%E5%AD%A6%E9%99%A24%E4%BE%8B%E7%9C%9F%E8%8F%8C%E8%BD%AC%E5%BD%95%E7%BB%84%E6%B5%8B%E5%BA%8F%E9%A1%B9%E7%9B%AE-Denovo-RNAseq_result\4_basic_annotation\KEGG\Trichophyton_mentagrophytes-Unigene.fa.htm#gene8) | 94 (4.1%) | ko03040 |
| 9 | [Protein processing in endoplasmic reticulum](file:///G:\WEN\%E8%BD%AC%E5%BD%95%E7%BB%84%E6%B5%8B%E5%BA%8F\%E3%80%90%E6%89%B9%E9%87%8F%E4%B8%8B%E8%BD%BD%E3%80%91GDR1624-%E8%A5%BF%E5%8C%97%E5%86%9C%E6%9E%97%E7%A7%91%E6%8A%80%E5%A4%A7%E5%AD%A6%E5%8A%A8%E7%89%A9%E5%8C%BB%E5%AD%A6%E9%99%A24%E4%BE%8B%E7%9C%9F%E8%8F%8C%E8%BD%AC%E5%BD%95%E7%BB%84%E6%B5%8B%E5%BA%8F%E9%A1%B9%E7%9B%AE-%E5%89%94%E9%99%A4T2%E6%A0%B7%E5%93%81-Denovo_%E5%AE%8C%E6%95%B4%E7%89%88%E7%BB%93%E9%A2%98%E6%8A%A5%E5%91%8A%E7%AD%89\GDR1624-%E8%A5%BF%E5%8C%97%E5%86%9C%E6%9E%97%E7%A7%91%E6%8A%80%E5%A4%A7%E5%AD%A6%E5%8A%A8%E7%89%A9%E5%8C%BB%E5%AD%A6%E9%99%A24%E4%BE%8B%E7%9C%9F%E8%8F%8C%E8%BD%AC%E5%BD%95%E7%BB%84%E6%B5%8B%E5%BA%8F%E9%A1%B9%E7%9B%AE-Denovo-RNAseq_result\4_basic_annotation\KEGG\Trichophyton_mentagrophytes-Unigene.fa.htm#gene9) | 93 (4.06%) | ko04141 |
| 10 | [Endocytosis](file:///G:\WEN\%E8%BD%AC%E5%BD%95%E7%BB%84%E6%B5%8B%E5%BA%8F\%E3%80%90%E6%89%B9%E9%87%8F%E4%B8%8B%E8%BD%BD%E3%80%91GDR1624-%E8%A5%BF%E5%8C%97%E5%86%9C%E6%9E%97%E7%A7%91%E6%8A%80%E5%A4%A7%E5%AD%A6%E5%8A%A8%E7%89%A9%E5%8C%BB%E5%AD%A6%E9%99%A24%E4%BE%8B%E7%9C%9F%E8%8F%8C%E8%BD%AC%E5%BD%95%E7%BB%84%E6%B5%8B%E5%BA%8F%E9%A1%B9%E7%9B%AE-%E5%89%94%E9%99%A4T2%E6%A0%B7%E5%93%81-Denovo_%E5%AE%8C%E6%95%B4%E7%89%88%E7%BB%93%E9%A2%98%E6%8A%A5%E5%91%8A%E7%AD%89\GDR1624-%E8%A5%BF%E5%8C%97%E5%86%9C%E6%9E%97%E7%A7%91%E6%8A%80%E5%A4%A7%E5%AD%A6%E5%8A%A8%E7%89%A9%E5%8C%BB%E5%AD%A6%E9%99%A24%E4%BE%8B%E7%9C%9F%E8%8F%8C%E8%BD%AC%E5%BD%95%E7%BB%84%E6%B5%8B%E5%BA%8F%E9%A1%B9%E7%9B%AE-Denovo-RNAseq_result\4_basic_annotation\KEGG\Trichophyton_mentagrophytes-Unigene.fa.htm#gene10) | 92 (4.01%) | ko04144 |
| 11 | [Meiosis - yeast](file:///G:\WEN\%E8%BD%AC%E5%BD%95%E7%BB%84%E6%B5%8B%E5%BA%8F\%E3%80%90%E6%89%B9%E9%87%8F%E4%B8%8B%E8%BD%BD%E3%80%91GDR1624-%E8%A5%BF%E5%8C%97%E5%86%9C%E6%9E%97%E7%A7%91%E6%8A%80%E5%A4%A7%E5%AD%A6%E5%8A%A8%E7%89%A9%E5%8C%BB%E5%AD%A6%E9%99%A24%E4%BE%8B%E7%9C%9F%E8%8F%8C%E8%BD%AC%E5%BD%95%E7%BB%84%E6%B5%8B%E5%BA%8F%E9%A1%B9%E7%9B%AE-%E5%89%94%E9%99%A4T2%E6%A0%B7%E5%93%81-Denovo_%E5%AE%8C%E6%95%B4%E7%89%88%E7%BB%93%E9%A2%98%E6%8A%A5%E5%91%8A%E7%AD%89\GDR1624-%E8%A5%BF%E5%8C%97%E5%86%9C%E6%9E%97%E7%A7%91%E6%8A%80%E5%A4%A7%E5%AD%A6%E5%8A%A8%E7%89%A9%E5%8C%BB%E5%AD%A6%E9%99%A24%E4%BE%8B%E7%9C%9F%E8%8F%8C%E8%BD%AC%E5%BD%95%E7%BB%84%E6%B5%8B%E5%BA%8F%E9%A1%B9%E7%9B%AE-Denovo-RNAseq_result\4_basic_annotation\KEGG\Trichophyton_mentagrophytes-Unigene.fa.htm#gene11) | 82 (3.58%) | ko04113 |
| 12 | [Pyrimidine metabolism](file:///G:\WEN\%E8%BD%AC%E5%BD%95%E7%BB%84%E6%B5%8B%E5%BA%8F\%E3%80%90%E6%89%B9%E9%87%8F%E4%B8%8B%E8%BD%BD%E3%80%91GDR1624-%E8%A5%BF%E5%8C%97%E5%86%9C%E6%9E%97%E7%A7%91%E6%8A%80%E5%A4%A7%E5%AD%A6%E5%8A%A8%E7%89%A9%E5%8C%BB%E5%AD%A6%E9%99%A24%E4%BE%8B%E7%9C%9F%E8%8F%8C%E8%BD%AC%E5%BD%95%E7%BB%84%E6%B5%8B%E5%BA%8F%E9%A1%B9%E7%9B%AE-%E5%89%94%E9%99%A4T2%E6%A0%B7%E5%93%81-Denovo_%E5%AE%8C%E6%95%B4%E7%89%88%E7%BB%93%E9%A2%98%E6%8A%A5%E5%91%8A%E7%AD%89\GDR1624-%E8%A5%BF%E5%8C%97%E5%86%9C%E6%9E%97%E7%A7%91%E6%8A%80%E5%A4%A7%E5%AD%A6%E5%8A%A8%E7%89%A9%E5%8C%BB%E5%AD%A6%E9%99%A24%E4%BE%8B%E7%9C%9F%E8%8F%8C%E8%BD%AC%E5%BD%95%E7%BB%84%E6%B5%8B%E5%BA%8F%E9%A1%B9%E7%9B%AE-Denovo-RNAseq_result\4_basic_annotation\KEGG\Trichophyton_mentagrophytes-Unigene.fa.htm#gene12) | 82 (3.58%) | ko00240 |
| 13 | [Ribosome biogenesis in eukaryotes](file:///G:\WEN\%E8%BD%AC%E5%BD%95%E7%BB%84%E6%B5%8B%E5%BA%8F\%E3%80%90%E6%89%B9%E9%87%8F%E4%B8%8B%E8%BD%BD%E3%80%91GDR1624-%E8%A5%BF%E5%8C%97%E5%86%9C%E6%9E%97%E7%A7%91%E6%8A%80%E5%A4%A7%E5%AD%A6%E5%8A%A8%E7%89%A9%E5%8C%BB%E5%AD%A6%E9%99%A24%E4%BE%8B%E7%9C%9F%E8%8F%8C%E8%BD%AC%E5%BD%95%E7%BB%84%E6%B5%8B%E5%BA%8F%E9%A1%B9%E7%9B%AE-%E5%89%94%E9%99%A4T2%E6%A0%B7%E5%93%81-Denovo_%E5%AE%8C%E6%95%B4%E7%89%88%E7%BB%93%E9%A2%98%E6%8A%A5%E5%91%8A%E7%AD%89\GDR1624-%E8%A5%BF%E5%8C%97%E5%86%9C%E6%9E%97%E7%A7%91%E6%8A%80%E5%A4%A7%E5%AD%A6%E5%8A%A8%E7%89%A9%E5%8C%BB%E5%AD%A6%E9%99%A24%E4%BE%8B%E7%9C%9F%E8%8F%8C%E8%BD%AC%E5%BD%95%E7%BB%84%E6%B5%8B%E5%BA%8F%E9%A1%B9%E7%9B%AE-Denovo-RNAseq_result\4_basic_annotation\KEGG\Trichophyton_mentagrophytes-Unigene.fa.htm#gene13) | 78 (3.4%) | ko03008 |
| 14 | [Ubiquitin mediated proteolysis](file:///G:\WEN\%E8%BD%AC%E5%BD%95%E7%BB%84%E6%B5%8B%E5%BA%8F\%E3%80%90%E6%89%B9%E9%87%8F%E4%B8%8B%E8%BD%BD%E3%80%91GDR1624-%E8%A5%BF%E5%8C%97%E5%86%9C%E6%9E%97%E7%A7%91%E6%8A%80%E5%A4%A7%E5%AD%A6%E5%8A%A8%E7%89%A9%E5%8C%BB%E5%AD%A6%E9%99%A24%E4%BE%8B%E7%9C%9F%E8%8F%8C%E8%BD%AC%E5%BD%95%E7%BB%84%E6%B5%8B%E5%BA%8F%E9%A1%B9%E7%9B%AE-%E5%89%94%E9%99%A4T2%E6%A0%B7%E5%93%81-Denovo_%E5%AE%8C%E6%95%B4%E7%89%88%E7%BB%93%E9%A2%98%E6%8A%A5%E5%91%8A%E7%AD%89\GDR1624-%E8%A5%BF%E5%8C%97%E5%86%9C%E6%9E%97%E7%A7%91%E6%8A%80%E5%A4%A7%E5%AD%A6%E5%8A%A8%E7%89%A9%E5%8C%BB%E5%AD%A6%E9%99%A24%E4%BE%8B%E7%9C%9F%E8%8F%8C%E8%BD%AC%E5%BD%95%E7%BB%84%E6%B5%8B%E5%BA%8F%E9%A1%B9%E7%9B%AE-Denovo-RNAseq_result\4_basic_annotation\KEGG\Trichophyton_mentagrophytes-Unigene.fa.htm#gene14) | 76 (3.32%) | ko04120 |
| 15 | [Amino sugar and nucleotide sugar metabolism](file:///G:\WEN\%E8%BD%AC%E5%BD%95%E7%BB%84%E6%B5%8B%E5%BA%8F\%E3%80%90%E6%89%B9%E9%87%8F%E4%B8%8B%E8%BD%BD%E3%80%91GDR1624-%E8%A5%BF%E5%8C%97%E5%86%9C%E6%9E%97%E7%A7%91%E6%8A%80%E5%A4%A7%E5%AD%A6%E5%8A%A8%E7%89%A9%E5%8C%BB%E5%AD%A6%E9%99%A24%E4%BE%8B%E7%9C%9F%E8%8F%8C%E8%BD%AC%E5%BD%95%E7%BB%84%E6%B5%8B%E5%BA%8F%E9%A1%B9%E7%9B%AE-%E5%89%94%E9%99%A4T2%E6%A0%B7%E5%93%81-Denovo_%E5%AE%8C%E6%95%B4%E7%89%88%E7%BB%93%E9%A2%98%E6%8A%A5%E5%91%8A%E7%AD%89\GDR1624-%E8%A5%BF%E5%8C%97%E5%86%9C%E6%9E%97%E7%A7%91%E6%8A%80%E5%A4%A7%E5%AD%A6%E5%8A%A8%E7%89%A9%E5%8C%BB%E5%AD%A6%E9%99%A24%E4%BE%8B%E7%9C%9F%E8%8F%8C%E8%BD%AC%E5%BD%95%E7%BB%84%E6%B5%8B%E5%BA%8F%E9%A1%B9%E7%9B%AE-Denovo-RNAseq_result\4_basic_annotation\KEGG\Trichophyton_mentagrophytes-Unigene.fa.htm#gene15) | 76 (3.32%) | ko00520 |
| 16 | [mRNA surveillance pathway](file:///G:\WEN\%E8%BD%AC%E5%BD%95%E7%BB%84%E6%B5%8B%E5%BA%8F\%E3%80%90%E6%89%B9%E9%87%8F%E4%B8%8B%E8%BD%BD%E3%80%91GDR1624-%E8%A5%BF%E5%8C%97%E5%86%9C%E6%9E%97%E7%A7%91%E6%8A%80%E5%A4%A7%E5%AD%A6%E5%8A%A8%E7%89%A9%E5%8C%BB%E5%AD%A6%E9%99%A24%E4%BE%8B%E7%9C%9F%E8%8F%8C%E8%BD%AC%E5%BD%95%E7%BB%84%E6%B5%8B%E5%BA%8F%E9%A1%B9%E7%9B%AE-%E5%89%94%E9%99%A4T2%E6%A0%B7%E5%93%81-Denovo_%E5%AE%8C%E6%95%B4%E7%89%88%E7%BB%93%E9%A2%98%E6%8A%A5%E5%91%8A%E7%AD%89\GDR1624-%E8%A5%BF%E5%8C%97%E5%86%9C%E6%9E%97%E7%A7%91%E6%8A%80%E5%A4%A7%E5%AD%A6%E5%8A%A8%E7%89%A9%E5%8C%BB%E5%AD%A6%E9%99%A24%E4%BE%8B%E7%9C%9F%E8%8F%8C%E8%BD%AC%E5%BD%95%E7%BB%84%E6%B5%8B%E5%BA%8F%E9%A1%B9%E7%9B%AE-Denovo-RNAseq_result\4_basic_annotation\KEGG\Trichophyton_mentagrophytes-Unigene.fa.htm#gene16) | 65 (2.84%) | ko03015 |
| 17 | [RNA degradation](file:///G:\WEN\%E8%BD%AC%E5%BD%95%E7%BB%84%E6%B5%8B%E5%BA%8F\%E3%80%90%E6%89%B9%E9%87%8F%E4%B8%8B%E8%BD%BD%E3%80%91GDR1624-%E8%A5%BF%E5%8C%97%E5%86%9C%E6%9E%97%E7%A7%91%E6%8A%80%E5%A4%A7%E5%AD%A6%E5%8A%A8%E7%89%A9%E5%8C%BB%E5%AD%A6%E9%99%A24%E4%BE%8B%E7%9C%9F%E8%8F%8C%E8%BD%AC%E5%BD%95%E7%BB%84%E6%B5%8B%E5%BA%8F%E9%A1%B9%E7%9B%AE-%E5%89%94%E9%99%A4T2%E6%A0%B7%E5%93%81-Denovo_%E5%AE%8C%E6%95%B4%E7%89%88%E7%BB%93%E9%A2%98%E6%8A%A5%E5%91%8A%E7%AD%89\GDR1624-%E8%A5%BF%E5%8C%97%E5%86%9C%E6%9E%97%E7%A7%91%E6%8A%80%E5%A4%A7%E5%AD%A6%E5%8A%A8%E7%89%A9%E5%8C%BB%E5%AD%A6%E9%99%A24%E4%BE%8B%E7%9C%9F%E8%8F%8C%E8%BD%AC%E5%BD%95%E7%BB%84%E6%B5%8B%E5%BA%8F%E9%A1%B9%E7%9B%AE-Denovo-RNAseq_result\4_basic_annotation\KEGG\Trichophyton_mentagrophytes-Unigene.fa.htm#gene17) | 59 (2.57%) | ko03018 |
| 18 | [Aminoacyl-tRNA biosynthesis](file:///G:\WEN\%E8%BD%AC%E5%BD%95%E7%BB%84%E6%B5%8B%E5%BA%8F\%E3%80%90%E6%89%B9%E9%87%8F%E4%B8%8B%E8%BD%BD%E3%80%91GDR1624-%E8%A5%BF%E5%8C%97%E5%86%9C%E6%9E%97%E7%A7%91%E6%8A%80%E5%A4%A7%E5%AD%A6%E5%8A%A8%E7%89%A9%E5%8C%BB%E5%AD%A6%E9%99%A24%E4%BE%8B%E7%9C%9F%E8%8F%8C%E8%BD%AC%E5%BD%95%E7%BB%84%E6%B5%8B%E5%BA%8F%E9%A1%B9%E7%9B%AE-%E5%89%94%E9%99%A4T2%E6%A0%B7%E5%93%81-Denovo_%E5%AE%8C%E6%95%B4%E7%89%88%E7%BB%93%E9%A2%98%E6%8A%A5%E5%91%8A%E7%AD%89\GDR1624-%E8%A5%BF%E5%8C%97%E5%86%9C%E6%9E%97%E7%A7%91%E6%8A%80%E5%A4%A7%E5%AD%A6%E5%8A%A8%E7%89%A9%E5%8C%BB%E5%AD%A6%E9%99%A24%E4%BE%8B%E7%9C%9F%E8%8F%8C%E8%BD%AC%E5%BD%95%E7%BB%84%E6%B5%8B%E5%BA%8F%E9%A1%B9%E7%9B%AE-Denovo-RNAseq_result\4_basic_annotation\KEGG\Trichophyton_mentagrophytes-Unigene.fa.htm#gene18) | 58 (2.53%) | ko00970 |
| 19 | [Peroxisome](file:///G:\WEN\%E8%BD%AC%E5%BD%95%E7%BB%84%E6%B5%8B%E5%BA%8F\%E3%80%90%E6%89%B9%E9%87%8F%E4%B8%8B%E8%BD%BD%E3%80%91GDR1624-%E8%A5%BF%E5%8C%97%E5%86%9C%E6%9E%97%E7%A7%91%E6%8A%80%E5%A4%A7%E5%AD%A6%E5%8A%A8%E7%89%A9%E5%8C%BB%E5%AD%A6%E9%99%A24%E4%BE%8B%E7%9C%9F%E8%8F%8C%E8%BD%AC%E5%BD%95%E7%BB%84%E6%B5%8B%E5%BA%8F%E9%A1%B9%E7%9B%AE-%E5%89%94%E9%99%A4T2%E6%A0%B7%E5%93%81-Denovo_%E5%AE%8C%E6%95%B4%E7%89%88%E7%BB%93%E9%A2%98%E6%8A%A5%E5%91%8A%E7%AD%89\GDR1624-%E8%A5%BF%E5%8C%97%E5%86%9C%E6%9E%97%E7%A7%91%E6%8A%80%E5%A4%A7%E5%AD%A6%E5%8A%A8%E7%89%A9%E5%8C%BB%E5%AD%A6%E9%99%A24%E4%BE%8B%E7%9C%9F%E8%8F%8C%E8%BD%AC%E5%BD%95%E7%BB%84%E6%B5%8B%E5%BA%8F%E9%A1%B9%E7%9B%AE-Denovo-RNAseq_result\4_basic_annotation\KEGG\Trichophyton_mentagrophytes-Unigene.fa.htm#gene19) | 58 (2.53%) | ko04146 |
| 20 | [Glycerophospholipid metabolism](file:///G:\WEN\%E8%BD%AC%E5%BD%95%E7%BB%84%E6%B5%8B%E5%BA%8F\%E3%80%90%E6%89%B9%E9%87%8F%E4%B8%8B%E8%BD%BD%E3%80%91GDR1624-%E8%A5%BF%E5%8C%97%E5%86%9C%E6%9E%97%E7%A7%91%E6%8A%80%E5%A4%A7%E5%AD%A6%E5%8A%A8%E7%89%A9%E5%8C%BB%E5%AD%A6%E9%99%A24%E4%BE%8B%E7%9C%9F%E8%8F%8C%E8%BD%AC%E5%BD%95%E7%BB%84%E6%B5%8B%E5%BA%8F%E9%A1%B9%E7%9B%AE-%E5%89%94%E9%99%A4T2%E6%A0%B7%E5%93%81-Denovo_%E5%AE%8C%E6%95%B4%E7%89%88%E7%BB%93%E9%A2%98%E6%8A%A5%E5%91%8A%E7%AD%89\GDR1624-%E8%A5%BF%E5%8C%97%E5%86%9C%E6%9E%97%E7%A7%91%E6%8A%80%E5%A4%A7%E5%AD%A6%E5%8A%A8%E7%89%A9%E5%8C%BB%E5%AD%A6%E9%99%A24%E4%BE%8B%E7%9C%9F%E8%8F%8C%E8%BD%AC%E5%BD%95%E7%BB%84%E6%B5%8B%E5%BA%8F%E9%A1%B9%E7%9B%AE-Denovo-RNAseq_result\4_basic_annotation\KEGG\Trichophyton_mentagrophytes-Unigene.fa.htm#gene20) | 53 (2.31%) | ko00564 |
| 21 | [Cysteine and methionine metabolism](file:///G:\WEN\%E8%BD%AC%E5%BD%95%E7%BB%84%E6%B5%8B%E5%BA%8F\%E3%80%90%E6%89%B9%E9%87%8F%E4%B8%8B%E8%BD%BD%E3%80%91GDR1624-%E8%A5%BF%E5%8C%97%E5%86%9C%E6%9E%97%E7%A7%91%E6%8A%80%E5%A4%A7%E5%AD%A6%E5%8A%A8%E7%89%A9%E5%8C%BB%E5%AD%A6%E9%99%A24%E4%BE%8B%E7%9C%9F%E8%8F%8C%E8%BD%AC%E5%BD%95%E7%BB%84%E6%B5%8B%E5%BA%8F%E9%A1%B9%E7%9B%AE-%E5%89%94%E9%99%A4T2%E6%A0%B7%E5%93%81-Denovo_%E5%AE%8C%E6%95%B4%E7%89%88%E7%BB%93%E9%A2%98%E6%8A%A5%E5%91%8A%E7%AD%89\GDR1624-%E8%A5%BF%E5%8C%97%E5%86%9C%E6%9E%97%E7%A7%91%E6%8A%80%E5%A4%A7%E5%AD%A6%E5%8A%A8%E7%89%A9%E5%8C%BB%E5%AD%A6%E9%99%A24%E4%BE%8B%E7%9C%9F%E8%8F%8C%E8%BD%AC%E5%BD%95%E7%BB%84%E6%B5%8B%E5%BA%8F%E9%A1%B9%E7%9B%AE-Denovo-RNAseq_result\4_basic_annotation\KEGG\Trichophyton_mentagrophytes-Unigene.fa.htm#gene21) | 51 (2.23%) | ko00270 |
| 22 | [DNA replication](file:///G:\WEN\%E8%BD%AC%E5%BD%95%E7%BB%84%E6%B5%8B%E5%BA%8F\%E3%80%90%E6%89%B9%E9%87%8F%E4%B8%8B%E8%BD%BD%E3%80%91GDR1624-%E8%A5%BF%E5%8C%97%E5%86%9C%E6%9E%97%E7%A7%91%E6%8A%80%E5%A4%A7%E5%AD%A6%E5%8A%A8%E7%89%A9%E5%8C%BB%E5%AD%A6%E9%99%A24%E4%BE%8B%E7%9C%9F%E8%8F%8C%E8%BD%AC%E5%BD%95%E7%BB%84%E6%B5%8B%E5%BA%8F%E9%A1%B9%E7%9B%AE-%E5%89%94%E9%99%A4T2%E6%A0%B7%E5%93%81-Denovo_%E5%AE%8C%E6%95%B4%E7%89%88%E7%BB%93%E9%A2%98%E6%8A%A5%E5%91%8A%E7%AD%89\GDR1624-%E8%A5%BF%E5%8C%97%E5%86%9C%E6%9E%97%E7%A7%91%E6%8A%80%E5%A4%A7%E5%AD%A6%E5%8A%A8%E7%89%A9%E5%8C%BB%E5%AD%A6%E9%99%A24%E4%BE%8B%E7%9C%9F%E8%8F%8C%E8%BD%AC%E5%BD%95%E7%BB%84%E6%B5%8B%E5%BA%8F%E9%A1%B9%E7%9B%AE-Denovo-RNAseq_result\4_basic_annotation\KEGG\Trichophyton_mentagrophytes-Unigene.fa.htm#gene22) | 50 (2.18%) | ko03030 |
| 23 | [Phagosome](file:///G:\WEN\%E8%BD%AC%E5%BD%95%E7%BB%84%E6%B5%8B%E5%BA%8F\%E3%80%90%E6%89%B9%E9%87%8F%E4%B8%8B%E8%BD%BD%E3%80%91GDR1624-%E8%A5%BF%E5%8C%97%E5%86%9C%E6%9E%97%E7%A7%91%E6%8A%80%E5%A4%A7%E5%AD%A6%E5%8A%A8%E7%89%A9%E5%8C%BB%E5%AD%A6%E9%99%A24%E4%BE%8B%E7%9C%9F%E8%8F%8C%E8%BD%AC%E5%BD%95%E7%BB%84%E6%B5%8B%E5%BA%8F%E9%A1%B9%E7%9B%AE-%E5%89%94%E9%99%A4T2%E6%A0%B7%E5%93%81-Denovo_%E5%AE%8C%E6%95%B4%E7%89%88%E7%BB%93%E9%A2%98%E6%8A%A5%E5%91%8A%E7%AD%89\GDR1624-%E8%A5%BF%E5%8C%97%E5%86%9C%E6%9E%97%E7%A7%91%E6%8A%80%E5%A4%A7%E5%AD%A6%E5%8A%A8%E7%89%A9%E5%8C%BB%E5%AD%A6%E9%99%A24%E4%BE%8B%E7%9C%9F%E8%8F%8C%E8%BD%AC%E5%BD%95%E7%BB%84%E6%B5%8B%E5%BA%8F%E9%A1%B9%E7%9B%AE-Denovo-RNAseq_result\4_basic_annotation\KEGG\Trichophyton_mentagrophytes-Unigene.fa.htm#gene23) | 47 (2.05%) | ko04145 |
| 24 | [Nucleotide excision repair](file:///G:\WEN\%E8%BD%AC%E5%BD%95%E7%BB%84%E6%B5%8B%E5%BA%8F\%E3%80%90%E6%89%B9%E9%87%8F%E4%B8%8B%E8%BD%BD%E3%80%91GDR1624-%E8%A5%BF%E5%8C%97%E5%86%9C%E6%9E%97%E7%A7%91%E6%8A%80%E5%A4%A7%E5%AD%A6%E5%8A%A8%E7%89%A9%E5%8C%BB%E5%AD%A6%E9%99%A24%E4%BE%8B%E7%9C%9F%E8%8F%8C%E8%BD%AC%E5%BD%95%E7%BB%84%E6%B5%8B%E5%BA%8F%E9%A1%B9%E7%9B%AE-%E5%89%94%E9%99%A4T2%E6%A0%B7%E5%93%81-Denovo_%E5%AE%8C%E6%95%B4%E7%89%88%E7%BB%93%E9%A2%98%E6%8A%A5%E5%91%8A%E7%AD%89\GDR1624-%E8%A5%BF%E5%8C%97%E5%86%9C%E6%9E%97%E7%A7%91%E6%8A%80%E5%A4%A7%E5%AD%A6%E5%8A%A8%E7%89%A9%E5%8C%BB%E5%AD%A6%E9%99%A24%E4%BE%8B%E7%9C%9F%E8%8F%8C%E8%BD%AC%E5%BD%95%E7%BB%84%E6%B5%8B%E5%BA%8F%E9%A1%B9%E7%9B%AE-Denovo-RNAseq_result\4_basic_annotation\KEGG\Trichophyton_mentagrophytes-Unigene.fa.htm#gene24) | 46 (2.01%) | ko03420 |
| 25 | [Glycine, serine and threonine metabolism](file:///G:\WEN\%E8%BD%AC%E5%BD%95%E7%BB%84%E6%B5%8B%E5%BA%8F\%E3%80%90%E6%89%B9%E9%87%8F%E4%B8%8B%E8%BD%BD%E3%80%91GDR1624-%E8%A5%BF%E5%8C%97%E5%86%9C%E6%9E%97%E7%A7%91%E6%8A%80%E5%A4%A7%E5%AD%A6%E5%8A%A8%E7%89%A9%E5%8C%BB%E5%AD%A6%E9%99%A24%E4%BE%8B%E7%9C%9F%E8%8F%8C%E8%BD%AC%E5%BD%95%E7%BB%84%E6%B5%8B%E5%BA%8F%E9%A1%B9%E7%9B%AE-%E5%89%94%E9%99%A4T2%E6%A0%B7%E5%93%81-Denovo_%E5%AE%8C%E6%95%B4%E7%89%88%E7%BB%93%E9%A2%98%E6%8A%A5%E5%91%8A%E7%AD%89\GDR1624-%E8%A5%BF%E5%8C%97%E5%86%9C%E6%9E%97%E7%A7%91%E6%8A%80%E5%A4%A7%E5%AD%A6%E5%8A%A8%E7%89%A9%E5%8C%BB%E5%AD%A6%E9%99%A24%E4%BE%8B%E7%9C%9F%E8%8F%8C%E8%BD%AC%E5%BD%95%E7%BB%84%E6%B5%8B%E5%BA%8F%E9%A1%B9%E7%9B%AE-Denovo-RNAseq_result\4_basic_annotation\KEGG\Trichophyton_mentagrophytes-Unigene.fa.htm#gene25) | 45 (1.96%) | ko00260 |
| 26 | [Valine, leucine and isoleucine degradation](file:///G:\WEN\%E8%BD%AC%E5%BD%95%E7%BB%84%E6%B5%8B%E5%BA%8F\%E3%80%90%E6%89%B9%E9%87%8F%E4%B8%8B%E8%BD%BD%E3%80%91GDR1624-%E8%A5%BF%E5%8C%97%E5%86%9C%E6%9E%97%E7%A7%91%E6%8A%80%E5%A4%A7%E5%AD%A6%E5%8A%A8%E7%89%A9%E5%8C%BB%E5%AD%A6%E9%99%A24%E4%BE%8B%E7%9C%9F%E8%8F%8C%E8%BD%AC%E5%BD%95%E7%BB%84%E6%B5%8B%E5%BA%8F%E9%A1%B9%E7%9B%AE-%E5%89%94%E9%99%A4T2%E6%A0%B7%E5%93%81-Denovo_%E5%AE%8C%E6%95%B4%E7%89%88%E7%BB%93%E9%A2%98%E6%8A%A5%E5%91%8A%E7%AD%89\GDR1624-%E8%A5%BF%E5%8C%97%E5%86%9C%E6%9E%97%E7%A7%91%E6%8A%80%E5%A4%A7%E5%AD%A6%E5%8A%A8%E7%89%A9%E5%8C%BB%E5%AD%A6%E9%99%A24%E4%BE%8B%E7%9C%9F%E8%8F%8C%E8%BD%AC%E5%BD%95%E7%BB%84%E6%B5%8B%E5%BA%8F%E9%A1%B9%E7%9B%AE-Denovo-RNAseq_result\4_basic_annotation\KEGG\Trichophyton_mentagrophytes-Unigene.fa.htm#gene26) | 44 (1.92%) | ko00280 |
| 27 | [Starch and sucrose metabolism](file:///G:\WEN\%E8%BD%AC%E5%BD%95%E7%BB%84%E6%B5%8B%E5%BA%8F\%E3%80%90%E6%89%B9%E9%87%8F%E4%B8%8B%E8%BD%BD%E3%80%91GDR1624-%E8%A5%BF%E5%8C%97%E5%86%9C%E6%9E%97%E7%A7%91%E6%8A%80%E5%A4%A7%E5%AD%A6%E5%8A%A8%E7%89%A9%E5%8C%BB%E5%AD%A6%E9%99%A24%E4%BE%8B%E7%9C%9F%E8%8F%8C%E8%BD%AC%E5%BD%95%E7%BB%84%E6%B5%8B%E5%BA%8F%E9%A1%B9%E7%9B%AE-%E5%89%94%E9%99%A4T2%E6%A0%B7%E5%93%81-Denovo_%E5%AE%8C%E6%95%B4%E7%89%88%E7%BB%93%E9%A2%98%E6%8A%A5%E5%91%8A%E7%AD%89\GDR1624-%E8%A5%BF%E5%8C%97%E5%86%9C%E6%9E%97%E7%A7%91%E6%8A%80%E5%A4%A7%E5%AD%A6%E5%8A%A8%E7%89%A9%E5%8C%BB%E5%AD%A6%E9%99%A24%E4%BE%8B%E7%9C%9F%E8%8F%8C%E8%BD%AC%E5%BD%95%E7%BB%84%E6%B5%8B%E5%BA%8F%E9%A1%B9%E7%9B%AE-Denovo-RNAseq_result\4_basic_annotation\KEGG\Trichophyton_mentagrophytes-Unigene.fa.htm#gene27) | 42 (1.83%) | ko00500 |
| 28 | [Glycolysis / Gluconeogenesis](file:///G:\WEN\%E8%BD%AC%E5%BD%95%E7%BB%84%E6%B5%8B%E5%BA%8F\%E3%80%90%E6%89%B9%E9%87%8F%E4%B8%8B%E8%BD%BD%E3%80%91GDR1624-%E8%A5%BF%E5%8C%97%E5%86%9C%E6%9E%97%E7%A7%91%E6%8A%80%E5%A4%A7%E5%AD%A6%E5%8A%A8%E7%89%A9%E5%8C%BB%E5%AD%A6%E9%99%A24%E4%BE%8B%E7%9C%9F%E8%8F%8C%E8%BD%AC%E5%BD%95%E7%BB%84%E6%B5%8B%E5%BA%8F%E9%A1%B9%E7%9B%AE-%E5%89%94%E9%99%A4T2%E6%A0%B7%E5%93%81-Denovo_%E5%AE%8C%E6%95%B4%E7%89%88%E7%BB%93%E9%A2%98%E6%8A%A5%E5%91%8A%E7%AD%89\GDR1624-%E8%A5%BF%E5%8C%97%E5%86%9C%E6%9E%97%E7%A7%91%E6%8A%80%E5%A4%A7%E5%AD%A6%E5%8A%A8%E7%89%A9%E5%8C%BB%E5%AD%A6%E9%99%A24%E4%BE%8B%E7%9C%9F%E8%8F%8C%E8%BD%AC%E5%BD%95%E7%BB%84%E6%B5%8B%E5%BA%8F%E9%A1%B9%E7%9B%AE-Denovo-RNAseq_result\4_basic_annotation\KEGG\Trichophyton_mentagrophytes-Unigene.fa.htm#gene28) | 42 (1.83%) | ko00010 |
| 29 | [Regulation of mitophagy - yeast](file:///G:\WEN\%E8%BD%AC%E5%BD%95%E7%BB%84%E6%B5%8B%E5%BA%8F\%E3%80%90%E6%89%B9%E9%87%8F%E4%B8%8B%E8%BD%BD%E3%80%91GDR1624-%E8%A5%BF%E5%8C%97%E5%86%9C%E6%9E%97%E7%A7%91%E6%8A%80%E5%A4%A7%E5%AD%A6%E5%8A%A8%E7%89%A9%E5%8C%BB%E5%AD%A6%E9%99%A24%E4%BE%8B%E7%9C%9F%E8%8F%8C%E8%BD%AC%E5%BD%95%E7%BB%84%E6%B5%8B%E5%BA%8F%E9%A1%B9%E7%9B%AE-%E5%89%94%E9%99%A4T2%E6%A0%B7%E5%93%81-Denovo_%E5%AE%8C%E6%95%B4%E7%89%88%E7%BB%93%E9%A2%98%E6%8A%A5%E5%91%8A%E7%AD%89\GDR1624-%E8%A5%BF%E5%8C%97%E5%86%9C%E6%9E%97%E7%A7%91%E6%8A%80%E5%A4%A7%E5%AD%A6%E5%8A%A8%E7%89%A9%E5%8C%BB%E5%AD%A6%E9%99%A24%E4%BE%8B%E7%9C%9F%E8%8F%8C%E8%BD%AC%E5%BD%95%E7%BB%84%E6%B5%8B%E5%BA%8F%E9%A1%B9%E7%9B%AE-Denovo-RNAseq_result\4_basic_annotation\KEGG\Trichophyton_mentagrophytes-Unigene.fa.htm#gene29) | 41 (1.79%) | ko04139 |
| 30 | [MAPK signaling pathway - yeast](file:///G:\WEN\%E8%BD%AC%E5%BD%95%E7%BB%84%E6%B5%8B%E5%BA%8F\%E3%80%90%E6%89%B9%E9%87%8F%E4%B8%8B%E8%BD%BD%E3%80%91GDR1624-%E8%A5%BF%E5%8C%97%E5%86%9C%E6%9E%97%E7%A7%91%E6%8A%80%E5%A4%A7%E5%AD%A6%E5%8A%A8%E7%89%A9%E5%8C%BB%E5%AD%A6%E9%99%A24%E4%BE%8B%E7%9C%9F%E8%8F%8C%E8%BD%AC%E5%BD%95%E7%BB%84%E6%B5%8B%E5%BA%8F%E9%A1%B9%E7%9B%AE-%E5%89%94%E9%99%A4T2%E6%A0%B7%E5%93%81-Denovo_%E5%AE%8C%E6%95%B4%E7%89%88%E7%BB%93%E9%A2%98%E6%8A%A5%E5%91%8A%E7%AD%89\GDR1624-%E8%A5%BF%E5%8C%97%E5%86%9C%E6%9E%97%E7%A7%91%E6%8A%80%E5%A4%A7%E5%AD%A6%E5%8A%A8%E7%89%A9%E5%8C%BB%E5%AD%A6%E9%99%A24%E4%BE%8B%E7%9C%9F%E8%8F%8C%E8%BD%AC%E5%BD%95%E7%BB%84%E6%B5%8B%E5%BA%8F%E9%A1%B9%E7%9B%AE-Denovo-RNAseq_result\4_basic_annotation\KEGG\Trichophyton_mentagrophytes-Unigene.fa.htm#gene30) | 40 (1.75%) | ko04011 |
| 31 | [N-Glycan biosynthesis](file:///G:\WEN\%E8%BD%AC%E5%BD%95%E7%BB%84%E6%B5%8B%E5%BA%8F\%E3%80%90%E6%89%B9%E9%87%8F%E4%B8%8B%E8%BD%BD%E3%80%91GDR1624-%E8%A5%BF%E5%8C%97%E5%86%9C%E6%9E%97%E7%A7%91%E6%8A%80%E5%A4%A7%E5%AD%A6%E5%8A%A8%E7%89%A9%E5%8C%BB%E5%AD%A6%E9%99%A24%E4%BE%8B%E7%9C%9F%E8%8F%8C%E8%BD%AC%E5%BD%95%E7%BB%84%E6%B5%8B%E5%BA%8F%E9%A1%B9%E7%9B%AE-%E5%89%94%E9%99%A4T2%E6%A0%B7%E5%93%81-Denovo_%E5%AE%8C%E6%95%B4%E7%89%88%E7%BB%93%E9%A2%98%E6%8A%A5%E5%91%8A%E7%AD%89\GDR1624-%E8%A5%BF%E5%8C%97%E5%86%9C%E6%9E%97%E7%A7%91%E6%8A%80%E5%A4%A7%E5%AD%A6%E5%8A%A8%E7%89%A9%E5%8C%BB%E5%AD%A6%E9%99%A24%E4%BE%8B%E7%9C%9F%E8%8F%8C%E8%BD%AC%E5%BD%95%E7%BB%84%E6%B5%8B%E5%BA%8F%E9%A1%B9%E7%9B%AE-Denovo-RNAseq_result\4_basic_annotation\KEGG\Trichophyton_mentagrophytes-Unigene.fa.htm#gene31) | 40 (1.75%) | ko00510 |
| 32 | [Alanine, aspartate and glutamate metabolism](file:///G:\WEN\%E8%BD%AC%E5%BD%95%E7%BB%84%E6%B5%8B%E5%BA%8F\%E3%80%90%E6%89%B9%E9%87%8F%E4%B8%8B%E8%BD%BD%E3%80%91GDR1624-%E8%A5%BF%E5%8C%97%E5%86%9C%E6%9E%97%E7%A7%91%E6%8A%80%E5%A4%A7%E5%AD%A6%E5%8A%A8%E7%89%A9%E5%8C%BB%E5%AD%A6%E9%99%A24%E4%BE%8B%E7%9C%9F%E8%8F%8C%E8%BD%AC%E5%BD%95%E7%BB%84%E6%B5%8B%E5%BA%8F%E9%A1%B9%E7%9B%AE-%E5%89%94%E9%99%A4T2%E6%A0%B7%E5%93%81-Denovo_%E5%AE%8C%E6%95%B4%E7%89%88%E7%BB%93%E9%A2%98%E6%8A%A5%E5%91%8A%E7%AD%89\GDR1624-%E8%A5%BF%E5%8C%97%E5%86%9C%E6%9E%97%E7%A7%91%E6%8A%80%E5%A4%A7%E5%AD%A6%E5%8A%A8%E7%89%A9%E5%8C%BB%E5%AD%A6%E9%99%A24%E4%BE%8B%E7%9C%9F%E8%8F%8C%E8%BD%AC%E5%BD%95%E7%BB%84%E6%B5%8B%E5%BA%8F%E9%A1%B9%E7%9B%AE-Denovo-RNAseq_result\4_basic_annotation\KEGG\Trichophyton_mentagrophytes-Unigene.fa.htm#gene32) | 40 (1.75%) | ko00250 |
| 33 | [Tryptophan metabolism](file:///G:\WEN\%E8%BD%AC%E5%BD%95%E7%BB%84%E6%B5%8B%E5%BA%8F\%E3%80%90%E6%89%B9%E9%87%8F%E4%B8%8B%E8%BD%BD%E3%80%91GDR1624-%E8%A5%BF%E5%8C%97%E5%86%9C%E6%9E%97%E7%A7%91%E6%8A%80%E5%A4%A7%E5%AD%A6%E5%8A%A8%E7%89%A9%E5%8C%BB%E5%AD%A6%E9%99%A24%E4%BE%8B%E7%9C%9F%E8%8F%8C%E8%BD%AC%E5%BD%95%E7%BB%84%E6%B5%8B%E5%BA%8F%E9%A1%B9%E7%9B%AE-%E5%89%94%E9%99%A4T2%E6%A0%B7%E5%93%81-Denovo_%E5%AE%8C%E6%95%B4%E7%89%88%E7%BB%93%E9%A2%98%E6%8A%A5%E5%91%8A%E7%AD%89\GDR1624-%E8%A5%BF%E5%8C%97%E5%86%9C%E6%9E%97%E7%A7%91%E6%8A%80%E5%A4%A7%E5%AD%A6%E5%8A%A8%E7%89%A9%E5%8C%BB%E5%AD%A6%E9%99%A24%E4%BE%8B%E7%9C%9F%E8%8F%8C%E8%BD%AC%E5%BD%95%E7%BB%84%E6%B5%8B%E5%BA%8F%E9%A1%B9%E7%9B%AE-Denovo-RNAseq_result\4_basic_annotation\KEGG\Trichophyton_mentagrophytes-Unigene.fa.htm#gene33) | 38 (1.66%) | ko00380 |
| 34 | [Proteasome](file:///G:\WEN\%E8%BD%AC%E5%BD%95%E7%BB%84%E6%B5%8B%E5%BA%8F\%E3%80%90%E6%89%B9%E9%87%8F%E4%B8%8B%E8%BD%BD%E3%80%91GDR1624-%E8%A5%BF%E5%8C%97%E5%86%9C%E6%9E%97%E7%A7%91%E6%8A%80%E5%A4%A7%E5%AD%A6%E5%8A%A8%E7%89%A9%E5%8C%BB%E5%AD%A6%E9%99%A24%E4%BE%8B%E7%9C%9F%E8%8F%8C%E8%BD%AC%E5%BD%95%E7%BB%84%E6%B5%8B%E5%BA%8F%E9%A1%B9%E7%9B%AE-%E5%89%94%E9%99%A4T2%E6%A0%B7%E5%93%81-Denovo_%E5%AE%8C%E6%95%B4%E7%89%88%E7%BB%93%E9%A2%98%E6%8A%A5%E5%91%8A%E7%AD%89\GDR1624-%E8%A5%BF%E5%8C%97%E5%86%9C%E6%9E%97%E7%A7%91%E6%8A%80%E5%A4%A7%E5%AD%A6%E5%8A%A8%E7%89%A9%E5%8C%BB%E5%AD%A6%E9%99%A24%E4%BE%8B%E7%9C%9F%E8%8F%8C%E8%BD%AC%E5%BD%95%E7%BB%84%E6%B5%8B%E5%BA%8F%E9%A1%B9%E7%9B%AE-Denovo-RNAseq_result\4_basic_annotation\KEGG\Trichophyton_mentagrophytes-Unigene.fa.htm#gene34) | 38 (1.66%) | ko03050 |
| 35 | [Pyruvate metabolism](file:///G:\WEN\%E8%BD%AC%E5%BD%95%E7%BB%84%E6%B5%8B%E5%BA%8F\%E3%80%90%E6%89%B9%E9%87%8F%E4%B8%8B%E8%BD%BD%E3%80%91GDR1624-%E8%A5%BF%E5%8C%97%E5%86%9C%E6%9E%97%E7%A7%91%E6%8A%80%E5%A4%A7%E5%AD%A6%E5%8A%A8%E7%89%A9%E5%8C%BB%E5%AD%A6%E9%99%A24%E4%BE%8B%E7%9C%9F%E8%8F%8C%E8%BD%AC%E5%BD%95%E7%BB%84%E6%B5%8B%E5%BA%8F%E9%A1%B9%E7%9B%AE-%E5%89%94%E9%99%A4T2%E6%A0%B7%E5%93%81-Denovo_%E5%AE%8C%E6%95%B4%E7%89%88%E7%BB%93%E9%A2%98%E6%8A%A5%E5%91%8A%E7%AD%89\GDR1624-%E8%A5%BF%E5%8C%97%E5%86%9C%E6%9E%97%E7%A7%91%E6%8A%80%E5%A4%A7%E5%AD%A6%E5%8A%A8%E7%89%A9%E5%8C%BB%E5%AD%A6%E9%99%A24%E4%BE%8B%E7%9C%9F%E8%8F%8C%E8%BD%AC%E5%BD%95%E7%BB%84%E6%B5%8B%E5%BA%8F%E9%A1%B9%E7%9B%AE-Denovo-RNAseq_result\4_basic_annotation\KEGG\Trichophyton_mentagrophytes-Unigene.fa.htm#gene35) | 38 (1.66%) | ko00620 |
| 36 | [2-Oxocarboxylic acid metabolism](file:///G:\WEN\%E8%BD%AC%E5%BD%95%E7%BB%84%E6%B5%8B%E5%BA%8F\%E3%80%90%E6%89%B9%E9%87%8F%E4%B8%8B%E8%BD%BD%E3%80%91GDR1624-%E8%A5%BF%E5%8C%97%E5%86%9C%E6%9E%97%E7%A7%91%E6%8A%80%E5%A4%A7%E5%AD%A6%E5%8A%A8%E7%89%A9%E5%8C%BB%E5%AD%A6%E9%99%A24%E4%BE%8B%E7%9C%9F%E8%8F%8C%E8%BD%AC%E5%BD%95%E7%BB%84%E6%B5%8B%E5%BA%8F%E9%A1%B9%E7%9B%AE-%E5%89%94%E9%99%A4T2%E6%A0%B7%E5%93%81-Denovo_%E5%AE%8C%E6%95%B4%E7%89%88%E7%BB%93%E9%A2%98%E6%8A%A5%E5%91%8A%E7%AD%89\GDR1624-%E8%A5%BF%E5%8C%97%E5%86%9C%E6%9E%97%E7%A7%91%E6%8A%80%E5%A4%A7%E5%AD%A6%E5%8A%A8%E7%89%A9%E5%8C%BB%E5%AD%A6%E9%99%A24%E4%BE%8B%E7%9C%9F%E8%8F%8C%E8%BD%AC%E5%BD%95%E7%BB%84%E6%B5%8B%E5%BA%8F%E9%A1%B9%E7%9B%AE-Denovo-RNAseq_result\4_basic_annotation\KEGG\Trichophyton_mentagrophytes-Unigene.fa.htm#gene36) | 37 (1.61%) | ko01210 |
| 37 | [Various types of N-glycan biosynthesis](file:///G:\WEN\%E8%BD%AC%E5%BD%95%E7%BB%84%E6%B5%8B%E5%BA%8F\%E3%80%90%E6%89%B9%E9%87%8F%E4%B8%8B%E8%BD%BD%E3%80%91GDR1624-%E8%A5%BF%E5%8C%97%E5%86%9C%E6%9E%97%E7%A7%91%E6%8A%80%E5%A4%A7%E5%AD%A6%E5%8A%A8%E7%89%A9%E5%8C%BB%E5%AD%A6%E9%99%A24%E4%BE%8B%E7%9C%9F%E8%8F%8C%E8%BD%AC%E5%BD%95%E7%BB%84%E6%B5%8B%E5%BA%8F%E9%A1%B9%E7%9B%AE-%E5%89%94%E9%99%A4T2%E6%A0%B7%E5%93%81-Denovo_%E5%AE%8C%E6%95%B4%E7%89%88%E7%BB%93%E9%A2%98%E6%8A%A5%E5%91%8A%E7%AD%89\GDR1624-%E8%A5%BF%E5%8C%97%E5%86%9C%E6%9E%97%E7%A7%91%E6%8A%80%E5%A4%A7%E5%AD%A6%E5%8A%A8%E7%89%A9%E5%8C%BB%E5%AD%A6%E9%99%A24%E4%BE%8B%E7%9C%9F%E8%8F%8C%E8%BD%AC%E5%BD%95%E7%BB%84%E6%B5%8B%E5%BA%8F%E9%A1%B9%E7%9B%AE-Denovo-RNAseq_result\4_basic_annotation\KEGG\Trichophyton_mentagrophytes-Unigene.fa.htm#gene37) | 37 (1.61%) | ko00513 |
| 38 | [Glutathione metabolism](file:///G:\WEN\%E8%BD%AC%E5%BD%95%E7%BB%84%E6%B5%8B%E5%BA%8F\%E3%80%90%E6%89%B9%E9%87%8F%E4%B8%8B%E8%BD%BD%E3%80%91GDR1624-%E8%A5%BF%E5%8C%97%E5%86%9C%E6%9E%97%E7%A7%91%E6%8A%80%E5%A4%A7%E5%AD%A6%E5%8A%A8%E7%89%A9%E5%8C%BB%E5%AD%A6%E9%99%A24%E4%BE%8B%E7%9C%9F%E8%8F%8C%E8%BD%AC%E5%BD%95%E7%BB%84%E6%B5%8B%E5%BA%8F%E9%A1%B9%E7%9B%AE-%E5%89%94%E9%99%A4T2%E6%A0%B7%E5%93%81-Denovo_%E5%AE%8C%E6%95%B4%E7%89%88%E7%BB%93%E9%A2%98%E6%8A%A5%E5%91%8A%E7%AD%89\GDR1624-%E8%A5%BF%E5%8C%97%E5%86%9C%E6%9E%97%E7%A7%91%E6%8A%80%E5%A4%A7%E5%AD%A6%E5%8A%A8%E7%89%A9%E5%8C%BB%E5%AD%A6%E9%99%A24%E4%BE%8B%E7%9C%9F%E8%8F%8C%E8%BD%AC%E5%BD%95%E7%BB%84%E6%B5%8B%E5%BA%8F%E9%A1%B9%E7%9B%AE-Denovo-RNAseq_result\4_basic_annotation\KEGG\Trichophyton_mentagrophytes-Unigene.fa.htm#gene38) | 36 (1.57%) | ko00480 |
| 39 | [Arginine and proline metabolism](file:///G:\WEN\%E8%BD%AC%E5%BD%95%E7%BB%84%E6%B5%8B%E5%BA%8F\%E3%80%90%E6%89%B9%E9%87%8F%E4%B8%8B%E8%BD%BD%E3%80%91GDR1624-%E8%A5%BF%E5%8C%97%E5%86%9C%E6%9E%97%E7%A7%91%E6%8A%80%E5%A4%A7%E5%AD%A6%E5%8A%A8%E7%89%A9%E5%8C%BB%E5%AD%A6%E9%99%A24%E4%BE%8B%E7%9C%9F%E8%8F%8C%E8%BD%AC%E5%BD%95%E7%BB%84%E6%B5%8B%E5%BA%8F%E9%A1%B9%E7%9B%AE-%E5%89%94%E9%99%A4T2%E6%A0%B7%E5%93%81-Denovo_%E5%AE%8C%E6%95%B4%E7%89%88%E7%BB%93%E9%A2%98%E6%8A%A5%E5%91%8A%E7%AD%89\GDR1624-%E8%A5%BF%E5%8C%97%E5%86%9C%E6%9E%97%E7%A7%91%E6%8A%80%E5%A4%A7%E5%AD%A6%E5%8A%A8%E7%89%A9%E5%8C%BB%E5%AD%A6%E9%99%A24%E4%BE%8B%E7%9C%9F%E8%8F%8C%E8%BD%AC%E5%BD%95%E7%BB%84%E6%B5%8B%E5%BA%8F%E9%A1%B9%E7%9B%AE-Denovo-RNAseq_result\4_basic_annotation\KEGG\Trichophyton_mentagrophytes-Unigene.fa.htm#gene39) | 35 (1.53%) | ko00330 |
| 40 | [Steroid biosynthesis](file:///G:\WEN\%E8%BD%AC%E5%BD%95%E7%BB%84%E6%B5%8B%E5%BA%8F\%E3%80%90%E6%89%B9%E9%87%8F%E4%B8%8B%E8%BD%BD%E3%80%91GDR1624-%E8%A5%BF%E5%8C%97%E5%86%9C%E6%9E%97%E7%A7%91%E6%8A%80%E5%A4%A7%E5%AD%A6%E5%8A%A8%E7%89%A9%E5%8C%BB%E5%AD%A6%E9%99%A24%E4%BE%8B%E7%9C%9F%E8%8F%8C%E8%BD%AC%E5%BD%95%E7%BB%84%E6%B5%8B%E5%BA%8F%E9%A1%B9%E7%9B%AE-%E5%89%94%E9%99%A4T2%E6%A0%B7%E5%93%81-Denovo_%E5%AE%8C%E6%95%B4%E7%89%88%E7%BB%93%E9%A2%98%E6%8A%A5%E5%91%8A%E7%AD%89\GDR1624-%E8%A5%BF%E5%8C%97%E5%86%9C%E6%9E%97%E7%A7%91%E6%8A%80%E5%A4%A7%E5%AD%A6%E5%8A%A8%E7%89%A9%E5%8C%BB%E5%AD%A6%E9%99%A24%E4%BE%8B%E7%9C%9F%E8%8F%8C%E8%BD%AC%E5%BD%95%E7%BB%84%E6%B5%8B%E5%BA%8F%E9%A1%B9%E7%9B%AE-Denovo-RNAseq_result\4_basic_annotation\KEGG\Trichophyton_mentagrophytes-Unigene.fa.htm#gene40) | 34 (1.48%) | ko00100 |
| 41 | [Glyoxylate and dicarboxylate metabolism](file:///G:\WEN\%E8%BD%AC%E5%BD%95%E7%BB%84%E6%B5%8B%E5%BA%8F\%E3%80%90%E6%89%B9%E9%87%8F%E4%B8%8B%E8%BD%BD%E3%80%91GDR1624-%E8%A5%BF%E5%8C%97%E5%86%9C%E6%9E%97%E7%A7%91%E6%8A%80%E5%A4%A7%E5%AD%A6%E5%8A%A8%E7%89%A9%E5%8C%BB%E5%AD%A6%E9%99%A24%E4%BE%8B%E7%9C%9F%E8%8F%8C%E8%BD%AC%E5%BD%95%E7%BB%84%E6%B5%8B%E5%BA%8F%E9%A1%B9%E7%9B%AE-%E5%89%94%E9%99%A4T2%E6%A0%B7%E5%93%81-Denovo_%E5%AE%8C%E6%95%B4%E7%89%88%E7%BB%93%E9%A2%98%E6%8A%A5%E5%91%8A%E7%AD%89\GDR1624-%E8%A5%BF%E5%8C%97%E5%86%9C%E6%9E%97%E7%A7%91%E6%8A%80%E5%A4%A7%E5%AD%A6%E5%8A%A8%E7%89%A9%E5%8C%BB%E5%AD%A6%E9%99%A24%E4%BE%8B%E7%9C%9F%E8%8F%8C%E8%BD%AC%E5%BD%95%E7%BB%84%E6%B5%8B%E5%BA%8F%E9%A1%B9%E7%9B%AE-Denovo-RNAseq_result\4_basic_annotation\KEGG\Trichophyton_mentagrophytes-Unigene.fa.htm#gene41) | 33 (1.44%) | ko00630 |
| 42 | [Citrate cycle (TCA cycle)](file:///G:\WEN\%E8%BD%AC%E5%BD%95%E7%BB%84%E6%B5%8B%E5%BA%8F\%E3%80%90%E6%89%B9%E9%87%8F%E4%B8%8B%E8%BD%BD%E3%80%91GDR1624-%E8%A5%BF%E5%8C%97%E5%86%9C%E6%9E%97%E7%A7%91%E6%8A%80%E5%A4%A7%E5%AD%A6%E5%8A%A8%E7%89%A9%E5%8C%BB%E5%AD%A6%E9%99%A24%E4%BE%8B%E7%9C%9F%E8%8F%8C%E8%BD%AC%E5%BD%95%E7%BB%84%E6%B5%8B%E5%BA%8F%E9%A1%B9%E7%9B%AE-%E5%89%94%E9%99%A4T2%E6%A0%B7%E5%93%81-Denovo_%E5%AE%8C%E6%95%B4%E7%89%88%E7%BB%93%E9%A2%98%E6%8A%A5%E5%91%8A%E7%AD%89\GDR1624-%E8%A5%BF%E5%8C%97%E5%86%9C%E6%9E%97%E7%A7%91%E6%8A%80%E5%A4%A7%E5%AD%A6%E5%8A%A8%E7%89%A9%E5%8C%BB%E5%AD%A6%E9%99%A24%E4%BE%8B%E7%9C%9F%E8%8F%8C%E8%BD%AC%E5%BD%95%E7%BB%84%E6%B5%8B%E5%BA%8F%E9%A1%B9%E7%9B%AE-Denovo-RNAseq_result\4_basic_annotation\KEGG\Trichophyton_mentagrophytes-Unigene.fa.htm#gene42) | 32 (1.4%) | ko00020 |
| 43 | [Pentose phosphate pathway](file:///G:\WEN\%E8%BD%AC%E5%BD%95%E7%BB%84%E6%B5%8B%E5%BA%8F\%E3%80%90%E6%89%B9%E9%87%8F%E4%B8%8B%E8%BD%BD%E3%80%91GDR1624-%E8%A5%BF%E5%8C%97%E5%86%9C%E6%9E%97%E7%A7%91%E6%8A%80%E5%A4%A7%E5%AD%A6%E5%8A%A8%E7%89%A9%E5%8C%BB%E5%AD%A6%E9%99%A24%E4%BE%8B%E7%9C%9F%E8%8F%8C%E8%BD%AC%E5%BD%95%E7%BB%84%E6%B5%8B%E5%BA%8F%E9%A1%B9%E7%9B%AE-%E5%89%94%E9%99%A4T2%E6%A0%B7%E5%93%81-Denovo_%E5%AE%8C%E6%95%B4%E7%89%88%E7%BB%93%E9%A2%98%E6%8A%A5%E5%91%8A%E7%AD%89\GDR1624-%E8%A5%BF%E5%8C%97%E5%86%9C%E6%9E%97%E7%A7%91%E6%8A%80%E5%A4%A7%E5%AD%A6%E5%8A%A8%E7%89%A9%E5%8C%BB%E5%AD%A6%E9%99%A24%E4%BE%8B%E7%9C%9F%E8%8F%8C%E8%BD%AC%E5%BD%95%E7%BB%84%E6%B5%8B%E5%BA%8F%E9%A1%B9%E7%9B%AE-Denovo-RNAseq_result\4_basic_annotation\KEGG\Trichophyton_mentagrophytes-Unigene.fa.htm#gene43) | 31 (1.35%) | ko00030 |
| 44 | [Butanoate metabolism](file:///G:\WEN\%E8%BD%AC%E5%BD%95%E7%BB%84%E6%B5%8B%E5%BA%8F\%E3%80%90%E6%89%B9%E9%87%8F%E4%B8%8B%E8%BD%BD%E3%80%91GDR1624-%E8%A5%BF%E5%8C%97%E5%86%9C%E6%9E%97%E7%A7%91%E6%8A%80%E5%A4%A7%E5%AD%A6%E5%8A%A8%E7%89%A9%E5%8C%BB%E5%AD%A6%E9%99%A24%E4%BE%8B%E7%9C%9F%E8%8F%8C%E8%BD%AC%E5%BD%95%E7%BB%84%E6%B5%8B%E5%BA%8F%E9%A1%B9%E7%9B%AE-%E5%89%94%E9%99%A4T2%E6%A0%B7%E5%93%81-Denovo_%E5%AE%8C%E6%95%B4%E7%89%88%E7%BB%93%E9%A2%98%E6%8A%A5%E5%91%8A%E7%AD%89\GDR1624-%E8%A5%BF%E5%8C%97%E5%86%9C%E6%9E%97%E7%A7%91%E6%8A%80%E5%A4%A7%E5%AD%A6%E5%8A%A8%E7%89%A9%E5%8C%BB%E5%AD%A6%E9%99%A24%E4%BE%8B%E7%9C%9F%E8%8F%8C%E8%BD%AC%E5%BD%95%E7%BB%84%E6%B5%8B%E5%BA%8F%E9%A1%B9%E7%9B%AE-Denovo-RNAseq_result\4_basic_annotation\KEGG\Trichophyton_mentagrophytes-Unigene.fa.htm#gene44) | 31 (1.35%) | ko00650 |
| 45 | [Basal transcription factors](file:///G:\WEN\%E8%BD%AC%E5%BD%95%E7%BB%84%E6%B5%8B%E5%BA%8F\%E3%80%90%E6%89%B9%E9%87%8F%E4%B8%8B%E8%BD%BD%E3%80%91GDR1624-%E8%A5%BF%E5%8C%97%E5%86%9C%E6%9E%97%E7%A7%91%E6%8A%80%E5%A4%A7%E5%AD%A6%E5%8A%A8%E7%89%A9%E5%8C%BB%E5%AD%A6%E9%99%A24%E4%BE%8B%E7%9C%9F%E8%8F%8C%E8%BD%AC%E5%BD%95%E7%BB%84%E6%B5%8B%E5%BA%8F%E9%A1%B9%E7%9B%AE-%E5%89%94%E9%99%A4T2%E6%A0%B7%E5%93%81-Denovo_%E5%AE%8C%E6%95%B4%E7%89%88%E7%BB%93%E9%A2%98%E6%8A%A5%E5%91%8A%E7%AD%89\GDR1624-%E8%A5%BF%E5%8C%97%E5%86%9C%E6%9E%97%E7%A7%91%E6%8A%80%E5%A4%A7%E5%AD%A6%E5%8A%A8%E7%89%A9%E5%8C%BB%E5%AD%A6%E9%99%A24%E4%BE%8B%E7%9C%9F%E8%8F%8C%E8%BD%AC%E5%BD%95%E7%BB%84%E6%B5%8B%E5%BA%8F%E9%A1%B9%E7%9B%AE-Denovo-RNAseq_result\4_basic_annotation\KEGG\Trichophyton_mentagrophytes-Unigene.fa.htm#gene45) | 30 (1.31%) | ko03022 |
| 46 | [RNA polymerase](file:///G:\WEN\%E8%BD%AC%E5%BD%95%E7%BB%84%E6%B5%8B%E5%BA%8F\%E3%80%90%E6%89%B9%E9%87%8F%E4%B8%8B%E8%BD%BD%E3%80%91GDR1624-%E8%A5%BF%E5%8C%97%E5%86%9C%E6%9E%97%E7%A7%91%E6%8A%80%E5%A4%A7%E5%AD%A6%E5%8A%A8%E7%89%A9%E5%8C%BB%E5%AD%A6%E9%99%A24%E4%BE%8B%E7%9C%9F%E8%8F%8C%E8%BD%AC%E5%BD%95%E7%BB%84%E6%B5%8B%E5%BA%8F%E9%A1%B9%E7%9B%AE-%E5%89%94%E9%99%A4T2%E6%A0%B7%E5%93%81-Denovo_%E5%AE%8C%E6%95%B4%E7%89%88%E7%BB%93%E9%A2%98%E6%8A%A5%E5%91%8A%E7%AD%89\GDR1624-%E8%A5%BF%E5%8C%97%E5%86%9C%E6%9E%97%E7%A7%91%E6%8A%80%E5%A4%A7%E5%AD%A6%E5%8A%A8%E7%89%A9%E5%8C%BB%E5%AD%A6%E9%99%A24%E4%BE%8B%E7%9C%9F%E8%8F%8C%E8%BD%AC%E5%BD%95%E7%BB%84%E6%B5%8B%E5%BA%8F%E9%A1%B9%E7%9B%AE-Denovo-RNAseq_result\4_basic_annotation\KEGG\Trichophyton_mentagrophytes-Unigene.fa.htm#gene46) | 29 (1.27%) | ko03020 |
| 47 | [Fatty acid metabolism](file:///G:\WEN\%E8%BD%AC%E5%BD%95%E7%BB%84%E6%B5%8B%E5%BA%8F\%E3%80%90%E6%89%B9%E9%87%8F%E4%B8%8B%E8%BD%BD%E3%80%91GDR1624-%E8%A5%BF%E5%8C%97%E5%86%9C%E6%9E%97%E7%A7%91%E6%8A%80%E5%A4%A7%E5%AD%A6%E5%8A%A8%E7%89%A9%E5%8C%BB%E5%AD%A6%E9%99%A24%E4%BE%8B%E7%9C%9F%E8%8F%8C%E8%BD%AC%E5%BD%95%E7%BB%84%E6%B5%8B%E5%BA%8F%E9%A1%B9%E7%9B%AE-%E5%89%94%E9%99%A4T2%E6%A0%B7%E5%93%81-Denovo_%E5%AE%8C%E6%95%B4%E7%89%88%E7%BB%93%E9%A2%98%E6%8A%A5%E5%91%8A%E7%AD%89\GDR1624-%E8%A5%BF%E5%8C%97%E5%86%9C%E6%9E%97%E7%A7%91%E6%8A%80%E5%A4%A7%E5%AD%A6%E5%8A%A8%E7%89%A9%E5%8C%BB%E5%AD%A6%E9%99%A24%E4%BE%8B%E7%9C%9F%E8%8F%8C%E8%BD%AC%E5%BD%95%E7%BB%84%E6%B5%8B%E5%BA%8F%E9%A1%B9%E7%9B%AE-Denovo-RNAseq_result\4_basic_annotation\KEGG\Trichophyton_mentagrophytes-Unigene.fa.htm#gene47) | 29 (1.27%) | ko01212 |
| 48 | [Lysine degradation](file:///G:\WEN\%E8%BD%AC%E5%BD%95%E7%BB%84%E6%B5%8B%E5%BA%8F\%E3%80%90%E6%89%B9%E9%87%8F%E4%B8%8B%E8%BD%BD%E3%80%91GDR1624-%E8%A5%BF%E5%8C%97%E5%86%9C%E6%9E%97%E7%A7%91%E6%8A%80%E5%A4%A7%E5%AD%A6%E5%8A%A8%E7%89%A9%E5%8C%BB%E5%AD%A6%E9%99%A24%E4%BE%8B%E7%9C%9F%E8%8F%8C%E8%BD%AC%E5%BD%95%E7%BB%84%E6%B5%8B%E5%BA%8F%E9%A1%B9%E7%9B%AE-%E5%89%94%E9%99%A4T2%E6%A0%B7%E5%93%81-Denovo_%E5%AE%8C%E6%95%B4%E7%89%88%E7%BB%93%E9%A2%98%E6%8A%A5%E5%91%8A%E7%AD%89\GDR1624-%E8%A5%BF%E5%8C%97%E5%86%9C%E6%9E%97%E7%A7%91%E6%8A%80%E5%A4%A7%E5%AD%A6%E5%8A%A8%E7%89%A9%E5%8C%BB%E5%AD%A6%E9%99%A24%E4%BE%8B%E7%9C%9F%E8%8F%8C%E8%BD%AC%E5%BD%95%E7%BB%84%E6%B5%8B%E5%BA%8F%E9%A1%B9%E7%9B%AE-Denovo-RNAseq_result\4_basic_annotation\KEGG\Trichophyton_mentagrophytes-Unigene.fa.htm#gene48) | 28 (1.22%) | ko00310 |
| 49 | [Mismatch repair](file:///G:\WEN\%E8%BD%AC%E5%BD%95%E7%BB%84%E6%B5%8B%E5%BA%8F\%E3%80%90%E6%89%B9%E9%87%8F%E4%B8%8B%E8%BD%BD%E3%80%91GDR1624-%E8%A5%BF%E5%8C%97%E5%86%9C%E6%9E%97%E7%A7%91%E6%8A%80%E5%A4%A7%E5%AD%A6%E5%8A%A8%E7%89%A9%E5%8C%BB%E5%AD%A6%E9%99%A24%E4%BE%8B%E7%9C%9F%E8%8F%8C%E8%BD%AC%E5%BD%95%E7%BB%84%E6%B5%8B%E5%BA%8F%E9%A1%B9%E7%9B%AE-%E5%89%94%E9%99%A4T2%E6%A0%B7%E5%93%81-Denovo_%E5%AE%8C%E6%95%B4%E7%89%88%E7%BB%93%E9%A2%98%E6%8A%A5%E5%91%8A%E7%AD%89\GDR1624-%E8%A5%BF%E5%8C%97%E5%86%9C%E6%9E%97%E7%A7%91%E6%8A%80%E5%A4%A7%E5%AD%A6%E5%8A%A8%E7%89%A9%E5%8C%BB%E5%AD%A6%E9%99%A24%E4%BE%8B%E7%9C%9F%E8%8F%8C%E8%BD%AC%E5%BD%95%E7%BB%84%E6%B5%8B%E5%BA%8F%E9%A1%B9%E7%9B%AE-Denovo-RNAseq_result\4_basic_annotation\KEGG\Trichophyton_mentagrophytes-Unigene.fa.htm#gene49) | 28 (1.22%) | ko03430 |
| 50 | [Base excision repair](file:///G:\WEN\%E8%BD%AC%E5%BD%95%E7%BB%84%E6%B5%8B%E5%BA%8F\%E3%80%90%E6%89%B9%E9%87%8F%E4%B8%8B%E8%BD%BD%E3%80%91GDR1624-%E8%A5%BF%E5%8C%97%E5%86%9C%E6%9E%97%E7%A7%91%E6%8A%80%E5%A4%A7%E5%AD%A6%E5%8A%A8%E7%89%A9%E5%8C%BB%E5%AD%A6%E9%99%A24%E4%BE%8B%E7%9C%9F%E8%8F%8C%E8%BD%AC%E5%BD%95%E7%BB%84%E6%B5%8B%E5%BA%8F%E9%A1%B9%E7%9B%AE-%E5%89%94%E9%99%A4T2%E6%A0%B7%E5%93%81-Denovo_%E5%AE%8C%E6%95%B4%E7%89%88%E7%BB%93%E9%A2%98%E6%8A%A5%E5%91%8A%E7%AD%89\GDR1624-%E8%A5%BF%E5%8C%97%E5%86%9C%E6%9E%97%E7%A7%91%E6%8A%80%E5%A4%A7%E5%AD%A6%E5%8A%A8%E7%89%A9%E5%8C%BB%E5%AD%A6%E9%99%A24%E4%BE%8B%E7%9C%9F%E8%8F%8C%E8%BD%AC%E5%BD%95%E7%BB%84%E6%B5%8B%E5%BA%8F%E9%A1%B9%E7%9B%AE-Denovo-RNAseq_result\4_basic_annotation\KEGG\Trichophyton_mentagrophytes-Unigene.fa.htm#gene50) | 28 (1.22%) | ko03410 |
| 51 | [Terpenoid backbone biosynthesis](file:///G:\WEN\%E8%BD%AC%E5%BD%95%E7%BB%84%E6%B5%8B%E5%BA%8F\%E3%80%90%E6%89%B9%E9%87%8F%E4%B8%8B%E8%BD%BD%E3%80%91GDR1624-%E8%A5%BF%E5%8C%97%E5%86%9C%E6%9E%97%E7%A7%91%E6%8A%80%E5%A4%A7%E5%AD%A6%E5%8A%A8%E7%89%A9%E5%8C%BB%E5%AD%A6%E9%99%A24%E4%BE%8B%E7%9C%9F%E8%8F%8C%E8%BD%AC%E5%BD%95%E7%BB%84%E6%B5%8B%E5%BA%8F%E9%A1%B9%E7%9B%AE-%E5%89%94%E9%99%A4T2%E6%A0%B7%E5%93%81-Denovo_%E5%AE%8C%E6%95%B4%E7%89%88%E7%BB%93%E9%A2%98%E6%8A%A5%E5%91%8A%E7%AD%89\GDR1624-%E8%A5%BF%E5%8C%97%E5%86%9C%E6%9E%97%E7%A7%91%E6%8A%80%E5%A4%A7%E5%AD%A6%E5%8A%A8%E7%89%A9%E5%8C%BB%E5%AD%A6%E9%99%A24%E4%BE%8B%E7%9C%9F%E8%8F%8C%E8%BD%AC%E5%BD%95%E7%BB%84%E6%B5%8B%E5%BA%8F%E9%A1%B9%E7%9B%AE-Denovo-RNAseq_result\4_basic_annotation\KEGG\Trichophyton_mentagrophytes-Unigene.fa.htm#gene51) | 28 (1.22%) | ko00900 |
| 52 | [Phenylalanine, tyrosine and tryptophan biosynthesis](file:///G:\WEN\%E8%BD%AC%E5%BD%95%E7%BB%84%E6%B5%8B%E5%BA%8F\%E3%80%90%E6%89%B9%E9%87%8F%E4%B8%8B%E8%BD%BD%E3%80%91GDR1624-%E8%A5%BF%E5%8C%97%E5%86%9C%E6%9E%97%E7%A7%91%E6%8A%80%E5%A4%A7%E5%AD%A6%E5%8A%A8%E7%89%A9%E5%8C%BB%E5%AD%A6%E9%99%A24%E4%BE%8B%E7%9C%9F%E8%8F%8C%E8%BD%AC%E5%BD%95%E7%BB%84%E6%B5%8B%E5%BA%8F%E9%A1%B9%E7%9B%AE-%E5%89%94%E9%99%A4T2%E6%A0%B7%E5%93%81-Denovo_%E5%AE%8C%E6%95%B4%E7%89%88%E7%BB%93%E9%A2%98%E6%8A%A5%E5%91%8A%E7%AD%89\GDR1624-%E8%A5%BF%E5%8C%97%E5%86%9C%E6%9E%97%E7%A7%91%E6%8A%80%E5%A4%A7%E5%AD%A6%E5%8A%A8%E7%89%A9%E5%8C%BB%E5%AD%A6%E9%99%A24%E4%BE%8B%E7%9C%9F%E8%8F%8C%E8%BD%AC%E5%BD%95%E7%BB%84%E6%B5%8B%E5%BA%8F%E9%A1%B9%E7%9B%AE-Denovo-RNAseq_result\4_basic_annotation\KEGG\Trichophyton_mentagrophytes-Unigene.fa.htm#gene52) | 27 (1.18%) | ko00400 |
| 53 | [Fructose and mannose metabolism](file:///G:\WEN\%E8%BD%AC%E5%BD%95%E7%BB%84%E6%B5%8B%E5%BA%8F\%E3%80%90%E6%89%B9%E9%87%8F%E4%B8%8B%E8%BD%BD%E3%80%91GDR1624-%E8%A5%BF%E5%8C%97%E5%86%9C%E6%9E%97%E7%A7%91%E6%8A%80%E5%A4%A7%E5%AD%A6%E5%8A%A8%E7%89%A9%E5%8C%BB%E5%AD%A6%E9%99%A24%E4%BE%8B%E7%9C%9F%E8%8F%8C%E8%BD%AC%E5%BD%95%E7%BB%84%E6%B5%8B%E5%BA%8F%E9%A1%B9%E7%9B%AE-%E5%89%94%E9%99%A4T2%E6%A0%B7%E5%93%81-Denovo_%E5%AE%8C%E6%95%B4%E7%89%88%E7%BB%93%E9%A2%98%E6%8A%A5%E5%91%8A%E7%AD%89\GDR1624-%E8%A5%BF%E5%8C%97%E5%86%9C%E6%9E%97%E7%A7%91%E6%8A%80%E5%A4%A7%E5%AD%A6%E5%8A%A8%E7%89%A9%E5%8C%BB%E5%AD%A6%E9%99%A24%E4%BE%8B%E7%9C%9F%E8%8F%8C%E8%BD%AC%E5%BD%95%E7%BB%84%E6%B5%8B%E5%BA%8F%E9%A1%B9%E7%9B%AE-Denovo-RNAseq_result\4_basic_annotation\KEGG\Trichophyton_mentagrophytes-Unigene.fa.htm#gene53) | 27 (1.18%) | ko00051 |
| 54 | [Phenylalanine metabolism](file:///G:\WEN\%E8%BD%AC%E5%BD%95%E7%BB%84%E6%B5%8B%E5%BA%8F\%E3%80%90%E6%89%B9%E9%87%8F%E4%B8%8B%E8%BD%BD%E3%80%91GDR1624-%E8%A5%BF%E5%8C%97%E5%86%9C%E6%9E%97%E7%A7%91%E6%8A%80%E5%A4%A7%E5%AD%A6%E5%8A%A8%E7%89%A9%E5%8C%BB%E5%AD%A6%E9%99%A24%E4%BE%8B%E7%9C%9F%E8%8F%8C%E8%BD%AC%E5%BD%95%E7%BB%84%E6%B5%8B%E5%BA%8F%E9%A1%B9%E7%9B%AE-%E5%89%94%E9%99%A4T2%E6%A0%B7%E5%93%81-Denovo_%E5%AE%8C%E6%95%B4%E7%89%88%E7%BB%93%E9%A2%98%E6%8A%A5%E5%91%8A%E7%AD%89\GDR1624-%E8%A5%BF%E5%8C%97%E5%86%9C%E6%9E%97%E7%A7%91%E6%8A%80%E5%A4%A7%E5%AD%A6%E5%8A%A8%E7%89%A9%E5%8C%BB%E5%AD%A6%E9%99%A24%E4%BE%8B%E7%9C%9F%E8%8F%8C%E8%BD%AC%E5%BD%95%E7%BB%84%E6%B5%8B%E5%BA%8F%E9%A1%B9%E7%9B%AE-Denovo-RNAseq_result\4_basic_annotation\KEGG\Trichophyton_mentagrophytes-Unigene.fa.htm#gene54) | 26 (1.13%) | ko00360 |
| 55 | [Glycerolipid metabolism](file:///G:\WEN\%E8%BD%AC%E5%BD%95%E7%BB%84%E6%B5%8B%E5%BA%8F\%E3%80%90%E6%89%B9%E9%87%8F%E4%B8%8B%E8%BD%BD%E3%80%91GDR1624-%E8%A5%BF%E5%8C%97%E5%86%9C%E6%9E%97%E7%A7%91%E6%8A%80%E5%A4%A7%E5%AD%A6%E5%8A%A8%E7%89%A9%E5%8C%BB%E5%AD%A6%E9%99%A24%E4%BE%8B%E7%9C%9F%E8%8F%8C%E8%BD%AC%E5%BD%95%E7%BB%84%E6%B5%8B%E5%BA%8F%E9%A1%B9%E7%9B%AE-%E5%89%94%E9%99%A4T2%E6%A0%B7%E5%93%81-Denovo_%E5%AE%8C%E6%95%B4%E7%89%88%E7%BB%93%E9%A2%98%E6%8A%A5%E5%91%8A%E7%AD%89\GDR1624-%E8%A5%BF%E5%8C%97%E5%86%9C%E6%9E%97%E7%A7%91%E6%8A%80%E5%A4%A7%E5%AD%A6%E5%8A%A8%E7%89%A9%E5%8C%BB%E5%AD%A6%E9%99%A24%E4%BE%8B%E7%9C%9F%E8%8F%8C%E8%BD%AC%E5%BD%95%E7%BB%84%E6%B5%8B%E5%BA%8F%E9%A1%B9%E7%9B%AE-Denovo-RNAseq_result\4_basic_annotation\KEGG\Trichophyton_mentagrophytes-Unigene.fa.htm#gene55) | 26 (1.13%) | ko00561 |
| 56 | [Homologous recombination](file:///G:\WEN\%E8%BD%AC%E5%BD%95%E7%BB%84%E6%B5%8B%E5%BA%8F\%E3%80%90%E6%89%B9%E9%87%8F%E4%B8%8B%E8%BD%BD%E3%80%91GDR1624-%E8%A5%BF%E5%8C%97%E5%86%9C%E6%9E%97%E7%A7%91%E6%8A%80%E5%A4%A7%E5%AD%A6%E5%8A%A8%E7%89%A9%E5%8C%BB%E5%AD%A6%E9%99%A24%E4%BE%8B%E7%9C%9F%E8%8F%8C%E8%BD%AC%E5%BD%95%E7%BB%84%E6%B5%8B%E5%BA%8F%E9%A1%B9%E7%9B%AE-%E5%89%94%E9%99%A4T2%E6%A0%B7%E5%93%81-Denovo_%E5%AE%8C%E6%95%B4%E7%89%88%E7%BB%93%E9%A2%98%E6%8A%A5%E5%91%8A%E7%AD%89\GDR1624-%E8%A5%BF%E5%8C%97%E5%86%9C%E6%9E%97%E7%A7%91%E6%8A%80%E5%A4%A7%E5%AD%A6%E5%8A%A8%E7%89%A9%E5%8C%BB%E5%AD%A6%E9%99%A24%E4%BE%8B%E7%9C%9F%E8%8F%8C%E8%BD%AC%E5%BD%95%E7%BB%84%E6%B5%8B%E5%BA%8F%E9%A1%B9%E7%9B%AE-Denovo-RNAseq_result\4_basic_annotation\KEGG\Trichophyton_mentagrophytes-Unigene.fa.htm#gene56) | 26 (1.13%) | ko03440 |
| 57 | [Pantothenate and CoA biosynthesis](file:///G:\WEN\%E8%BD%AC%E5%BD%95%E7%BB%84%E6%B5%8B%E5%BA%8F\%E3%80%90%E6%89%B9%E9%87%8F%E4%B8%8B%E8%BD%BD%E3%80%91GDR1624-%E8%A5%BF%E5%8C%97%E5%86%9C%E6%9E%97%E7%A7%91%E6%8A%80%E5%A4%A7%E5%AD%A6%E5%8A%A8%E7%89%A9%E5%8C%BB%E5%AD%A6%E9%99%A24%E4%BE%8B%E7%9C%9F%E8%8F%8C%E8%BD%AC%E5%BD%95%E7%BB%84%E6%B5%8B%E5%BA%8F%E9%A1%B9%E7%9B%AE-%E5%89%94%E9%99%A4T2%E6%A0%B7%E5%93%81-Denovo_%E5%AE%8C%E6%95%B4%E7%89%88%E7%BB%93%E9%A2%98%E6%8A%A5%E5%91%8A%E7%AD%89\GDR1624-%E8%A5%BF%E5%8C%97%E5%86%9C%E6%9E%97%E7%A7%91%E6%8A%80%E5%A4%A7%E5%AD%A6%E5%8A%A8%E7%89%A9%E5%8C%BB%E5%AD%A6%E9%99%A24%E4%BE%8B%E7%9C%9F%E8%8F%8C%E8%BD%AC%E5%BD%95%E7%BB%84%E6%B5%8B%E5%BA%8F%E9%A1%B9%E7%9B%AE-Denovo-RNAseq_result\4_basic_annotation\KEGG\Trichophyton_mentagrophytes-Unigene.fa.htm#gene57) | 25 (1.09%) | ko00770 |
| 58 | [Glycosylphosphatidylinositol(GPI)-anchor biosynthesis](file:///G:\WEN\%E8%BD%AC%E5%BD%95%E7%BB%84%E6%B5%8B%E5%BA%8F\%E3%80%90%E6%89%B9%E9%87%8F%E4%B8%8B%E8%BD%BD%E3%80%91GDR1624-%E8%A5%BF%E5%8C%97%E5%86%9C%E6%9E%97%E7%A7%91%E6%8A%80%E5%A4%A7%E5%AD%A6%E5%8A%A8%E7%89%A9%E5%8C%BB%E5%AD%A6%E9%99%A24%E4%BE%8B%E7%9C%9F%E8%8F%8C%E8%BD%AC%E5%BD%95%E7%BB%84%E6%B5%8B%E5%BA%8F%E9%A1%B9%E7%9B%AE-%E5%89%94%E9%99%A4T2%E6%A0%B7%E5%93%81-Denovo_%E5%AE%8C%E6%95%B4%E7%89%88%E7%BB%93%E9%A2%98%E6%8A%A5%E5%91%8A%E7%AD%89\GDR1624-%E8%A5%BF%E5%8C%97%E5%86%9C%E6%9E%97%E7%A7%91%E6%8A%80%E5%A4%A7%E5%AD%A6%E5%8A%A8%E7%89%A9%E5%8C%BB%E5%AD%A6%E9%99%A24%E4%BE%8B%E7%9C%9F%E8%8F%8C%E8%BD%AC%E5%BD%95%E7%BB%84%E6%B5%8B%E5%BA%8F%E9%A1%B9%E7%9B%AE-Denovo-RNAseq_result\4_basic_annotation\KEGG\Trichophyton_mentagrophytes-Unigene.fa.htm#gene58) | 25 (1.09%) | ko00563 |
| 59 | [Tyrosine metabolism](file:///G:\WEN\%E8%BD%AC%E5%BD%95%E7%BB%84%E6%B5%8B%E5%BA%8F\%E3%80%90%E6%89%B9%E9%87%8F%E4%B8%8B%E8%BD%BD%E3%80%91GDR1624-%E8%A5%BF%E5%8C%97%E5%86%9C%E6%9E%97%E7%A7%91%E6%8A%80%E5%A4%A7%E5%AD%A6%E5%8A%A8%E7%89%A9%E5%8C%BB%E5%AD%A6%E9%99%A24%E4%BE%8B%E7%9C%9F%E8%8F%8C%E8%BD%AC%E5%BD%95%E7%BB%84%E6%B5%8B%E5%BA%8F%E9%A1%B9%E7%9B%AE-%E5%89%94%E9%99%A4T2%E6%A0%B7%E5%93%81-Denovo_%E5%AE%8C%E6%95%B4%E7%89%88%E7%BB%93%E9%A2%98%E6%8A%A5%E5%91%8A%E7%AD%89\GDR1624-%E8%A5%BF%E5%8C%97%E5%86%9C%E6%9E%97%E7%A7%91%E6%8A%80%E5%A4%A7%E5%AD%A6%E5%8A%A8%E7%89%A9%E5%8C%BB%E5%AD%A6%E9%99%A24%E4%BE%8B%E7%9C%9F%E8%8F%8C%E8%BD%AC%E5%BD%95%E7%BB%84%E6%B5%8B%E5%BA%8F%E9%A1%B9%E7%9B%AE-Denovo-RNAseq_result\4_basic_annotation\KEGG\Trichophyton_mentagrophytes-Unigene.fa.htm#gene59) | 23 (1%) | ko00350 |
| 60 | [Valine, leucine and isoleucine biosynthesis](file:///G:\WEN\%E8%BD%AC%E5%BD%95%E7%BB%84%E6%B5%8B%E5%BA%8F\%E3%80%90%E6%89%B9%E9%87%8F%E4%B8%8B%E8%BD%BD%E3%80%91GDR1624-%E8%A5%BF%E5%8C%97%E5%86%9C%E6%9E%97%E7%A7%91%E6%8A%80%E5%A4%A7%E5%AD%A6%E5%8A%A8%E7%89%A9%E5%8C%BB%E5%AD%A6%E9%99%A24%E4%BE%8B%E7%9C%9F%E8%8F%8C%E8%BD%AC%E5%BD%95%E7%BB%84%E6%B5%8B%E5%BA%8F%E9%A1%B9%E7%9B%AE-%E5%89%94%E9%99%A4T2%E6%A0%B7%E5%93%81-Denovo_%E5%AE%8C%E6%95%B4%E7%89%88%E7%BB%93%E9%A2%98%E6%8A%A5%E5%91%8A%E7%AD%89\GDR1624-%E8%A5%BF%E5%8C%97%E5%86%9C%E6%9E%97%E7%A7%91%E6%8A%80%E5%A4%A7%E5%AD%A6%E5%8A%A8%E7%89%A9%E5%8C%BB%E5%AD%A6%E9%99%A24%E4%BE%8B%E7%9C%9F%E8%8F%8C%E8%BD%AC%E5%BD%95%E7%BB%84%E6%B5%8B%E5%BA%8F%E9%A1%B9%E7%9B%AE-Denovo-RNAseq_result\4_basic_annotation\KEGG\Trichophyton_mentagrophytes-Unigene.fa.htm#gene60) | 22 (0.96%) | ko00290 |
| 61 | [Sphingolipid metabolism](file:///G:\WEN\%E8%BD%AC%E5%BD%95%E7%BB%84%E6%B5%8B%E5%BA%8F\%E3%80%90%E6%89%B9%E9%87%8F%E4%B8%8B%E8%BD%BD%E3%80%91GDR1624-%E8%A5%BF%E5%8C%97%E5%86%9C%E6%9E%97%E7%A7%91%E6%8A%80%E5%A4%A7%E5%AD%A6%E5%8A%A8%E7%89%A9%E5%8C%BB%E5%AD%A6%E9%99%A24%E4%BE%8B%E7%9C%9F%E8%8F%8C%E8%BD%AC%E5%BD%95%E7%BB%84%E6%B5%8B%E5%BA%8F%E9%A1%B9%E7%9B%AE-%E5%89%94%E9%99%A4T2%E6%A0%B7%E5%93%81-Denovo_%E5%AE%8C%E6%95%B4%E7%89%88%E7%BB%93%E9%A2%98%E6%8A%A5%E5%91%8A%E7%AD%89\GDR1624-%E8%A5%BF%E5%8C%97%E5%86%9C%E6%9E%97%E7%A7%91%E6%8A%80%E5%A4%A7%E5%AD%A6%E5%8A%A8%E7%89%A9%E5%8C%BB%E5%AD%A6%E9%99%A24%E4%BE%8B%E7%9C%9F%E8%8F%8C%E8%BD%AC%E5%BD%95%E7%BB%84%E6%B5%8B%E5%BA%8F%E9%A1%B9%E7%9B%AE-Denovo-RNAseq_result\4_basic_annotation\KEGG\Trichophyton_mentagrophytes-Unigene.fa.htm#gene61) | 22 (0.96%) | ko00600 |
| 62 | [beta-Alanine metabolism](file:///G:\WEN\%E8%BD%AC%E5%BD%95%E7%BB%84%E6%B5%8B%E5%BA%8F\%E3%80%90%E6%89%B9%E9%87%8F%E4%B8%8B%E8%BD%BD%E3%80%91GDR1624-%E8%A5%BF%E5%8C%97%E5%86%9C%E6%9E%97%E7%A7%91%E6%8A%80%E5%A4%A7%E5%AD%A6%E5%8A%A8%E7%89%A9%E5%8C%BB%E5%AD%A6%E9%99%A24%E4%BE%8B%E7%9C%9F%E8%8F%8C%E8%BD%AC%E5%BD%95%E7%BB%84%E6%B5%8B%E5%BA%8F%E9%A1%B9%E7%9B%AE-%E5%89%94%E9%99%A4T2%E6%A0%B7%E5%93%81-Denovo_%E5%AE%8C%E6%95%B4%E7%89%88%E7%BB%93%E9%A2%98%E6%8A%A5%E5%91%8A%E7%AD%89\GDR1624-%E8%A5%BF%E5%8C%97%E5%86%9C%E6%9E%97%E7%A7%91%E6%8A%80%E5%A4%A7%E5%AD%A6%E5%8A%A8%E7%89%A9%E5%8C%BB%E5%AD%A6%E9%99%A24%E4%BE%8B%E7%9C%9F%E8%8F%8C%E8%BD%AC%E5%BD%95%E7%BB%84%E6%B5%8B%E5%BA%8F%E9%A1%B9%E7%9B%AE-Denovo-RNAseq_result\4_basic_annotation\KEGG\Trichophyton_mentagrophytes-Unigene.fa.htm#gene62) | 22 (0.96%) | ko00410 |
| 63 | [Fatty acid degradation](file:///G:\WEN\%E8%BD%AC%E5%BD%95%E7%BB%84%E6%B5%8B%E5%BA%8F\%E3%80%90%E6%89%B9%E9%87%8F%E4%B8%8B%E8%BD%BD%E3%80%91GDR1624-%E8%A5%BF%E5%8C%97%E5%86%9C%E6%9E%97%E7%A7%91%E6%8A%80%E5%A4%A7%E5%AD%A6%E5%8A%A8%E7%89%A9%E5%8C%BB%E5%AD%A6%E9%99%A24%E4%BE%8B%E7%9C%9F%E8%8F%8C%E8%BD%AC%E5%BD%95%E7%BB%84%E6%B5%8B%E5%BA%8F%E9%A1%B9%E7%9B%AE-%E5%89%94%E9%99%A4T2%E6%A0%B7%E5%93%81-Denovo_%E5%AE%8C%E6%95%B4%E7%89%88%E7%BB%93%E9%A2%98%E6%8A%A5%E5%91%8A%E7%AD%89\GDR1624-%E8%A5%BF%E5%8C%97%E5%86%9C%E6%9E%97%E7%A7%91%E6%8A%80%E5%A4%A7%E5%AD%A6%E5%8A%A8%E7%89%A9%E5%8C%BB%E5%AD%A6%E9%99%A24%E4%BE%8B%E7%9C%9F%E8%8F%8C%E8%BD%AC%E5%BD%95%E7%BB%84%E6%B5%8B%E5%BA%8F%E9%A1%B9%E7%9B%AE-Denovo-RNAseq_result\4_basic_annotation\KEGG\Trichophyton_mentagrophytes-Unigene.fa.htm#gene63) | 21 (0.92%) | ko00071 |
| 64 | [Arginine biosynthesis](file:///G:\WEN\%E8%BD%AC%E5%BD%95%E7%BB%84%E6%B5%8B%E5%BA%8F\%E3%80%90%E6%89%B9%E9%87%8F%E4%B8%8B%E8%BD%BD%E3%80%91GDR1624-%E8%A5%BF%E5%8C%97%E5%86%9C%E6%9E%97%E7%A7%91%E6%8A%80%E5%A4%A7%E5%AD%A6%E5%8A%A8%E7%89%A9%E5%8C%BB%E5%AD%A6%E9%99%A24%E4%BE%8B%E7%9C%9F%E8%8F%8C%E8%BD%AC%E5%BD%95%E7%BB%84%E6%B5%8B%E5%BA%8F%E9%A1%B9%E7%9B%AE-%E5%89%94%E9%99%A4T2%E6%A0%B7%E5%93%81-Denovo_%E5%AE%8C%E6%95%B4%E7%89%88%E7%BB%93%E9%A2%98%E6%8A%A5%E5%91%8A%E7%AD%89\GDR1624-%E8%A5%BF%E5%8C%97%E5%86%9C%E6%9E%97%E7%A7%91%E6%8A%80%E5%A4%A7%E5%AD%A6%E5%8A%A8%E7%89%A9%E5%8C%BB%E5%AD%A6%E9%99%A24%E4%BE%8B%E7%9C%9F%E8%8F%8C%E8%BD%AC%E5%BD%95%E7%BB%84%E6%B5%8B%E5%BA%8F%E9%A1%B9%E7%9B%AE-Denovo-RNAseq_result\4_basic_annotation\KEGG\Trichophyton_mentagrophytes-Unigene.fa.htm#gene64) | 21 (0.92%) | ko00220 |
| 65 | [Propanoate metabolism](file:///G:\WEN\%E8%BD%AC%E5%BD%95%E7%BB%84%E6%B5%8B%E5%BA%8F\%E3%80%90%E6%89%B9%E9%87%8F%E4%B8%8B%E8%BD%BD%E3%80%91GDR1624-%E8%A5%BF%E5%8C%97%E5%86%9C%E6%9E%97%E7%A7%91%E6%8A%80%E5%A4%A7%E5%AD%A6%E5%8A%A8%E7%89%A9%E5%8C%BB%E5%AD%A6%E9%99%A24%E4%BE%8B%E7%9C%9F%E8%8F%8C%E8%BD%AC%E5%BD%95%E7%BB%84%E6%B5%8B%E5%BA%8F%E9%A1%B9%E7%9B%AE-%E5%89%94%E9%99%A4T2%E6%A0%B7%E5%93%81-Denovo_%E5%AE%8C%E6%95%B4%E7%89%88%E7%BB%93%E9%A2%98%E6%8A%A5%E5%91%8A%E7%AD%89\GDR1624-%E8%A5%BF%E5%8C%97%E5%86%9C%E6%9E%97%E7%A7%91%E6%8A%80%E5%A4%A7%E5%AD%A6%E5%8A%A8%E7%89%A9%E5%8C%BB%E5%AD%A6%E9%99%A24%E4%BE%8B%E7%9C%9F%E8%8F%8C%E8%BD%AC%E5%BD%95%E7%BB%84%E6%B5%8B%E5%BA%8F%E9%A1%B9%E7%9B%AE-Denovo-RNAseq_result\4_basic_annotation\KEGG\Trichophyton_mentagrophytes-Unigene.fa.htm#gene65) | 20 (0.87%) | ko00640 |
| 66 | [One carbon pool by folate](file:///G:\WEN\%E8%BD%AC%E5%BD%95%E7%BB%84%E6%B5%8B%E5%BA%8F\%E3%80%90%E6%89%B9%E9%87%8F%E4%B8%8B%E8%BD%BD%E3%80%91GDR1624-%E8%A5%BF%E5%8C%97%E5%86%9C%E6%9E%97%E7%A7%91%E6%8A%80%E5%A4%A7%E5%AD%A6%E5%8A%A8%E7%89%A9%E5%8C%BB%E5%AD%A6%E9%99%A24%E4%BE%8B%E7%9C%9F%E8%8F%8C%E8%BD%AC%E5%BD%95%E7%BB%84%E6%B5%8B%E5%BA%8F%E9%A1%B9%E7%9B%AE-%E5%89%94%E9%99%A4T2%E6%A0%B7%E5%93%81-Denovo_%E5%AE%8C%E6%95%B4%E7%89%88%E7%BB%93%E9%A2%98%E6%8A%A5%E5%91%8A%E7%AD%89\GDR1624-%E8%A5%BF%E5%8C%97%E5%86%9C%E6%9E%97%E7%A7%91%E6%8A%80%E5%A4%A7%E5%AD%A6%E5%8A%A8%E7%89%A9%E5%8C%BB%E5%AD%A6%E9%99%A24%E4%BE%8B%E7%9C%9F%E8%8F%8C%E8%BD%AC%E5%BD%95%E7%BB%84%E6%B5%8B%E5%BA%8F%E9%A1%B9%E7%9B%AE-Denovo-RNAseq_result\4_basic_annotation\KEGG\Trichophyton_mentagrophytes-Unigene.fa.htm#gene66) | 19 (0.83%) | ko00670 |
| 67 | [Pentose and glucuronate interconversions](file:///G:\WEN\%E8%BD%AC%E5%BD%95%E7%BB%84%E6%B5%8B%E5%BA%8F\%E3%80%90%E6%89%B9%E9%87%8F%E4%B8%8B%E8%BD%BD%E3%80%91GDR1624-%E8%A5%BF%E5%8C%97%E5%86%9C%E6%9E%97%E7%A7%91%E6%8A%80%E5%A4%A7%E5%AD%A6%E5%8A%A8%E7%89%A9%E5%8C%BB%E5%AD%A6%E9%99%A24%E4%BE%8B%E7%9C%9F%E8%8F%8C%E8%BD%AC%E5%BD%95%E7%BB%84%E6%B5%8B%E5%BA%8F%E9%A1%B9%E7%9B%AE-%E5%89%94%E9%99%A4T2%E6%A0%B7%E5%93%81-Denovo_%E5%AE%8C%E6%95%B4%E7%89%88%E7%BB%93%E9%A2%98%E6%8A%A5%E5%91%8A%E7%AD%89\GDR1624-%E8%A5%BF%E5%8C%97%E5%86%9C%E6%9E%97%E7%A7%91%E6%8A%80%E5%A4%A7%E5%AD%A6%E5%8A%A8%E7%89%A9%E5%8C%BB%E5%AD%A6%E9%99%A24%E4%BE%8B%E7%9C%9F%E8%8F%8C%E8%BD%AC%E5%BD%95%E7%BB%84%E6%B5%8B%E5%BA%8F%E9%A1%B9%E7%9B%AE-Denovo-RNAseq_result\4_basic_annotation\KEGG\Trichophyton_mentagrophytes-Unigene.fa.htm#gene67) | 19 (0.83%) | ko00040 |
| 68 | [Methane metabolism](file:///G:\WEN\%E8%BD%AC%E5%BD%95%E7%BB%84%E6%B5%8B%E5%BA%8F\%E3%80%90%E6%89%B9%E9%87%8F%E4%B8%8B%E8%BD%BD%E3%80%91GDR1624-%E8%A5%BF%E5%8C%97%E5%86%9C%E6%9E%97%E7%A7%91%E6%8A%80%E5%A4%A7%E5%AD%A6%E5%8A%A8%E7%89%A9%E5%8C%BB%E5%AD%A6%E9%99%A24%E4%BE%8B%E7%9C%9F%E8%8F%8C%E8%BD%AC%E5%BD%95%E7%BB%84%E6%B5%8B%E5%BA%8F%E9%A1%B9%E7%9B%AE-%E5%89%94%E9%99%A4T2%E6%A0%B7%E5%93%81-Denovo_%E5%AE%8C%E6%95%B4%E7%89%88%E7%BB%93%E9%A2%98%E6%8A%A5%E5%91%8A%E7%AD%89\GDR1624-%E8%A5%BF%E5%8C%97%E5%86%9C%E6%9E%97%E7%A7%91%E6%8A%80%E5%A4%A7%E5%AD%A6%E5%8A%A8%E7%89%A9%E5%8C%BB%E5%AD%A6%E9%99%A24%E4%BE%8B%E7%9C%9F%E8%8F%8C%E8%BD%AC%E5%BD%95%E7%BB%84%E6%B5%8B%E5%BA%8F%E9%A1%B9%E7%9B%AE-Denovo-RNAseq_result\4_basic_annotation\KEGG\Trichophyton_mentagrophytes-Unigene.fa.htm#gene68) | 19 (0.83%) | ko00680 |
| 69 | [Galactose metabolism](file:///G:\WEN\%E8%BD%AC%E5%BD%95%E7%BB%84%E6%B5%8B%E5%BA%8F\%E3%80%90%E6%89%B9%E9%87%8F%E4%B8%8B%E8%BD%BD%E3%80%91GDR1624-%E8%A5%BF%E5%8C%97%E5%86%9C%E6%9E%97%E7%A7%91%E6%8A%80%E5%A4%A7%E5%AD%A6%E5%8A%A8%E7%89%A9%E5%8C%BB%E5%AD%A6%E9%99%A24%E4%BE%8B%E7%9C%9F%E8%8F%8C%E8%BD%AC%E5%BD%95%E7%BB%84%E6%B5%8B%E5%BA%8F%E9%A1%B9%E7%9B%AE-%E5%89%94%E9%99%A4T2%E6%A0%B7%E5%93%81-Denovo_%E5%AE%8C%E6%95%B4%E7%89%88%E7%BB%93%E9%A2%98%E6%8A%A5%E5%91%8A%E7%AD%89\GDR1624-%E8%A5%BF%E5%8C%97%E5%86%9C%E6%9E%97%E7%A7%91%E6%8A%80%E5%A4%A7%E5%AD%A6%E5%8A%A8%E7%89%A9%E5%8C%BB%E5%AD%A6%E9%99%A24%E4%BE%8B%E7%9C%9F%E8%8F%8C%E8%BD%AC%E5%BD%95%E7%BB%84%E6%B5%8B%E5%BA%8F%E9%A1%B9%E7%9B%AE-Denovo-RNAseq_result\4_basic_annotation\KEGG\Trichophyton_mentagrophytes-Unigene.fa.htm#gene69) | 19 (0.83%) | ko00052 |
| 70 | [Porphyrin and chlorophyll metabolism](file:///G:\WEN\%E8%BD%AC%E5%BD%95%E7%BB%84%E6%B5%8B%E5%BA%8F\%E3%80%90%E6%89%B9%E9%87%8F%E4%B8%8B%E8%BD%BD%E3%80%91GDR1624-%E8%A5%BF%E5%8C%97%E5%86%9C%E6%9E%97%E7%A7%91%E6%8A%80%E5%A4%A7%E5%AD%A6%E5%8A%A8%E7%89%A9%E5%8C%BB%E5%AD%A6%E9%99%A24%E4%BE%8B%E7%9C%9F%E8%8F%8C%E8%BD%AC%E5%BD%95%E7%BB%84%E6%B5%8B%E5%BA%8F%E9%A1%B9%E7%9B%AE-%E5%89%94%E9%99%A4T2%E6%A0%B7%E5%93%81-Denovo_%E5%AE%8C%E6%95%B4%E7%89%88%E7%BB%93%E9%A2%98%E6%8A%A5%E5%91%8A%E7%AD%89\GDR1624-%E8%A5%BF%E5%8C%97%E5%86%9C%E6%9E%97%E7%A7%91%E6%8A%80%E5%A4%A7%E5%AD%A6%E5%8A%A8%E7%89%A9%E5%8C%BB%E5%AD%A6%E9%99%A24%E4%BE%8B%E7%9C%9F%E8%8F%8C%E8%BD%AC%E5%BD%95%E7%BB%84%E6%B5%8B%E5%BA%8F%E9%A1%B9%E7%9B%AE-Denovo-RNAseq_result\4_basic_annotation\KEGG\Trichophyton_mentagrophytes-Unigene.fa.htm#gene70) | 19 (0.83%) | ko00860 |
| 71 | [Ether lipid metabolism](file:///G:\WEN\%E8%BD%AC%E5%BD%95%E7%BB%84%E6%B5%8B%E5%BA%8F\%E3%80%90%E6%89%B9%E9%87%8F%E4%B8%8B%E8%BD%BD%E3%80%91GDR1624-%E8%A5%BF%E5%8C%97%E5%86%9C%E6%9E%97%E7%A7%91%E6%8A%80%E5%A4%A7%E5%AD%A6%E5%8A%A8%E7%89%A9%E5%8C%BB%E5%AD%A6%E9%99%A24%E4%BE%8B%E7%9C%9F%E8%8F%8C%E8%BD%AC%E5%BD%95%E7%BB%84%E6%B5%8B%E5%BA%8F%E9%A1%B9%E7%9B%AE-%E5%89%94%E9%99%A4T2%E6%A0%B7%E5%93%81-Denovo_%E5%AE%8C%E6%95%B4%E7%89%88%E7%BB%93%E9%A2%98%E6%8A%A5%E5%91%8A%E7%AD%89\GDR1624-%E8%A5%BF%E5%8C%97%E5%86%9C%E6%9E%97%E7%A7%91%E6%8A%80%E5%A4%A7%E5%AD%A6%E5%8A%A8%E7%89%A9%E5%8C%BB%E5%AD%A6%E9%99%A24%E4%BE%8B%E7%9C%9F%E8%8F%8C%E8%BD%AC%E5%BD%95%E7%BB%84%E6%B5%8B%E5%BA%8F%E9%A1%B9%E7%9B%AE-Denovo-RNAseq_result\4_basic_annotation\KEGG\Trichophyton_mentagrophytes-Unigene.fa.htm#gene71) | 17 (0.74%) | ko00565 |
| 72 | [Folate biosynthesis](file:///G:\WEN\%E8%BD%AC%E5%BD%95%E7%BB%84%E6%B5%8B%E5%BA%8F\%E3%80%90%E6%89%B9%E9%87%8F%E4%B8%8B%E8%BD%BD%E3%80%91GDR1624-%E8%A5%BF%E5%8C%97%E5%86%9C%E6%9E%97%E7%A7%91%E6%8A%80%E5%A4%A7%E5%AD%A6%E5%8A%A8%E7%89%A9%E5%8C%BB%E5%AD%A6%E9%99%A24%E4%BE%8B%E7%9C%9F%E8%8F%8C%E8%BD%AC%E5%BD%95%E7%BB%84%E6%B5%8B%E5%BA%8F%E9%A1%B9%E7%9B%AE-%E5%89%94%E9%99%A4T2%E6%A0%B7%E5%93%81-Denovo_%E5%AE%8C%E6%95%B4%E7%89%88%E7%BB%93%E9%A2%98%E6%8A%A5%E5%91%8A%E7%AD%89\GDR1624-%E8%A5%BF%E5%8C%97%E5%86%9C%E6%9E%97%E7%A7%91%E6%8A%80%E5%A4%A7%E5%AD%A6%E5%8A%A8%E7%89%A9%E5%8C%BB%E5%AD%A6%E9%99%A24%E4%BE%8B%E7%9C%9F%E8%8F%8C%E8%BD%AC%E5%BD%95%E7%BB%84%E6%B5%8B%E5%BA%8F%E9%A1%B9%E7%9B%AE-Denovo-RNAseq_result\4_basic_annotation\KEGG\Trichophyton_mentagrophytes-Unigene.fa.htm#gene72) | 16 (0.7%) | ko00790 |
| 73 | [SNARE interactions in vesicular transport](file:///G:\WEN\%E8%BD%AC%E5%BD%95%E7%BB%84%E6%B5%8B%E5%BA%8F\%E3%80%90%E6%89%B9%E9%87%8F%E4%B8%8B%E8%BD%BD%E3%80%91GDR1624-%E8%A5%BF%E5%8C%97%E5%86%9C%E6%9E%97%E7%A7%91%E6%8A%80%E5%A4%A7%E5%AD%A6%E5%8A%A8%E7%89%A9%E5%8C%BB%E5%AD%A6%E9%99%A24%E4%BE%8B%E7%9C%9F%E8%8F%8C%E8%BD%AC%E5%BD%95%E7%BB%84%E6%B5%8B%E5%BA%8F%E9%A1%B9%E7%9B%AE-%E5%89%94%E9%99%A4T2%E6%A0%B7%E5%93%81-Denovo_%E5%AE%8C%E6%95%B4%E7%89%88%E7%BB%93%E9%A2%98%E6%8A%A5%E5%91%8A%E7%AD%89\GDR1624-%E8%A5%BF%E5%8C%97%E5%86%9C%E6%9E%97%E7%A7%91%E6%8A%80%E5%A4%A7%E5%AD%A6%E5%8A%A8%E7%89%A9%E5%8C%BB%E5%AD%A6%E9%99%A24%E4%BE%8B%E7%9C%9F%E8%8F%8C%E8%BD%AC%E5%BD%95%E7%BB%84%E6%B5%8B%E5%BA%8F%E9%A1%B9%E7%9B%AE-Denovo-RNAseq_result\4_basic_annotation\KEGG\Trichophyton_mentagrophytes-Unigene.fa.htm#gene73) | 16 (0.7%) | ko04130 |
| 74 | [Regulation of autophagy](file:///G:\WEN\%E8%BD%AC%E5%BD%95%E7%BB%84%E6%B5%8B%E5%BA%8F\%E3%80%90%E6%89%B9%E9%87%8F%E4%B8%8B%E8%BD%BD%E3%80%91GDR1624-%E8%A5%BF%E5%8C%97%E5%86%9C%E6%9E%97%E7%A7%91%E6%8A%80%E5%A4%A7%E5%AD%A6%E5%8A%A8%E7%89%A9%E5%8C%BB%E5%AD%A6%E9%99%A24%E4%BE%8B%E7%9C%9F%E8%8F%8C%E8%BD%AC%E5%BD%95%E7%BB%84%E6%B5%8B%E5%BA%8F%E9%A1%B9%E7%9B%AE-%E5%89%94%E9%99%A4T2%E6%A0%B7%E5%93%81-Denovo_%E5%AE%8C%E6%95%B4%E7%89%88%E7%BB%93%E9%A2%98%E6%8A%A5%E5%91%8A%E7%AD%89\GDR1624-%E8%A5%BF%E5%8C%97%E5%86%9C%E6%9E%97%E7%A7%91%E6%8A%80%E5%A4%A7%E5%AD%A6%E5%8A%A8%E7%89%A9%E5%8C%BB%E5%AD%A6%E9%99%A24%E4%BE%8B%E7%9C%9F%E8%8F%8C%E8%BD%AC%E5%BD%95%E7%BB%84%E6%B5%8B%E5%BA%8F%E9%A1%B9%E7%9B%AE-Denovo-RNAseq_result\4_basic_annotation\KEGG\Trichophyton_mentagrophytes-Unigene.fa.htm#gene74) | 16 (0.7%) | ko04140 |
| 75 | [Protein export](file:///G:\WEN\%E8%BD%AC%E5%BD%95%E7%BB%84%E6%B5%8B%E5%BA%8F\%E3%80%90%E6%89%B9%E9%87%8F%E4%B8%8B%E8%BD%BD%E3%80%91GDR1624-%E8%A5%BF%E5%8C%97%E5%86%9C%E6%9E%97%E7%A7%91%E6%8A%80%E5%A4%A7%E5%AD%A6%E5%8A%A8%E7%89%A9%E5%8C%BB%E5%AD%A6%E9%99%A24%E4%BE%8B%E7%9C%9F%E8%8F%8C%E8%BD%AC%E5%BD%95%E7%BB%84%E6%B5%8B%E5%BA%8F%E9%A1%B9%E7%9B%AE-%E5%89%94%E9%99%A4T2%E6%A0%B7%E5%93%81-Denovo_%E5%AE%8C%E6%95%B4%E7%89%88%E7%BB%93%E9%A2%98%E6%8A%A5%E5%91%8A%E7%AD%89\GDR1624-%E8%A5%BF%E5%8C%97%E5%86%9C%E6%9E%97%E7%A7%91%E6%8A%80%E5%A4%A7%E5%AD%A6%E5%8A%A8%E7%89%A9%E5%8C%BB%E5%AD%A6%E9%99%A24%E4%BE%8B%E7%9C%9F%E8%8F%8C%E8%BD%AC%E5%BD%95%E7%BB%84%E6%B5%8B%E5%BA%8F%E9%A1%B9%E7%9B%AE-Denovo-RNAseq_result\4_basic_annotation\KEGG\Trichophyton_mentagrophytes-Unigene.fa.htm#gene75) | 16 (0.7%) | ko03060 |
| 76 | [Cyanoamino acid metabolism](file:///G:\WEN\%E8%BD%AC%E5%BD%95%E7%BB%84%E6%B5%8B%E5%BA%8F\%E3%80%90%E6%89%B9%E9%87%8F%E4%B8%8B%E8%BD%BD%E3%80%91GDR1624-%E8%A5%BF%E5%8C%97%E5%86%9C%E6%9E%97%E7%A7%91%E6%8A%80%E5%A4%A7%E5%AD%A6%E5%8A%A8%E7%89%A9%E5%8C%BB%E5%AD%A6%E9%99%A24%E4%BE%8B%E7%9C%9F%E8%8F%8C%E8%BD%AC%E5%BD%95%E7%BB%84%E6%B5%8B%E5%BA%8F%E9%A1%B9%E7%9B%AE-%E5%89%94%E9%99%A4T2%E6%A0%B7%E5%93%81-Denovo_%E5%AE%8C%E6%95%B4%E7%89%88%E7%BB%93%E9%A2%98%E6%8A%A5%E5%91%8A%E7%AD%89\GDR1624-%E8%A5%BF%E5%8C%97%E5%86%9C%E6%9E%97%E7%A7%91%E6%8A%80%E5%A4%A7%E5%AD%A6%E5%8A%A8%E7%89%A9%E5%8C%BB%E5%AD%A6%E9%99%A24%E4%BE%8B%E7%9C%9F%E8%8F%8C%E8%BD%AC%E5%BD%95%E7%BB%84%E6%B5%8B%E5%BA%8F%E9%A1%B9%E7%9B%AE-Denovo-RNAseq_result\4_basic_annotation\KEGG\Trichophyton_mentagrophytes-Unigene.fa.htm#gene76) | 16 (0.7%) | ko00460 |
| 77 | [Nitrogen metabolism](file:///G:\WEN\%E8%BD%AC%E5%BD%95%E7%BB%84%E6%B5%8B%E5%BA%8F\%E3%80%90%E6%89%B9%E9%87%8F%E4%B8%8B%E8%BD%BD%E3%80%91GDR1624-%E8%A5%BF%E5%8C%97%E5%86%9C%E6%9E%97%E7%A7%91%E6%8A%80%E5%A4%A7%E5%AD%A6%E5%8A%A8%E7%89%A9%E5%8C%BB%E5%AD%A6%E9%99%A24%E4%BE%8B%E7%9C%9F%E8%8F%8C%E8%BD%AC%E5%BD%95%E7%BB%84%E6%B5%8B%E5%BA%8F%E9%A1%B9%E7%9B%AE-%E5%89%94%E9%99%A4T2%E6%A0%B7%E5%93%81-Denovo_%E5%AE%8C%E6%95%B4%E7%89%88%E7%BB%93%E9%A2%98%E6%8A%A5%E5%91%8A%E7%AD%89\GDR1624-%E8%A5%BF%E5%8C%97%E5%86%9C%E6%9E%97%E7%A7%91%E6%8A%80%E5%A4%A7%E5%AD%A6%E5%8A%A8%E7%89%A9%E5%8C%BB%E5%AD%A6%E9%99%A24%E4%BE%8B%E7%9C%9F%E8%8F%8C%E8%BD%AC%E5%BD%95%E7%BB%84%E6%B5%8B%E5%BA%8F%E9%A1%B9%E7%9B%AE-Denovo-RNAseq_result\4_basic_annotation\KEGG\Trichophyton_mentagrophytes-Unigene.fa.htm#gene77) | 15 (0.65%) | ko00910 |
| 78 | [Nicotinate and nicotinamide metabolism](file:///G:\WEN\%E8%BD%AC%E5%BD%95%E7%BB%84%E6%B5%8B%E5%BA%8F\%E3%80%90%E6%89%B9%E9%87%8F%E4%B8%8B%E8%BD%BD%E3%80%91GDR1624-%E8%A5%BF%E5%8C%97%E5%86%9C%E6%9E%97%E7%A7%91%E6%8A%80%E5%A4%A7%E5%AD%A6%E5%8A%A8%E7%89%A9%E5%8C%BB%E5%AD%A6%E9%99%A24%E4%BE%8B%E7%9C%9F%E8%8F%8C%E8%BD%AC%E5%BD%95%E7%BB%84%E6%B5%8B%E5%BA%8F%E9%A1%B9%E7%9B%AE-%E5%89%94%E9%99%A4T2%E6%A0%B7%E5%93%81-Denovo_%E5%AE%8C%E6%95%B4%E7%89%88%E7%BB%93%E9%A2%98%E6%8A%A5%E5%91%8A%E7%AD%89\GDR1624-%E8%A5%BF%E5%8C%97%E5%86%9C%E6%9E%97%E7%A7%91%E6%8A%80%E5%A4%A7%E5%AD%A6%E5%8A%A8%E7%89%A9%E5%8C%BB%E5%AD%A6%E9%99%A24%E4%BE%8B%E7%9C%9F%E8%8F%8C%E8%BD%AC%E5%BD%95%E7%BB%84%E6%B5%8B%E5%BA%8F%E9%A1%B9%E7%9B%AE-Denovo-RNAseq_result\4_basic_annotation\KEGG\Trichophyton_mentagrophytes-Unigene.fa.htm#gene78) | 15 (0.65%) | ko00760 |
| 79 | [Histidine metabolism](file:///G:\WEN\%E8%BD%AC%E5%BD%95%E7%BB%84%E6%B5%8B%E5%BA%8F\%E3%80%90%E6%89%B9%E9%87%8F%E4%B8%8B%E8%BD%BD%E3%80%91GDR1624-%E8%A5%BF%E5%8C%97%E5%86%9C%E6%9E%97%E7%A7%91%E6%8A%80%E5%A4%A7%E5%AD%A6%E5%8A%A8%E7%89%A9%E5%8C%BB%E5%AD%A6%E9%99%A24%E4%BE%8B%E7%9C%9F%E8%8F%8C%E8%BD%AC%E5%BD%95%E7%BB%84%E6%B5%8B%E5%BA%8F%E9%A1%B9%E7%9B%AE-%E5%89%94%E9%99%A4T2%E6%A0%B7%E5%93%81-Denovo_%E5%AE%8C%E6%95%B4%E7%89%88%E7%BB%93%E9%A2%98%E6%8A%A5%E5%91%8A%E7%AD%89\GDR1624-%E8%A5%BF%E5%8C%97%E5%86%9C%E6%9E%97%E7%A7%91%E6%8A%80%E5%A4%A7%E5%AD%A6%E5%8A%A8%E7%89%A9%E5%8C%BB%E5%AD%A6%E9%99%A24%E4%BE%8B%E7%9C%9F%E8%8F%8C%E8%BD%AC%E5%BD%95%E7%BB%84%E6%B5%8B%E5%BA%8F%E9%A1%B9%E7%9B%AE-Denovo-RNAseq_result\4_basic_annotation\KEGG\Trichophyton_mentagrophytes-Unigene.fa.htm#gene79) | 15 (0.65%) | ko00340 |
| 80 | [Inositol phosphate metabolism](file:///G:\WEN\%E8%BD%AC%E5%BD%95%E7%BB%84%E6%B5%8B%E5%BA%8F\%E3%80%90%E6%89%B9%E9%87%8F%E4%B8%8B%E8%BD%BD%E3%80%91GDR1624-%E8%A5%BF%E5%8C%97%E5%86%9C%E6%9E%97%E7%A7%91%E6%8A%80%E5%A4%A7%E5%AD%A6%E5%8A%A8%E7%89%A9%E5%8C%BB%E5%AD%A6%E9%99%A24%E4%BE%8B%E7%9C%9F%E8%8F%8C%E8%BD%AC%E5%BD%95%E7%BB%84%E6%B5%8B%E5%BA%8F%E9%A1%B9%E7%9B%AE-%E5%89%94%E9%99%A4T2%E6%A0%B7%E5%93%81-Denovo_%E5%AE%8C%E6%95%B4%E7%89%88%E7%BB%93%E9%A2%98%E6%8A%A5%E5%91%8A%E7%AD%89\GDR1624-%E8%A5%BF%E5%8C%97%E5%86%9C%E6%9E%97%E7%A7%91%E6%8A%80%E5%A4%A7%E5%AD%A6%E5%8A%A8%E7%89%A9%E5%8C%BB%E5%AD%A6%E9%99%A24%E4%BE%8B%E7%9C%9F%E8%8F%8C%E8%BD%AC%E5%BD%95%E7%BB%84%E6%B5%8B%E5%BA%8F%E9%A1%B9%E7%9B%AE-Denovo-RNAseq_result\4_basic_annotation\KEGG\Trichophyton_mentagrophytes-Unigene.fa.htm#gene80) | 14 (0.61%) | ko00562 |
| 81 | [Ubiquinone and other terpenoid-quinone biosynthesis](file:///G:\WEN\%E8%BD%AC%E5%BD%95%E7%BB%84%E6%B5%8B%E5%BA%8F\%E3%80%90%E6%89%B9%E9%87%8F%E4%B8%8B%E8%BD%BD%E3%80%91GDR1624-%E8%A5%BF%E5%8C%97%E5%86%9C%E6%9E%97%E7%A7%91%E6%8A%80%E5%A4%A7%E5%AD%A6%E5%8A%A8%E7%89%A9%E5%8C%BB%E5%AD%A6%E9%99%A24%E4%BE%8B%E7%9C%9F%E8%8F%8C%E8%BD%AC%E5%BD%95%E7%BB%84%E6%B5%8B%E5%BA%8F%E9%A1%B9%E7%9B%AE-%E5%89%94%E9%99%A4T2%E6%A0%B7%E5%93%81-Denovo_%E5%AE%8C%E6%95%B4%E7%89%88%E7%BB%93%E9%A2%98%E6%8A%A5%E5%91%8A%E7%AD%89\GDR1624-%E8%A5%BF%E5%8C%97%E5%86%9C%E6%9E%97%E7%A7%91%E6%8A%80%E5%A4%A7%E5%AD%A6%E5%8A%A8%E7%89%A9%E5%8C%BB%E5%AD%A6%E9%99%A24%E4%BE%8B%E7%9C%9F%E8%8F%8C%E8%BD%AC%E5%BD%95%E7%BB%84%E6%B5%8B%E5%BA%8F%E9%A1%B9%E7%9B%AE-Denovo-RNAseq_result\4_basic_annotation\KEGG\Trichophyton_mentagrophytes-Unigene.fa.htm#gene81) | 14 (0.61%) | ko00130 |
| 82 | [Lysine biosynthesis](file:///G:\WEN\%E8%BD%AC%E5%BD%95%E7%BB%84%E6%B5%8B%E5%BA%8F\%E3%80%90%E6%89%B9%E9%87%8F%E4%B8%8B%E8%BD%BD%E3%80%91GDR1624-%E8%A5%BF%E5%8C%97%E5%86%9C%E6%9E%97%E7%A7%91%E6%8A%80%E5%A4%A7%E5%AD%A6%E5%8A%A8%E7%89%A9%E5%8C%BB%E5%AD%A6%E9%99%A24%E4%BE%8B%E7%9C%9F%E8%8F%8C%E8%BD%AC%E5%BD%95%E7%BB%84%E6%B5%8B%E5%BA%8F%E9%A1%B9%E7%9B%AE-%E5%89%94%E9%99%A4T2%E6%A0%B7%E5%93%81-Denovo_%E5%AE%8C%E6%95%B4%E7%89%88%E7%BB%93%E9%A2%98%E6%8A%A5%E5%91%8A%E7%AD%89\GDR1624-%E8%A5%BF%E5%8C%97%E5%86%9C%E6%9E%97%E7%A7%91%E6%8A%80%E5%A4%A7%E5%AD%A6%E5%8A%A8%E7%89%A9%E5%8C%BB%E5%AD%A6%E9%99%A24%E4%BE%8B%E7%9C%9F%E8%8F%8C%E8%BD%AC%E5%BD%95%E7%BB%84%E6%B5%8B%E5%BA%8F%E9%A1%B9%E7%9B%AE-Denovo-RNAseq_result\4_basic_annotation\KEGG\Trichophyton_mentagrophytes-Unigene.fa.htm#gene82) | 14 (0.61%) | ko00300 |
| 83 | [Biosynthesis of unsaturated fatty acids](file:///G:\WEN\%E8%BD%AC%E5%BD%95%E7%BB%84%E6%B5%8B%E5%BA%8F\%E3%80%90%E6%89%B9%E9%87%8F%E4%B8%8B%E8%BD%BD%E3%80%91GDR1624-%E8%A5%BF%E5%8C%97%E5%86%9C%E6%9E%97%E7%A7%91%E6%8A%80%E5%A4%A7%E5%AD%A6%E5%8A%A8%E7%89%A9%E5%8C%BB%E5%AD%A6%E9%99%A24%E4%BE%8B%E7%9C%9F%E8%8F%8C%E8%BD%AC%E5%BD%95%E7%BB%84%E6%B5%8B%E5%BA%8F%E9%A1%B9%E7%9B%AE-%E5%89%94%E9%99%A4T2%E6%A0%B7%E5%93%81-Denovo_%E5%AE%8C%E6%95%B4%E7%89%88%E7%BB%93%E9%A2%98%E6%8A%A5%E5%91%8A%E7%AD%89\GDR1624-%E8%A5%BF%E5%8C%97%E5%86%9C%E6%9E%97%E7%A7%91%E6%8A%80%E5%A4%A7%E5%AD%A6%E5%8A%A8%E7%89%A9%E5%8C%BB%E5%AD%A6%E9%99%A24%E4%BE%8B%E7%9C%9F%E8%8F%8C%E8%BD%AC%E5%BD%95%E7%BB%84%E6%B5%8B%E5%BA%8F%E9%A1%B9%E7%9B%AE-Denovo-RNAseq_result\4_basic_annotation\KEGG\Trichophyton_mentagrophytes-Unigene.fa.htm#gene83) | 14 (0.61%) | ko01040 |
| 84 | [ABC transporters](file:///G:\WEN\%E8%BD%AC%E5%BD%95%E7%BB%84%E6%B5%8B%E5%BA%8F\%E3%80%90%E6%89%B9%E9%87%8F%E4%B8%8B%E8%BD%BD%E3%80%91GDR1624-%E8%A5%BF%E5%8C%97%E5%86%9C%E6%9E%97%E7%A7%91%E6%8A%80%E5%A4%A7%E5%AD%A6%E5%8A%A8%E7%89%A9%E5%8C%BB%E5%AD%A6%E9%99%A24%E4%BE%8B%E7%9C%9F%E8%8F%8C%E8%BD%AC%E5%BD%95%E7%BB%84%E6%B5%8B%E5%BA%8F%E9%A1%B9%E7%9B%AE-%E5%89%94%E9%99%A4T2%E6%A0%B7%E5%93%81-Denovo_%E5%AE%8C%E6%95%B4%E7%89%88%E7%BB%93%E9%A2%98%E6%8A%A5%E5%91%8A%E7%AD%89\GDR1624-%E8%A5%BF%E5%8C%97%E5%86%9C%E6%9E%97%E7%A7%91%E6%8A%80%E5%A4%A7%E5%AD%A6%E5%8A%A8%E7%89%A9%E5%8C%BB%E5%AD%A6%E9%99%A24%E4%BE%8B%E7%9C%9F%E8%8F%8C%E8%BD%AC%E5%BD%95%E7%BB%84%E6%B5%8B%E5%BA%8F%E9%A1%B9%E7%9B%AE-Denovo-RNAseq_result\4_basic_annotation\KEGG\Trichophyton_mentagrophytes-Unigene.fa.htm#gene84) | 13 (0.57%) | ko02010 |
| 85 | [Phosphatidylinositol signaling system](file:///G:\WEN\%E8%BD%AC%E5%BD%95%E7%BB%84%E6%B5%8B%E5%BA%8F\%E3%80%90%E6%89%B9%E9%87%8F%E4%B8%8B%E8%BD%BD%E3%80%91GDR1624-%E8%A5%BF%E5%8C%97%E5%86%9C%E6%9E%97%E7%A7%91%E6%8A%80%E5%A4%A7%E5%AD%A6%E5%8A%A8%E7%89%A9%E5%8C%BB%E5%AD%A6%E9%99%A24%E4%BE%8B%E7%9C%9F%E8%8F%8C%E8%BD%AC%E5%BD%95%E7%BB%84%E6%B5%8B%E5%BA%8F%E9%A1%B9%E7%9B%AE-%E5%89%94%E9%99%A4T2%E6%A0%B7%E5%93%81-Denovo_%E5%AE%8C%E6%95%B4%E7%89%88%E7%BB%93%E9%A2%98%E6%8A%A5%E5%91%8A%E7%AD%89\GDR1624-%E8%A5%BF%E5%8C%97%E5%86%9C%E6%9E%97%E7%A7%91%E6%8A%80%E5%A4%A7%E5%AD%A6%E5%8A%A8%E7%89%A9%E5%8C%BB%E5%AD%A6%E9%99%A24%E4%BE%8B%E7%9C%9F%E8%8F%8C%E8%BD%AC%E5%BD%95%E7%BB%84%E6%B5%8B%E5%BA%8F%E9%A1%B9%E7%9B%AE-Denovo-RNAseq_result\4_basic_annotation\KEGG\Trichophyton_mentagrophytes-Unigene.fa.htm#gene85) | 12 (0.52%) | ko04070 |
| 86 | [Biotin metabolism](file:///G:\WEN\%E8%BD%AC%E5%BD%95%E7%BB%84%E6%B5%8B%E5%BA%8F\%E3%80%90%E6%89%B9%E9%87%8F%E4%B8%8B%E8%BD%BD%E3%80%91GDR1624-%E8%A5%BF%E5%8C%97%E5%86%9C%E6%9E%97%E7%A7%91%E6%8A%80%E5%A4%A7%E5%AD%A6%E5%8A%A8%E7%89%A9%E5%8C%BB%E5%AD%A6%E9%99%A24%E4%BE%8B%E7%9C%9F%E8%8F%8C%E8%BD%AC%E5%BD%95%E7%BB%84%E6%B5%8B%E5%BA%8F%E9%A1%B9%E7%9B%AE-%E5%89%94%E9%99%A4T2%E6%A0%B7%E5%93%81-Denovo_%E5%AE%8C%E6%95%B4%E7%89%88%E7%BB%93%E9%A2%98%E6%8A%A5%E5%91%8A%E7%AD%89\GDR1624-%E8%A5%BF%E5%8C%97%E5%86%9C%E6%9E%97%E7%A7%91%E6%8A%80%E5%A4%A7%E5%AD%A6%E5%8A%A8%E7%89%A9%E5%8C%BB%E5%AD%A6%E9%99%A24%E4%BE%8B%E7%9C%9F%E8%8F%8C%E8%BD%AC%E5%BD%95%E7%BB%84%E6%B5%8B%E5%BA%8F%E9%A1%B9%E7%9B%AE-Denovo-RNAseq_result\4_basic_annotation\KEGG\Trichophyton_mentagrophytes-Unigene.fa.htm#gene86) | 12 (0.52%) | ko00780 |
| 87 | [Non-homologous end-joining](file:///G:\WEN\%E8%BD%AC%E5%BD%95%E7%BB%84%E6%B5%8B%E5%BA%8F\%E3%80%90%E6%89%B9%E9%87%8F%E4%B8%8B%E8%BD%BD%E3%80%91GDR1624-%E8%A5%BF%E5%8C%97%E5%86%9C%E6%9E%97%E7%A7%91%E6%8A%80%E5%A4%A7%E5%AD%A6%E5%8A%A8%E7%89%A9%E5%8C%BB%E5%AD%A6%E9%99%A24%E4%BE%8B%E7%9C%9F%E8%8F%8C%E8%BD%AC%E5%BD%95%E7%BB%84%E6%B5%8B%E5%BA%8F%E9%A1%B9%E7%9B%AE-%E5%89%94%E9%99%A4T2%E6%A0%B7%E5%93%81-Denovo_%E5%AE%8C%E6%95%B4%E7%89%88%E7%BB%93%E9%A2%98%E6%8A%A5%E5%91%8A%E7%AD%89\GDR1624-%E8%A5%BF%E5%8C%97%E5%86%9C%E6%9E%97%E7%A7%91%E6%8A%80%E5%A4%A7%E5%AD%A6%E5%8A%A8%E7%89%A9%E5%8C%BB%E5%AD%A6%E9%99%A24%E4%BE%8B%E7%9C%9F%E8%8F%8C%E8%BD%AC%E5%BD%95%E7%BB%84%E6%B5%8B%E5%BA%8F%E9%A1%B9%E7%9B%AE-Denovo-RNAseq_result\4_basic_annotation\KEGG\Trichophyton_mentagrophytes-Unigene.fa.htm#gene87) | 12 (0.52%) | ko03450 |
| 88 | [Sulfur relay system](file:///G:\WEN\%E8%BD%AC%E5%BD%95%E7%BB%84%E6%B5%8B%E5%BA%8F\%E3%80%90%E6%89%B9%E9%87%8F%E4%B8%8B%E8%BD%BD%E3%80%91GDR1624-%E8%A5%BF%E5%8C%97%E5%86%9C%E6%9E%97%E7%A7%91%E6%8A%80%E5%A4%A7%E5%AD%A6%E5%8A%A8%E7%89%A9%E5%8C%BB%E5%AD%A6%E9%99%A24%E4%BE%8B%E7%9C%9F%E8%8F%8C%E8%BD%AC%E5%BD%95%E7%BB%84%E6%B5%8B%E5%BA%8F%E9%A1%B9%E7%9B%AE-%E5%89%94%E9%99%A4T2%E6%A0%B7%E5%93%81-Denovo_%E5%AE%8C%E6%95%B4%E7%89%88%E7%BB%93%E9%A2%98%E6%8A%A5%E5%91%8A%E7%AD%89\GDR1624-%E8%A5%BF%E5%8C%97%E5%86%9C%E6%9E%97%E7%A7%91%E6%8A%80%E5%A4%A7%E5%AD%A6%E5%8A%A8%E7%89%A9%E5%8C%BB%E5%AD%A6%E9%99%A24%E4%BE%8B%E7%9C%9F%E8%8F%8C%E8%BD%AC%E5%BD%95%E7%BB%84%E6%B5%8B%E5%BA%8F%E9%A1%B9%E7%9B%AE-Denovo-RNAseq_result\4_basic_annotation\KEGG\Trichophyton_mentagrophytes-Unigene.fa.htm#gene88) | 11 (0.48%) | ko04122 |
| 89 | [Linoleic acid metabolism](file:///G:\WEN\%E8%BD%AC%E5%BD%95%E7%BB%84%E6%B5%8B%E5%BA%8F\%E3%80%90%E6%89%B9%E9%87%8F%E4%B8%8B%E8%BD%BD%E3%80%91GDR1624-%E8%A5%BF%E5%8C%97%E5%86%9C%E6%9E%97%E7%A7%91%E6%8A%80%E5%A4%A7%E5%AD%A6%E5%8A%A8%E7%89%A9%E5%8C%BB%E5%AD%A6%E9%99%A24%E4%BE%8B%E7%9C%9F%E8%8F%8C%E8%BD%AC%E5%BD%95%E7%BB%84%E6%B5%8B%E5%BA%8F%E9%A1%B9%E7%9B%AE-%E5%89%94%E9%99%A4T2%E6%A0%B7%E5%93%81-Denovo_%E5%AE%8C%E6%95%B4%E7%89%88%E7%BB%93%E9%A2%98%E6%8A%A5%E5%91%8A%E7%AD%89\GDR1624-%E8%A5%BF%E5%8C%97%E5%86%9C%E6%9E%97%E7%A7%91%E6%8A%80%E5%A4%A7%E5%AD%A6%E5%8A%A8%E7%89%A9%E5%8C%BB%E5%AD%A6%E9%99%A24%E4%BE%8B%E7%9C%9F%E8%8F%8C%E8%BD%AC%E5%BD%95%E7%BB%84%E6%B5%8B%E5%BA%8F%E9%A1%B9%E7%9B%AE-Denovo-RNAseq_result\4_basic_annotation\KEGG\Trichophyton_mentagrophytes-Unigene.fa.htm#gene89) | 11 (0.48%) | ko00591 |
| 90 | [Sulfur metabolism](file:///G:\WEN\%E8%BD%AC%E5%BD%95%E7%BB%84%E6%B5%8B%E5%BA%8F\%E3%80%90%E6%89%B9%E9%87%8F%E4%B8%8B%E8%BD%BD%E3%80%91GDR1624-%E8%A5%BF%E5%8C%97%E5%86%9C%E6%9E%97%E7%A7%91%E6%8A%80%E5%A4%A7%E5%AD%A6%E5%8A%A8%E7%89%A9%E5%8C%BB%E5%AD%A6%E9%99%A24%E4%BE%8B%E7%9C%9F%E8%8F%8C%E8%BD%AC%E5%BD%95%E7%BB%84%E6%B5%8B%E5%BA%8F%E9%A1%B9%E7%9B%AE-%E5%89%94%E9%99%A4T2%E6%A0%B7%E5%93%81-Denovo_%E5%AE%8C%E6%95%B4%E7%89%88%E7%BB%93%E9%A2%98%E6%8A%A5%E5%91%8A%E7%AD%89\GDR1624-%E8%A5%BF%E5%8C%97%E5%86%9C%E6%9E%97%E7%A7%91%E6%8A%80%E5%A4%A7%E5%AD%A6%E5%8A%A8%E7%89%A9%E5%8C%BB%E5%AD%A6%E9%99%A24%E4%BE%8B%E7%9C%9F%E8%8F%8C%E8%BD%AC%E5%BD%95%E7%BB%84%E6%B5%8B%E5%BA%8F%E9%A1%B9%E7%9B%AE-Denovo-RNAseq_result\4_basic_annotation\KEGG\Trichophyton_mentagrophytes-Unigene.fa.htm#gene90) | 11 (0.48%) | ko00920 |
| 91 | [Selenocompound metabolism](file:///G:\WEN\%E8%BD%AC%E5%BD%95%E7%BB%84%E6%B5%8B%E5%BA%8F\%E3%80%90%E6%89%B9%E9%87%8F%E4%B8%8B%E8%BD%BD%E3%80%91GDR1624-%E8%A5%BF%E5%8C%97%E5%86%9C%E6%9E%97%E7%A7%91%E6%8A%80%E5%A4%A7%E5%AD%A6%E5%8A%A8%E7%89%A9%E5%8C%BB%E5%AD%A6%E9%99%A24%E4%BE%8B%E7%9C%9F%E8%8F%8C%E8%BD%AC%E5%BD%95%E7%BB%84%E6%B5%8B%E5%BA%8F%E9%A1%B9%E7%9B%AE-%E5%89%94%E9%99%A4T2%E6%A0%B7%E5%93%81-Denovo_%E5%AE%8C%E6%95%B4%E7%89%88%E7%BB%93%E9%A2%98%E6%8A%A5%E5%91%8A%E7%AD%89\GDR1624-%E8%A5%BF%E5%8C%97%E5%86%9C%E6%9E%97%E7%A7%91%E6%8A%80%E5%A4%A7%E5%AD%A6%E5%8A%A8%E7%89%A9%E5%8C%BB%E5%AD%A6%E9%99%A24%E4%BE%8B%E7%9C%9F%E8%8F%8C%E8%BD%AC%E5%BD%95%E7%BB%84%E6%B5%8B%E5%BA%8F%E9%A1%B9%E7%9B%AE-Denovo-RNAseq_result\4_basic_annotation\KEGG\Trichophyton_mentagrophytes-Unigene.fa.htm#gene91) | 11 (0.48%) | ko00450 |
| 92 | [Fatty acid biosynthesis](file:///G:\WEN\%E8%BD%AC%E5%BD%95%E7%BB%84%E6%B5%8B%E5%BA%8F\%E3%80%90%E6%89%B9%E9%87%8F%E4%B8%8B%E8%BD%BD%E3%80%91GDR1624-%E8%A5%BF%E5%8C%97%E5%86%9C%E6%9E%97%E7%A7%91%E6%8A%80%E5%A4%A7%E5%AD%A6%E5%8A%A8%E7%89%A9%E5%8C%BB%E5%AD%A6%E9%99%A24%E4%BE%8B%E7%9C%9F%E8%8F%8C%E8%BD%AC%E5%BD%95%E7%BB%84%E6%B5%8B%E5%BA%8F%E9%A1%B9%E7%9B%AE-%E5%89%94%E9%99%A4T2%E6%A0%B7%E5%93%81-Denovo_%E5%AE%8C%E6%95%B4%E7%89%88%E7%BB%93%E9%A2%98%E6%8A%A5%E5%91%8A%E7%AD%89\GDR1624-%E8%A5%BF%E5%8C%97%E5%86%9C%E6%9E%97%E7%A7%91%E6%8A%80%E5%A4%A7%E5%AD%A6%E5%8A%A8%E7%89%A9%E5%8C%BB%E5%AD%A6%E9%99%A24%E4%BE%8B%E7%9C%9F%E8%8F%8C%E8%BD%AC%E5%BD%95%E7%BB%84%E6%B5%8B%E5%BA%8F%E9%A1%B9%E7%9B%AE-Denovo-RNAseq_result\4_basic_annotation\KEGG\Trichophyton_mentagrophytes-Unigene.fa.htm#gene92) | 10 (0.44%) | ko00061 |
| 93 | [Taurine and hypotaurine metabolism](file:///G:\WEN\%E8%BD%AC%E5%BD%95%E7%BB%84%E6%B5%8B%E5%BA%8F\%E3%80%90%E6%89%B9%E9%87%8F%E4%B8%8B%E8%BD%BD%E3%80%91GDR1624-%E8%A5%BF%E5%8C%97%E5%86%9C%E6%9E%97%E7%A7%91%E6%8A%80%E5%A4%A7%E5%AD%A6%E5%8A%A8%E7%89%A9%E5%8C%BB%E5%AD%A6%E9%99%A24%E4%BE%8B%E7%9C%9F%E8%8F%8C%E8%BD%AC%E5%BD%95%E7%BB%84%E6%B5%8B%E5%BA%8F%E9%A1%B9%E7%9B%AE-%E5%89%94%E9%99%A4T2%E6%A0%B7%E5%93%81-Denovo_%E5%AE%8C%E6%95%B4%E7%89%88%E7%BB%93%E9%A2%98%E6%8A%A5%E5%91%8A%E7%AD%89\GDR1624-%E8%A5%BF%E5%8C%97%E5%86%9C%E6%9E%97%E7%A7%91%E6%8A%80%E5%A4%A7%E5%AD%A6%E5%8A%A8%E7%89%A9%E5%8C%BB%E5%AD%A6%E9%99%A24%E4%BE%8B%E7%9C%9F%E8%8F%8C%E8%BD%AC%E5%BD%95%E7%BB%84%E6%B5%8B%E5%BA%8F%E9%A1%B9%E7%9B%AE-Denovo-RNAseq_result\4_basic_annotation\KEGG\Trichophyton_mentagrophytes-Unigene.fa.htm#gene93) | 10 (0.44%) | ko00430 |
| 94 | [Glycosaminoglycan degradation](file:///G:\WEN\%E8%BD%AC%E5%BD%95%E7%BB%84%E6%B5%8B%E5%BA%8F\%E3%80%90%E6%89%B9%E9%87%8F%E4%B8%8B%E8%BD%BD%E3%80%91GDR1624-%E8%A5%BF%E5%8C%97%E5%86%9C%E6%9E%97%E7%A7%91%E6%8A%80%E5%A4%A7%E5%AD%A6%E5%8A%A8%E7%89%A9%E5%8C%BB%E5%AD%A6%E9%99%A24%E4%BE%8B%E7%9C%9F%E8%8F%8C%E8%BD%AC%E5%BD%95%E7%BB%84%E6%B5%8B%E5%BA%8F%E9%A1%B9%E7%9B%AE-%E5%89%94%E9%99%A4T2%E6%A0%B7%E5%93%81-Denovo_%E5%AE%8C%E6%95%B4%E7%89%88%E7%BB%93%E9%A2%98%E6%8A%A5%E5%91%8A%E7%AD%89\GDR1624-%E8%A5%BF%E5%8C%97%E5%86%9C%E6%9E%97%E7%A7%91%E6%8A%80%E5%A4%A7%E5%AD%A6%E5%8A%A8%E7%89%A9%E5%8C%BB%E5%AD%A6%E9%99%A24%E4%BE%8B%E7%9C%9F%E8%8F%8C%E8%BD%AC%E5%BD%95%E7%BB%84%E6%B5%8B%E5%BA%8F%E9%A1%B9%E7%9B%AE-Denovo-RNAseq_result\4_basic_annotation\KEGG\Trichophyton_mentagrophytes-Unigene.fa.htm#gene94) | 10 (0.44%) | ko00531 |
| 95 | [Thiamine metabolism](file:///G:\WEN\%E8%BD%AC%E5%BD%95%E7%BB%84%E6%B5%8B%E5%BA%8F\%E3%80%90%E6%89%B9%E9%87%8F%E4%B8%8B%E8%BD%BD%E3%80%91GDR1624-%E8%A5%BF%E5%8C%97%E5%86%9C%E6%9E%97%E7%A7%91%E6%8A%80%E5%A4%A7%E5%AD%A6%E5%8A%A8%E7%89%A9%E5%8C%BB%E5%AD%A6%E9%99%A24%E4%BE%8B%E7%9C%9F%E8%8F%8C%E8%BD%AC%E5%BD%95%E7%BB%84%E6%B5%8B%E5%BA%8F%E9%A1%B9%E7%9B%AE-%E5%89%94%E9%99%A4T2%E6%A0%B7%E5%93%81-Denovo_%E5%AE%8C%E6%95%B4%E7%89%88%E7%BB%93%E9%A2%98%E6%8A%A5%E5%91%8A%E7%AD%89\GDR1624-%E8%A5%BF%E5%8C%97%E5%86%9C%E6%9E%97%E7%A7%91%E6%8A%80%E5%A4%A7%E5%AD%A6%E5%8A%A8%E7%89%A9%E5%8C%BB%E5%AD%A6%E9%99%A24%E4%BE%8B%E7%9C%9F%E8%8F%8C%E8%BD%AC%E5%BD%95%E7%BB%84%E6%B5%8B%E5%BA%8F%E9%A1%B9%E7%9B%AE-Denovo-RNAseq_result\4_basic_annotation\KEGG\Trichophyton_mentagrophytes-Unigene.fa.htm#gene95) | 9 (0.39%) | ko00730 |
| 96 | [Fatty acid elongation](file:///G:\WEN\%E8%BD%AC%E5%BD%95%E7%BB%84%E6%B5%8B%E5%BA%8F\%E3%80%90%E6%89%B9%E9%87%8F%E4%B8%8B%E8%BD%BD%E3%80%91GDR1624-%E8%A5%BF%E5%8C%97%E5%86%9C%E6%9E%97%E7%A7%91%E6%8A%80%E5%A4%A7%E5%AD%A6%E5%8A%A8%E7%89%A9%E5%8C%BB%E5%AD%A6%E9%99%A24%E4%BE%8B%E7%9C%9F%E8%8F%8C%E8%BD%AC%E5%BD%95%E7%BB%84%E6%B5%8B%E5%BA%8F%E9%A1%B9%E7%9B%AE-%E5%89%94%E9%99%A4T2%E6%A0%B7%E5%93%81-Denovo_%E5%AE%8C%E6%95%B4%E7%89%88%E7%BB%93%E9%A2%98%E6%8A%A5%E5%91%8A%E7%AD%89\GDR1624-%E8%A5%BF%E5%8C%97%E5%86%9C%E6%9E%97%E7%A7%91%E6%8A%80%E5%A4%A7%E5%AD%A6%E5%8A%A8%E7%89%A9%E5%8C%BB%E5%AD%A6%E9%99%A24%E4%BE%8B%E7%9C%9F%E8%8F%8C%E8%BD%AC%E5%BD%95%E7%BB%84%E6%B5%8B%E5%BA%8F%E9%A1%B9%E7%9B%AE-Denovo-RNAseq_result\4_basic_annotation\KEGG\Trichophyton_mentagrophytes-Unigene.fa.htm#gene96) | 9 (0.39%) | ko00062 |
| 97 | [Synthesis and degradation of ketone bodies](file:///G:\WEN\%E8%BD%AC%E5%BD%95%E7%BB%84%E6%B5%8B%E5%BA%8F\%E3%80%90%E6%89%B9%E9%87%8F%E4%B8%8B%E8%BD%BD%E3%80%91GDR1624-%E8%A5%BF%E5%8C%97%E5%86%9C%E6%9E%97%E7%A7%91%E6%8A%80%E5%A4%A7%E5%AD%A6%E5%8A%A8%E7%89%A9%E5%8C%BB%E5%AD%A6%E9%99%A24%E4%BE%8B%E7%9C%9F%E8%8F%8C%E8%BD%AC%E5%BD%95%E7%BB%84%E6%B5%8B%E5%BA%8F%E9%A1%B9%E7%9B%AE-%E5%89%94%E9%99%A4T2%E6%A0%B7%E5%93%81-Denovo_%E5%AE%8C%E6%95%B4%E7%89%88%E7%BB%93%E9%A2%98%E6%8A%A5%E5%91%8A%E7%AD%89\GDR1624-%E8%A5%BF%E5%8C%97%E5%86%9C%E6%9E%97%E7%A7%91%E6%8A%80%E5%A4%A7%E5%AD%A6%E5%8A%A8%E7%89%A9%E5%8C%BB%E5%AD%A6%E9%99%A24%E4%BE%8B%E7%9C%9F%E8%8F%8C%E8%BD%AC%E5%BD%95%E7%BB%84%E6%B5%8B%E5%BA%8F%E9%A1%B9%E7%9B%AE-Denovo-RNAseq_result\4_basic_annotation\KEGG\Trichophyton_mentagrophytes-Unigene.fa.htm#gene97) | 9 (0.39%) | ko00072 |
| 98 | [Carotenoid biosynthesis](file:///G:\WEN\%E8%BD%AC%E5%BD%95%E7%BB%84%E6%B5%8B%E5%BA%8F\%E3%80%90%E6%89%B9%E9%87%8F%E4%B8%8B%E8%BD%BD%E3%80%91GDR1624-%E8%A5%BF%E5%8C%97%E5%86%9C%E6%9E%97%E7%A7%91%E6%8A%80%E5%A4%A7%E5%AD%A6%E5%8A%A8%E7%89%A9%E5%8C%BB%E5%AD%A6%E9%99%A24%E4%BE%8B%E7%9C%9F%E8%8F%8C%E8%BD%AC%E5%BD%95%E7%BB%84%E6%B5%8B%E5%BA%8F%E9%A1%B9%E7%9B%AE-%E5%89%94%E9%99%A4T2%E6%A0%B7%E5%93%81-Denovo_%E5%AE%8C%E6%95%B4%E7%89%88%E7%BB%93%E9%A2%98%E6%8A%A5%E5%91%8A%E7%AD%89\GDR1624-%E8%A5%BF%E5%8C%97%E5%86%9C%E6%9E%97%E7%A7%91%E6%8A%80%E5%A4%A7%E5%AD%A6%E5%8A%A8%E7%89%A9%E5%8C%BB%E5%AD%A6%E9%99%A24%E4%BE%8B%E7%9C%9F%E8%8F%8C%E8%BD%AC%E5%BD%95%E7%BB%84%E6%B5%8B%E5%BA%8F%E9%A1%B9%E7%9B%AE-Denovo-RNAseq_result\4_basic_annotation\KEGG\Trichophyton_mentagrophytes-Unigene.fa.htm#gene98) | 8 (0.35%) | ko00906 |
| 99 | [Vitamin B6 metabolism](file:///G:\WEN\%E8%BD%AC%E5%BD%95%E7%BB%84%E6%B5%8B%E5%BA%8F\%E3%80%90%E6%89%B9%E9%87%8F%E4%B8%8B%E8%BD%BD%E3%80%91GDR1624-%E8%A5%BF%E5%8C%97%E5%86%9C%E6%9E%97%E7%A7%91%E6%8A%80%E5%A4%A7%E5%AD%A6%E5%8A%A8%E7%89%A9%E5%8C%BB%E5%AD%A6%E9%99%A24%E4%BE%8B%E7%9C%9F%E8%8F%8C%E8%BD%AC%E5%BD%95%E7%BB%84%E6%B5%8B%E5%BA%8F%E9%A1%B9%E7%9B%AE-%E5%89%94%E9%99%A4T2%E6%A0%B7%E5%93%81-Denovo_%E5%AE%8C%E6%95%B4%E7%89%88%E7%BB%93%E9%A2%98%E6%8A%A5%E5%91%8A%E7%AD%89\GDR1624-%E8%A5%BF%E5%8C%97%E5%86%9C%E6%9E%97%E7%A7%91%E6%8A%80%E5%A4%A7%E5%AD%A6%E5%8A%A8%E7%89%A9%E5%8C%BB%E5%AD%A6%E9%99%A24%E4%BE%8B%E7%9C%9F%E8%8F%8C%E8%BD%AC%E5%BD%95%E7%BB%84%E6%B5%8B%E5%BA%8F%E9%A1%B9%E7%9B%AE-Denovo-RNAseq_result\4_basic_annotation\KEGG\Trichophyton_mentagrophytes-Unigene.fa.htm#gene99) | 7 (0.31%) | ko00750 |
| 100 | [C5-Branched dibasic acid metabolism](file:///G:\WEN\%E8%BD%AC%E5%BD%95%E7%BB%84%E6%B5%8B%E5%BA%8F\%E3%80%90%E6%89%B9%E9%87%8F%E4%B8%8B%E8%BD%BD%E3%80%91GDR1624-%E8%A5%BF%E5%8C%97%E5%86%9C%E6%9E%97%E7%A7%91%E6%8A%80%E5%A4%A7%E5%AD%A6%E5%8A%A8%E7%89%A9%E5%8C%BB%E5%AD%A6%E9%99%A24%E4%BE%8B%E7%9C%9F%E8%8F%8C%E8%BD%AC%E5%BD%95%E7%BB%84%E6%B5%8B%E5%BA%8F%E9%A1%B9%E7%9B%AE-%E5%89%94%E9%99%A4T2%E6%A0%B7%E5%93%81-Denovo_%E5%AE%8C%E6%95%B4%E7%89%88%E7%BB%93%E9%A2%98%E6%8A%A5%E5%91%8A%E7%AD%89\GDR1624-%E8%A5%BF%E5%8C%97%E5%86%9C%E6%9E%97%E7%A7%91%E6%8A%80%E5%A4%A7%E5%AD%A6%E5%8A%A8%E7%89%A9%E5%8C%BB%E5%AD%A6%E9%99%A24%E4%BE%8B%E7%9C%9F%E8%8F%8C%E8%BD%AC%E5%BD%95%E7%BB%84%E6%B5%8B%E5%BA%8F%E9%A1%B9%E7%9B%AE-Denovo-RNAseq_result\4_basic_annotation\KEGG\Trichophyton_mentagrophytes-Unigene.fa.htm#gene100) | 7 (0.31%) | ko00660 |
| 101 | [Other glycan degradation](file:///G:\WEN\%E8%BD%AC%E5%BD%95%E7%BB%84%E6%B5%8B%E5%BA%8F\%E3%80%90%E6%89%B9%E9%87%8F%E4%B8%8B%E8%BD%BD%E3%80%91GDR1624-%E8%A5%BF%E5%8C%97%E5%86%9C%E6%9E%97%E7%A7%91%E6%8A%80%E5%A4%A7%E5%AD%A6%E5%8A%A8%E7%89%A9%E5%8C%BB%E5%AD%A6%E9%99%A24%E4%BE%8B%E7%9C%9F%E8%8F%8C%E8%BD%AC%E5%BD%95%E7%BB%84%E6%B5%8B%E5%BA%8F%E9%A1%B9%E7%9B%AE-%E5%89%94%E9%99%A4T2%E6%A0%B7%E5%93%81-Denovo_%E5%AE%8C%E6%95%B4%E7%89%88%E7%BB%93%E9%A2%98%E6%8A%A5%E5%91%8A%E7%AD%89\GDR1624-%E8%A5%BF%E5%8C%97%E5%86%9C%E6%9E%97%E7%A7%91%E6%8A%80%E5%A4%A7%E5%AD%A6%E5%8A%A8%E7%89%A9%E5%8C%BB%E5%AD%A6%E9%99%A24%E4%BE%8B%E7%9C%9F%E8%8F%8C%E8%BD%AC%E5%BD%95%E7%BB%84%E6%B5%8B%E5%BA%8F%E9%A1%B9%E7%9B%AE-Denovo-RNAseq_result\4_basic_annotation\KEGG\Trichophyton_mentagrophytes-Unigene.fa.htm#gene101) | 7 (0.31%) | ko00511 |
| 102 | [Degradation of aromatic compounds](file:///G:\WEN\%E8%BD%AC%E5%BD%95%E7%BB%84%E6%B5%8B%E5%BA%8F\%E3%80%90%E6%89%B9%E9%87%8F%E4%B8%8B%E8%BD%BD%E3%80%91GDR1624-%E8%A5%BF%E5%8C%97%E5%86%9C%E6%9E%97%E7%A7%91%E6%8A%80%E5%A4%A7%E5%AD%A6%E5%8A%A8%E7%89%A9%E5%8C%BB%E5%AD%A6%E9%99%A24%E4%BE%8B%E7%9C%9F%E8%8F%8C%E8%BD%AC%E5%BD%95%E7%BB%84%E6%B5%8B%E5%BA%8F%E9%A1%B9%E7%9B%AE-%E5%89%94%E9%99%A4T2%E6%A0%B7%E5%93%81-Denovo_%E5%AE%8C%E6%95%B4%E7%89%88%E7%BB%93%E9%A2%98%E6%8A%A5%E5%91%8A%E7%AD%89\GDR1624-%E8%A5%BF%E5%8C%97%E5%86%9C%E6%9E%97%E7%A7%91%E6%8A%80%E5%A4%A7%E5%AD%A6%E5%8A%A8%E7%89%A9%E5%8C%BB%E5%AD%A6%E9%99%A24%E4%BE%8B%E7%9C%9F%E8%8F%8C%E8%BD%AC%E5%BD%95%E7%BB%84%E6%B5%8B%E5%BA%8F%E9%A1%B9%E7%9B%AE-Denovo-RNAseq_result\4_basic_annotation\KEGG\Trichophyton_mentagrophytes-Unigene.fa.htm#gene102) | 6 (0.26%) | ko01220 |
| 103 | [Penicillin and cephalosporin biosynthesis](file:///G:\WEN\%E8%BD%AC%E5%BD%95%E7%BB%84%E6%B5%8B%E5%BA%8F\%E3%80%90%E6%89%B9%E9%87%8F%E4%B8%8B%E8%BD%BD%E3%80%91GDR1624-%E8%A5%BF%E5%8C%97%E5%86%9C%E6%9E%97%E7%A7%91%E6%8A%80%E5%A4%A7%E5%AD%A6%E5%8A%A8%E7%89%A9%E5%8C%BB%E5%AD%A6%E9%99%A24%E4%BE%8B%E7%9C%9F%E8%8F%8C%E8%BD%AC%E5%BD%95%E7%BB%84%E6%B5%8B%E5%BA%8F%E9%A1%B9%E7%9B%AE-%E5%89%94%E9%99%A4T2%E6%A0%B7%E5%93%81-Denovo_%E5%AE%8C%E6%95%B4%E7%89%88%E7%BB%93%E9%A2%98%E6%8A%A5%E5%91%8A%E7%AD%89\GDR1624-%E8%A5%BF%E5%8C%97%E5%86%9C%E6%9E%97%E7%A7%91%E6%8A%80%E5%A4%A7%E5%AD%A6%E5%8A%A8%E7%89%A9%E5%8C%BB%E5%AD%A6%E9%99%A24%E4%BE%8B%E7%9C%9F%E8%8F%8C%E8%BD%AC%E5%BD%95%E7%BB%84%E6%B5%8B%E5%BA%8F%E9%A1%B9%E7%9B%AE-Denovo-RNAseq_result\4_basic_annotation\KEGG\Trichophyton_mentagrophytes-Unigene.fa.htm#gene103) | 6 (0.26%) | ko00311 |
| 104 | [alpha-Linolenic acid metabolism](file:///G:\WEN\%E8%BD%AC%E5%BD%95%E7%BB%84%E6%B5%8B%E5%BA%8F\%E3%80%90%E6%89%B9%E9%87%8F%E4%B8%8B%E8%BD%BD%E3%80%91GDR1624-%E8%A5%BF%E5%8C%97%E5%86%9C%E6%9E%97%E7%A7%91%E6%8A%80%E5%A4%A7%E5%AD%A6%E5%8A%A8%E7%89%A9%E5%8C%BB%E5%AD%A6%E9%99%A24%E4%BE%8B%E7%9C%9F%E8%8F%8C%E8%BD%AC%E5%BD%95%E7%BB%84%E6%B5%8B%E5%BA%8F%E9%A1%B9%E7%9B%AE-%E5%89%94%E9%99%A4T2%E6%A0%B7%E5%93%81-Denovo_%E5%AE%8C%E6%95%B4%E7%89%88%E7%BB%93%E9%A2%98%E6%8A%A5%E5%91%8A%E7%AD%89\GDR1624-%E8%A5%BF%E5%8C%97%E5%86%9C%E6%9E%97%E7%A7%91%E6%8A%80%E5%A4%A7%E5%AD%A6%E5%8A%A8%E7%89%A9%E5%8C%BB%E5%AD%A6%E9%99%A24%E4%BE%8B%E7%9C%9F%E8%8F%8C%E8%BD%AC%E5%BD%95%E7%BB%84%E6%B5%8B%E5%BA%8F%E9%A1%B9%E7%9B%AE-Denovo-RNAseq_result\4_basic_annotation\KEGG\Trichophyton_mentagrophytes-Unigene.fa.htm#gene104) | 6 (0.26%) | ko00592 |
| 105 | [Other types of O-glycan biosynthesis](file:///G:\WEN\%E8%BD%AC%E5%BD%95%E7%BB%84%E6%B5%8B%E5%BA%8F\%E3%80%90%E6%89%B9%E9%87%8F%E4%B8%8B%E8%BD%BD%E3%80%91GDR1624-%E8%A5%BF%E5%8C%97%E5%86%9C%E6%9E%97%E7%A7%91%E6%8A%80%E5%A4%A7%E5%AD%A6%E5%8A%A8%E7%89%A9%E5%8C%BB%E5%AD%A6%E9%99%A24%E4%BE%8B%E7%9C%9F%E8%8F%8C%E8%BD%AC%E5%BD%95%E7%BB%84%E6%B5%8B%E5%BA%8F%E9%A1%B9%E7%9B%AE-%E5%89%94%E9%99%A4T2%E6%A0%B7%E5%93%81-Denovo_%E5%AE%8C%E6%95%B4%E7%89%88%E7%BB%93%E9%A2%98%E6%8A%A5%E5%91%8A%E7%AD%89\GDR1624-%E8%A5%BF%E5%8C%97%E5%86%9C%E6%9E%97%E7%A7%91%E6%8A%80%E5%A4%A7%E5%AD%A6%E5%8A%A8%E7%89%A9%E5%8C%BB%E5%AD%A6%E9%99%A24%E4%BE%8B%E7%9C%9F%E8%8F%8C%E8%BD%AC%E5%BD%95%E7%BB%84%E6%B5%8B%E5%BA%8F%E9%A1%B9%E7%9B%AE-Denovo-RNAseq_result\4_basic_annotation\KEGG\Trichophyton_mentagrophytes-Unigene.fa.htm#gene105) | 5 (0.22%) | ko00514 |
| 106 | [Riboflavin metabolism](file:///G:\WEN\%E8%BD%AC%E5%BD%95%E7%BB%84%E6%B5%8B%E5%BA%8F\%E3%80%90%E6%89%B9%E9%87%8F%E4%B8%8B%E8%BD%BD%E3%80%91GDR1624-%E8%A5%BF%E5%8C%97%E5%86%9C%E6%9E%97%E7%A7%91%E6%8A%80%E5%A4%A7%E5%AD%A6%E5%8A%A8%E7%89%A9%E5%8C%BB%E5%AD%A6%E9%99%A24%E4%BE%8B%E7%9C%9F%E8%8F%8C%E8%BD%AC%E5%BD%95%E7%BB%84%E6%B5%8B%E5%BA%8F%E9%A1%B9%E7%9B%AE-%E5%89%94%E9%99%A4T2%E6%A0%B7%E5%93%81-Denovo_%E5%AE%8C%E6%95%B4%E7%89%88%E7%BB%93%E9%A2%98%E6%8A%A5%E5%91%8A%E7%AD%89\GDR1624-%E8%A5%BF%E5%8C%97%E5%86%9C%E6%9E%97%E7%A7%91%E6%8A%80%E5%A4%A7%E5%AD%A6%E5%8A%A8%E7%89%A9%E5%8C%BB%E5%AD%A6%E9%99%A24%E4%BE%8B%E7%9C%9F%E8%8F%8C%E8%BD%AC%E5%BD%95%E7%BB%84%E6%B5%8B%E5%BA%8F%E9%A1%B9%E7%9B%AE-Denovo-RNAseq_result\4_basic_annotation\KEGG\Trichophyton_mentagrophytes-Unigene.fa.htm#gene106) | 5 (0.22%) | ko00740 |
| 107 | [Arachidonic acid metabolism](file:///G:\WEN\%E8%BD%AC%E5%BD%95%E7%BB%84%E6%B5%8B%E5%BA%8F\%E3%80%90%E6%89%B9%E9%87%8F%E4%B8%8B%E8%BD%BD%E3%80%91GDR1624-%E8%A5%BF%E5%8C%97%E5%86%9C%E6%9E%97%E7%A7%91%E6%8A%80%E5%A4%A7%E5%AD%A6%E5%8A%A8%E7%89%A9%E5%8C%BB%E5%AD%A6%E9%99%A24%E4%BE%8B%E7%9C%9F%E8%8F%8C%E8%BD%AC%E5%BD%95%E7%BB%84%E6%B5%8B%E5%BA%8F%E9%A1%B9%E7%9B%AE-%E5%89%94%E9%99%A4T2%E6%A0%B7%E5%93%81-Denovo_%E5%AE%8C%E6%95%B4%E7%89%88%E7%BB%93%E9%A2%98%E6%8A%A5%E5%91%8A%E7%AD%89\GDR1624-%E8%A5%BF%E5%8C%97%E5%86%9C%E6%9E%97%E7%A7%91%E6%8A%80%E5%A4%A7%E5%AD%A6%E5%8A%A8%E7%89%A9%E5%8C%BB%E5%AD%A6%E9%99%A24%E4%BE%8B%E7%9C%9F%E8%8F%8C%E8%BD%AC%E5%BD%95%E7%BB%84%E6%B5%8B%E5%BA%8F%E9%A1%B9%E7%9B%AE-Denovo-RNAseq_result\4_basic_annotation\KEGG\Trichophyton_mentagrophytes-Unigene.fa.htm#gene107) | 5 (0.22%) | ko00590 |
| 108 | [Ascorbate and aldarate metabolism](file:///G:\WEN\%E8%BD%AC%E5%BD%95%E7%BB%84%E6%B5%8B%E5%BA%8F\%E3%80%90%E6%89%B9%E9%87%8F%E4%B8%8B%E8%BD%BD%E3%80%91GDR1624-%E8%A5%BF%E5%8C%97%E5%86%9C%E6%9E%97%E7%A7%91%E6%8A%80%E5%A4%A7%E5%AD%A6%E5%8A%A8%E7%89%A9%E5%8C%BB%E5%AD%A6%E9%99%A24%E4%BE%8B%E7%9C%9F%E8%8F%8C%E8%BD%AC%E5%BD%95%E7%BB%84%E6%B5%8B%E5%BA%8F%E9%A1%B9%E7%9B%AE-%E5%89%94%E9%99%A4T2%E6%A0%B7%E5%93%81-Denovo_%E5%AE%8C%E6%95%B4%E7%89%88%E7%BB%93%E9%A2%98%E6%8A%A5%E5%91%8A%E7%AD%89\GDR1624-%E8%A5%BF%E5%8C%97%E5%86%9C%E6%9E%97%E7%A7%91%E6%8A%80%E5%A4%A7%E5%AD%A6%E5%8A%A8%E7%89%A9%E5%8C%BB%E5%AD%A6%E9%99%A24%E4%BE%8B%E7%9C%9F%E8%8F%8C%E8%BD%AC%E5%BD%95%E7%BB%84%E6%B5%8B%E5%BA%8F%E9%A1%B9%E7%9B%AE-Denovo-RNAseq_result\4_basic_annotation\KEGG\Trichophyton_mentagrophytes-Unigene.fa.htm#gene108) | 5 (0.22%) | ko00053 |
| 109 | [Caffeine metabolism](file:///G:\WEN\%E8%BD%AC%E5%BD%95%E7%BB%84%E6%B5%8B%E5%BA%8F\%E3%80%90%E6%89%B9%E9%87%8F%E4%B8%8B%E8%BD%BD%E3%80%91GDR1624-%E8%A5%BF%E5%8C%97%E5%86%9C%E6%9E%97%E7%A7%91%E6%8A%80%E5%A4%A7%E5%AD%A6%E5%8A%A8%E7%89%A9%E5%8C%BB%E5%AD%A6%E9%99%A24%E4%BE%8B%E7%9C%9F%E8%8F%8C%E8%BD%AC%E5%BD%95%E7%BB%84%E6%B5%8B%E5%BA%8F%E9%A1%B9%E7%9B%AE-%E5%89%94%E9%99%A4T2%E6%A0%B7%E5%93%81-Denovo_%E5%AE%8C%E6%95%B4%E7%89%88%E7%BB%93%E9%A2%98%E6%8A%A5%E5%91%8A%E7%AD%89\GDR1624-%E8%A5%BF%E5%8C%97%E5%86%9C%E6%9E%97%E7%A7%91%E6%8A%80%E5%A4%A7%E5%AD%A6%E5%8A%A8%E7%89%A9%E5%8C%BB%E5%AD%A6%E9%99%A24%E4%BE%8B%E7%9C%9F%E8%8F%8C%E8%BD%AC%E5%BD%95%E7%BB%84%E6%B5%8B%E5%BA%8F%E9%A1%B9%E7%9B%AE-Denovo-RNAseq_result\4_basic_annotation\KEGG\Trichophyton_mentagrophytes-Unigene.fa.htm#gene109) | 3 (0.13%) | ko00232 |
| 110 | [Monobactam biosynthesis](file:///G:\WEN\%E8%BD%AC%E5%BD%95%E7%BB%84%E6%B5%8B%E5%BA%8F\%E3%80%90%E6%89%B9%E9%87%8F%E4%B8%8B%E8%BD%BD%E3%80%91GDR1624-%E8%A5%BF%E5%8C%97%E5%86%9C%E6%9E%97%E7%A7%91%E6%8A%80%E5%A4%A7%E5%AD%A6%E5%8A%A8%E7%89%A9%E5%8C%BB%E5%AD%A6%E9%99%A24%E4%BE%8B%E7%9C%9F%E8%8F%8C%E8%BD%AC%E5%BD%95%E7%BB%84%E6%B5%8B%E5%BA%8F%E9%A1%B9%E7%9B%AE-%E5%89%94%E9%99%A4T2%E6%A0%B7%E5%93%81-Denovo_%E5%AE%8C%E6%95%B4%E7%89%88%E7%BB%93%E9%A2%98%E6%8A%A5%E5%91%8A%E7%AD%89\GDR1624-%E8%A5%BF%E5%8C%97%E5%86%9C%E6%9E%97%E7%A7%91%E6%8A%80%E5%A4%A7%E5%AD%A6%E5%8A%A8%E7%89%A9%E5%8C%BB%E5%AD%A6%E9%99%A24%E4%BE%8B%E7%9C%9F%E8%8F%8C%E8%BD%AC%E5%BD%95%E7%BB%84%E6%B5%8B%E5%BA%8F%E9%A1%B9%E7%9B%AE-Denovo-RNAseq_result\4_basic_annotation\KEGG\Trichophyton_mentagrophytes-Unigene.fa.htm#gene110) | 3 (0.13%) | ko00261 |
| 111 | [Lipoic acid metabolism](file:///G:\WEN\%E8%BD%AC%E5%BD%95%E7%BB%84%E6%B5%8B%E5%BA%8F\%E3%80%90%E6%89%B9%E9%87%8F%E4%B8%8B%E8%BD%BD%E3%80%91GDR1624-%E8%A5%BF%E5%8C%97%E5%86%9C%E6%9E%97%E7%A7%91%E6%8A%80%E5%A4%A7%E5%AD%A6%E5%8A%A8%E7%89%A9%E5%8C%BB%E5%AD%A6%E9%99%A24%E4%BE%8B%E7%9C%9F%E8%8F%8C%E8%BD%AC%E5%BD%95%E7%BB%84%E6%B5%8B%E5%BA%8F%E9%A1%B9%E7%9B%AE-%E5%89%94%E9%99%A4T2%E6%A0%B7%E5%93%81-Denovo_%E5%AE%8C%E6%95%B4%E7%89%88%E7%BB%93%E9%A2%98%E6%8A%A5%E5%91%8A%E7%AD%89\GDR1624-%E8%A5%BF%E5%8C%97%E5%86%9C%E6%9E%97%E7%A7%91%E6%8A%80%E5%A4%A7%E5%AD%A6%E5%8A%A8%E7%89%A9%E5%8C%BB%E5%AD%A6%E9%99%A24%E4%BE%8B%E7%9C%9F%E8%8F%8C%E8%BD%AC%E5%BD%95%E7%BB%84%E6%B5%8B%E5%BA%8F%E9%A1%B9%E7%9B%AE-Denovo-RNAseq_result\4_basic_annotation\KEGG\Trichophyton_mentagrophytes-Unigene.fa.htm#gene111) | 3 (0.13%) | ko00785 |
| 112 | [Carbapenem biosynthesis](file:///G:\WEN\%E8%BD%AC%E5%BD%95%E7%BB%84%E6%B5%8B%E5%BA%8F\%E3%80%90%E6%89%B9%E9%87%8F%E4%B8%8B%E8%BD%BD%E3%80%91GDR1624-%E8%A5%BF%E5%8C%97%E5%86%9C%E6%9E%97%E7%A7%91%E6%8A%80%E5%A4%A7%E5%AD%A6%E5%8A%A8%E7%89%A9%E5%8C%BB%E5%AD%A6%E9%99%A24%E4%BE%8B%E7%9C%9F%E8%8F%8C%E8%BD%AC%E5%BD%95%E7%BB%84%E6%B5%8B%E5%BA%8F%E9%A1%B9%E7%9B%AE-%E5%89%94%E9%99%A4T2%E6%A0%B7%E5%93%81-Denovo_%E5%AE%8C%E6%95%B4%E7%89%88%E7%BB%93%E9%A2%98%E6%8A%A5%E5%91%8A%E7%AD%89\GDR1624-%E8%A5%BF%E5%8C%97%E5%86%9C%E6%9E%97%E7%A7%91%E6%8A%80%E5%A4%A7%E5%AD%A6%E5%8A%A8%E7%89%A9%E5%8C%BB%E5%AD%A6%E9%99%A24%E4%BE%8B%E7%9C%9F%E8%8F%8C%E8%BD%AC%E5%BD%95%E7%BB%84%E6%B5%8B%E5%BA%8F%E9%A1%B9%E7%9B%AE-Denovo-RNAseq_result\4_basic_annotation\KEGG\Trichophyton_mentagrophytes-Unigene.fa.htm#gene112) | 3 (0.13%) | ko00332 |
| 113 | [Sesquiterpenoid and triterpenoid biosynthesis](file:///G:\WEN\%E8%BD%AC%E5%BD%95%E7%BB%84%E6%B5%8B%E5%BA%8F\%E3%80%90%E6%89%B9%E9%87%8F%E4%B8%8B%E8%BD%BD%E3%80%91GDR1624-%E8%A5%BF%E5%8C%97%E5%86%9C%E6%9E%97%E7%A7%91%E6%8A%80%E5%A4%A7%E5%AD%A6%E5%8A%A8%E7%89%A9%E5%8C%BB%E5%AD%A6%E9%99%A24%E4%BE%8B%E7%9C%9F%E8%8F%8C%E8%BD%AC%E5%BD%95%E7%BB%84%E6%B5%8B%E5%BA%8F%E9%A1%B9%E7%9B%AE-%E5%89%94%E9%99%A4T2%E6%A0%B7%E5%93%81-Denovo_%E5%AE%8C%E6%95%B4%E7%89%88%E7%BB%93%E9%A2%98%E6%8A%A5%E5%91%8A%E7%AD%89\GDR1624-%E8%A5%BF%E5%8C%97%E5%86%9C%E6%9E%97%E7%A7%91%E6%8A%80%E5%A4%A7%E5%AD%A6%E5%8A%A8%E7%89%A9%E5%8C%BB%E5%AD%A6%E9%99%A24%E4%BE%8B%E7%9C%9F%E8%8F%8C%E8%BD%AC%E5%BD%95%E7%BB%84%E6%B5%8B%E5%BA%8F%E9%A1%B9%E7%9B%AE-Denovo-RNAseq_result\4_basic_annotation\KEGG\Trichophyton_mentagrophytes-Unigene.fa.htm#gene113) | 2 (0.09%) | ko00909 |
| 114 | [D-Arginine and D-ornithine metabolism](file:///G:\WEN\%E8%BD%AC%E5%BD%95%E7%BB%84%E6%B5%8B%E5%BA%8F\%E3%80%90%E6%89%B9%E9%87%8F%E4%B8%8B%E8%BD%BD%E3%80%91GDR1624-%E8%A5%BF%E5%8C%97%E5%86%9C%E6%9E%97%E7%A7%91%E6%8A%80%E5%A4%A7%E5%AD%A6%E5%8A%A8%E7%89%A9%E5%8C%BB%E5%AD%A6%E9%99%A24%E4%BE%8B%E7%9C%9F%E8%8F%8C%E8%BD%AC%E5%BD%95%E7%BB%84%E6%B5%8B%E5%BA%8F%E9%A1%B9%E7%9B%AE-%E5%89%94%E9%99%A4T2%E6%A0%B7%E5%93%81-Denovo_%E5%AE%8C%E6%95%B4%E7%89%88%E7%BB%93%E9%A2%98%E6%8A%A5%E5%91%8A%E7%AD%89\GDR1624-%E8%A5%BF%E5%8C%97%E5%86%9C%E6%9E%97%E7%A7%91%E6%8A%80%E5%A4%A7%E5%AD%A6%E5%8A%A8%E7%89%A9%E5%8C%BB%E5%AD%A6%E9%99%A24%E4%BE%8B%E7%9C%9F%E8%8F%8C%E8%BD%AC%E5%BD%95%E7%BB%84%E6%B5%8B%E5%BA%8F%E9%A1%B9%E7%9B%AE-Denovo-RNAseq_result\4_basic_annotation\KEGG\Trichophyton_mentagrophytes-Unigene.fa.htm#gene114) | 2 (0.09%) | ko00472 |
