# Supplementary material for: Transcriptome sequencing and analysis of zinc-uptake-related genes in Trichophyton mentagrophytes
Source: BMC Genomics. 2017 Nov 21;18:888. doi: 10.1186/s12864-017-4284-3 (PMC5697147; doi:10.1186/s12864-017-4284-3)
Supplement: Supplementary file 8 — The unigenes that have zinc finger structure. (DOCX 16 kb) [file 12864_2017_4284_MOESM8_ESM.docx]

|  | Description |
| --- | --- |
| Unigene0008014 | zinc-responsiveness transcriptional activator [*Trichophyton equinum* CBS 127.97] |
| Unigene0003597 | zinc finger protein 58 [*Trichophyton tonsurans* CBS 112818] |
| Unigene0000651 | zinc finger protein 32 [*Microsporum gypseum* CBS 118893] |
| Unigene0000648 | C2H2 zinc finger protein [*Arthroderma benhamiae* CBS 112371] |
| Unigene0007211 | zinc finger protein 273 [*Microsporum gypseum* CBS 118893] |
| Unigene0010738 | C2H2 finger domain-containing protein [*Trichophyton rubrum* CBS 118892] |
| Unigene0010737 | C2H2 finger domain-containing protein [*Trichophyton tonsurans* CBS 112818] |
| Unigene0000414 | C2H2 transcription factor [*Trichophyton rubrum* CBS 118892] |
| Unigene0005193 | C2H2 finger domain-containing protein [*Trichophyton tonsurans* CBS 112818] |
| Unigene0006034 | C2H2 transcription factor [*Trichophyton equinum* CBS 127.97] |
| Unigene0000640 | C2H2 transcription factor [*Trichophyton equinum* CBS 127.97] |
| Unigene0005062 | C2H2 transcription factor [*Trichophyton rubrum* CBS 118892] |
| Unigene0000227 | C2H2 finger domain-containing protein [*Trichophyton equinum* CBS 127.97] |
| Unigene0006172 | C2H2 transcription factor [*Trichophyton tonsurans* CBS 112818] |
| Unigene0010002 | C2H2 transcription factor [*Trichophyton tonsurans* CBS 112818] |
| Unigene0002887 | zinc finger transcription factor [*Trichophyton equinum* CBS 127.97] |
| Unigene0002886 | zinc finger transcription factor [*Trichophyton equinum* CBS 127.97] |
| Unigene0005665 | C2H2 finger domain-containing protein [*Trichophyton tonsurans* CBS 112818] |
| Unigene0002868 | C2H2 type zinc finger domain protein [*Aspergillus oryzae* RIB40] |
| Unigene0002870 | C2H2 type zinc finger domain protein [*Aspergillus oryzae* RIB40] |
| Unigene0002097 | Zinc finger protein SFP1 [*Trichophyton tonsurans* CBS 112818] |
| Unigene0002096 | Zinc finger protein SFP1 [*Trichophyton tonsurans* CBS 112818] |
| Unigene0004954 | C2H2 finger domain-containing protein [*Trichophyton tonsurans* CBS 112818] |
| Unigene0005234 | C2H2 type zinc finger containing protein [*Coccidioides posadasii* C735 delta SOWgp] |
| Unigene0000329 | C2H2 finger domain-containing protein [*Blastomyces dermatitidis* ER-3] |
| Unigene0004213 | Zinc finger C2H2-type/integrase DNA-binding domain [*Trichophyton rubrum]* |
| Unigene0004778 | C2H2 transcription factor [*Trichophyton tonsurans* CBS 112818] |
