# Supplementary material for: Transcriptome sequencing and analysis of zinc-uptake-related genes in Trichophyton mentagrophytes
Source: BMC Genomics. 2017 Nov 21;18:888. doi: 10.1186/s12864-017-4284-3 (PMC5697147; doi:10.1186/s12864-017-4284-3)
Supplement: Supplementary file 9 — Three pairs of primers that can amplify zinc-responsive. (DOCX 15 kb) [file 12864_2017_4284_MOESM9_ESM.docx]

| 1-F | CAGTCAGCCACAGTCTTG |
| --- | --- |
| 1-R | CCTTAGCACCTTCAGTCTG |
| 2-F | ACCTTAATCACCTTCACCAA |
| 2-R | GATTCAAGTCCGCAATAGTG |
| 3-F | CCTTACACTTACATCCTCCAT |
| 3-R | CTATCAGCGAACTATCTACTCT |

The three pairs primers of Unigene0008014
