# Supplementary material for: Transcriptome sequencing and analysis of zinc-uptake-related genes in Trichophyton mentagrophytes
Source: BMC Genomics. 2017 Nov 21;18:888. doi: 10.1186/s12864-017-4284-3 (PMC5697147; doi:10.1186/s12864-017-4284-3)
Supplement: Supplementary file 10 — The qRT-PCR differential expression analyses. (DOCX 17 kb) [file 12864_2017_4284_MOESM10_ESM.docx]

| Unigene0002709 | CG 1 | CG 2 | CG 3 | EG 1 | EG 2 | EG 3 |
| --- | --- | --- | --- | --- | --- | --- |
| 2^-ΔΔct | 1 | 1.389088 | 1.631254 | 0.059484 | 0.06358 | 0.070823 |
| STD | 0.038291 | 0.058519 | 0.031521 | 0.001805 | 0.002648 | 0.007034 |
| Unigene0002593 | CG 1 | CG 2 | CG 3 | EG 1 | EG 2 | EG 3 |
| 2^-ΔΔct | 1 | 1.372933 | 1.657175 | 0.462343 | 0.424541 | 0.410776 |
| STD | 0.058101 | 0.025231 | 0.012535 | 0.035686 | 0.04871 | 0.0399 |
| Unigene0002886 | CG 1 | CG 2 | CG 3 | EG 1 | EG 2 | EG 3 |
| 2^-ΔΔct | 1 | 0.921703 | 1.238336 | 0.673052 | 0.775202 | 0.750191 |
| STD | 0.046217 | 0.027197 | 0.048389 | 0.031983 | 0.021869 | 0.033447 |
| Unigene0005062 | CG 1 | CG 2 | CG 3 | EG 1 | EG 2 | EG 3 |
| 2^-ΔΔct | 1 | 0.97127 | 1.477123 | 0.242284 | 0.306229 | 0.373013 |
| STD | 0.006824 | 0.004548 | 0.016605 | 0.073272 | 0.086818 | 0.006782 |
| Unigene0008014 | CG 1 | CG 2 | CG 3 | EG 1 | EG 2 | EG 3 |
| 2^-ΔΔct | 1 | 1.297416 | 1.674534 | 0.051602 | 0.063593 | 0.064696 |
| STD | 0.049896 | 0.012997 | 0.016961 | 0.00693 | 0.0122 | 0.000289 |
| Unigene0005193 | CG 1 | CG 2 | CG 3 | EG 1 | EG 2 | EG 3 |
| 2^-ΔΔct | 1 | 1.492012 | 1.767444 | 4.742793 | 5.525754 | 4.973438 |
| STD | 0.053169 | 0.066913 | 0.061291 | 0.050382 | 0.059227 | 0.106991 |
